# Face shape and face identity processing in behavioral variant fronto-temporal dementia: A specific deficit for familiarity and name recognition of famous faces

**DOI:** 10.1016/j.nicl.2016.03.001

**Published:** 2016-03-10

**Authors:** François-Laurent De Winter, Dorien Timmers, Beatrice de Gelder, Marc Van Orshoven, Marleen Vieren, Miriam Bouckaert, Gert Cypers, Jo Caekebeke, Laura Van de Vliet, Karolien Goffin, Koen Van Laere, Stefan Sunaert, Rik Vandenberghe, Mathieu Vandenbulcke, Jan Van den Stock

**Affiliations:** a Laboratory for Translational Neuropsychiatry, Research Group Psychiatry, Department of Neurosciences, KU Leuven, Leuven, Belgium; b Department of Old Age Psychiatry, University Hospitals Leuven, Leuven, Belgium; c Department of Neurology, Onze-Lieve-Vrouwziekenhuis Aalst-Asse-Ninove, Aalst, Belgium; d Cognitive Neuroscience, Faculty of Psychology and Neuroscience, Maastricht University, Maastricht, The Netherlands; e Nuclear Medicine and Molecular Imaging, Department of Imaging and Pathology, KU Leuven, Leuven, Belgium; f Translational MRI, Department of Imaging and Pathology, KU Leuven, Leuven, Belgium; g Laboratory for Cognitive Neurology, Department of Neurosciences, KU Leuven, & Department of Neurology, University Hospitals Leuven, Leuven, Belgium

## Abstract

Deficits in face processing have been described in the behavioral variant of fronto-temporal dementia (bvFTD), primarily regarding the recognition of facial expressions. Less is known about face shape and face identity processing. Here we used a hierarchical strategy targeting face shape and face identity recognition in bvFTD and matched healthy controls. Participants performed 3 psychophysical experiments targeting face shape detection (Experiment 1), unfamiliar face identity matching (Experiment 2), familiarity categorization and famous face-name matching (Experiment 3). The results revealed group differences only in Experiment 3, with a deficit in the bvFTD group for both familiarity categorization and famous face-name matching. Voxel-based morphometry regression analyses in the bvFTD group revealed an association between grey matter volume of the left ventral anterior temporal lobe and familiarity recognition, while face-name matching correlated with grey matter volume of the bilateral ventral anterior temporal lobes. Subsequently, we quantified familiarity-specific and name-specific recognition deficits as the sum of the celebrities of which respectively only the name or only the familiarity was accurately recognized. Both indices were associated with grey matter volume of the bilateral anterior temporal cortices. These findings extent previous results by documenting the involvement of the left anterior temporal lobe (ATL) in familiarity detection and the right ATL in name recognition deficits in fronto-temporal lobar degeneration.

## Highlights

• Face outline and face identity recognition was tested in behavioral variant FTD
• Deficits were observed in recognition of famous but not unfamiliar faces
• The deficit comprised both familiarity and name recognition
• Both deficits related to structural integrity of bilateral anterio-temporal cortex

## 1 Introduction

Fronto-temporal lobar degeneration (Neary et al., 1998) is a neurodegenerative disorder associated with atrophy of the temporal and/or frontal lobes. The main regions of brain atrophy are often responsible for the corresponding symptoms. Patients with such brain atrophy can present with behavioral symptoms - designated behavioral variant fronto-temporal dementia (Rascovsky et al., 2011) - or language deficits - designated primary progressive aphasia (Gorno-Tempini et al., 2011). The former is characterized by progressive deterioration of personality, behavior and cognition, with atrophy situated in the anterior temporal, mesio-frontal and subcortical areas (Seeley et al., 2008, Whitwell et al., 2009). Neuropsychological deficits include the recognition of emotional expressions, which have primarily been consistently documented in the face domain (Baez et al., 2014, Bediou et al., 2009, Bertoux et al., 2014, Bertoux et al., 2012a, Bertoux et al., 2012b, Couto et al., 2013, Diehl-Schmid et al., 2007, Fernandez-Duque and Black, 2005, Hsieh et al., 2013, Keane et al., 2002, Kipps et al., 2009, Kumfor et al., 2014b, Kumfor et al., 2011, Kumfor and Piguet, 2012, Lavenu et al., 1999, Lough et al., 2006, Miller et al., 2012, Oliver et al., 2014, Omar et al., 2011a, Omar et al., 2011b, Rosen et al., 2004, Rosen et al., 2002, Snowden et al., 2008).

Patients with behavioral variant fronto-temporal dementia at times demonstrate decreased ability to detect the emotions conveyed by facial expression despite their ability to recognize the person as familiar (Couto et al., 2013, Keane et al., 2002, Rosen et al., 2004, Rosen et al., 2002, Snowden et al., 2008). However, studies also find decreased ability to recognize faces - so-called deficits in facial identity processing (Kumfor et al., 2015, Miller et al., 2012).

The study by Kumfor et al. (2015) further suggested that impaired face identity discrimination is associated with atrophy in the left temporal cortex, including the fusiform gyrus.

Recognition of famous faces can be considered a variant of face identity processing, combining processing of familiarity, an essential feature of famous face recognition (Bobes et al., 2013, Burton and Jenkins, 2011). There is evidence that the capacity for recognizing famous faces is impaired in fronto-temporal lobar degeneration, particularly in the semantic variant of primary progressive aphasia (Gefen et al., 2013, Gorno-Tempini et al., 2004, Snowden et al., 2004). The clinical phenotype of semantic variant primary progressive aphasia includes the loss of conceptual knowledge and is neuro-anatomically associated with anterior temporal lobe (ATL) atrophy. In line with this, patients with temporal variant fronto-temporal lobar degeneration display worse famous face identification compared to frontal variant fronto-temporal lobar degeneration (Omar, Rohrer, Hailstone, and Warren, 2011). In primary progressive aphasia, left anterior temporal grey matter volume (GMv) correlates with the ability to name faces and bilateral anterior temporal GMv correlates with recognition of famous faces (Gefen et al., 2013). These results complement earlier findings of famous person knowledge in semantic variant primary progressive aphasia, with primarily visuo-pictorial deficits in right lateralized semantic variant primary progressive aphasia and verbal deficits in left lateralized semantic variant primary progressive aphasia (Snowden et al., 2012).

The picture emerging from the findings reported above consists of a possible deficit in face identity processing in behavioral variant fronto-temporal dementia as well as a deficit in famous face recognition in semantic variant primary progressive aphasia, associated with anterior temporal grey matter volume. However, little is known about famous face recognition in behavioral variant fronto-temporal dementia, nor about the association between unfamiliar facial identity processing and famous face recognition. In the present study, we address these issues in a sample of behavioral variant fronto-temporal dementia patients. Furthermore, we investigate more basic face processing skills, i.e. the ability to detect a facial shape, which presumably precedes processing of identity and semantic associations according to influential face processing models (e.g. Haxby and Gobbini, 2011), as well as recognition of familiarity. We included the latter as our clinical observations supplemented with tailored neuropsychological investigations have indicated a specific degradation of familiarity processing in neurodegenerative disorders (Van den Stock et al., 2012a, Van den Stock et al., 2013).

Based on the documented involvement of the anterior temporal lobes in famous face recognition, in combination with the atrophic topography in behavioral variant fronto-temporal dementia, we expect a deficit in famous face recognition. On the other hand, considering the association between unfamiliar face recognition and face shape processing with more posterior temporal regions, we do not anticipate respective severe deficits.

## 2 Methods

### 2.1 Participants

All subjects were right-handed as assessed by the Edinburgh Handedness Inventory (Oldfield, 1971). A total of 29 consecutive behavioral variant fronto-temporal dementia patients were recruited. Six of these patients could not be included in the study since no experimental data could be acquired due to a lack of cooperation and/or agitation. The remaining 23 were recruited via the Memory Clinic (*N* = 7) and Old Age Psychiatry Department of University Hospitals Leuven (*N* = 10) and the Neurology Department of Onze-Lieve-Vrouwziekenhuis Aalst-Asse-Ninove (*N* = 6). All patients were evaluated via clinical assessment, neuropsychological testing and structural MRI. In addition, [^18^F]-Fluorodeoxyglucose Positron Emission Tomography (FDG-PET) was performed in all but three patients. Two patients fulfilled the revised diagnostic criteria of ‘behavioural variant FTD with definite FTLD Pathology’, based on a C9orf72 pathogenic mutation, and 18 patients fulfilled the criteria for ‘Probable behavioral variant fronto-temporal dementia’ (Rascovsky et al., 2011). The remaining three patients were diagnosed as ‘Possible behavioral variant fronto-temporal dementia’ (Rascovsky et al., 2011). In none of the patients, language difficulty was the most prominent clinical feature. Furthermore, in none of the patients, aphasia was the most prominent deficit at symptom onset and during the initial phase of the disease. These phenotypes do not comply with the current diagnostic criteria for primary progressive aphasia (Gorno-Tempini et al., 2011). Patients were included after clinical judgment deemed them able to successfully undergo the experimental procedure.

The control group was recruited through advertisements in local newspapers. Twenty control participants took part in the behavioral experiments and underwent structural MRI and neuropsychological assessment. The exclusion criteria consisted of present or past neurological or psychiatric disorders. This included substance abuse as well as significant systemic comorbidities or use of medication susceptible to affect the central nervous system. Demographic data and neuropsychological test results of all participants are presented in Table 1. The individual demographic and neuropsychological data of the patients, including a detailed overview of the diagnostic criteria they fulfilled, are presented in Supplementary Table S1.

### 2.2 Experiment 1: face shape detection

Materials consisted of visual images that were validated regarding face-semblance, based on a computerized face-detection algorithm as well as on subjective ratings of face-semblance (Meng et al., 2012). The dataset consists of 5 categories of images showing increasing facial shape cues. A total of 40 images was selected, 20 images from the category with the highest face-semblance and 5 images of each of the 4 remaining categories.

**Fig. 1:**
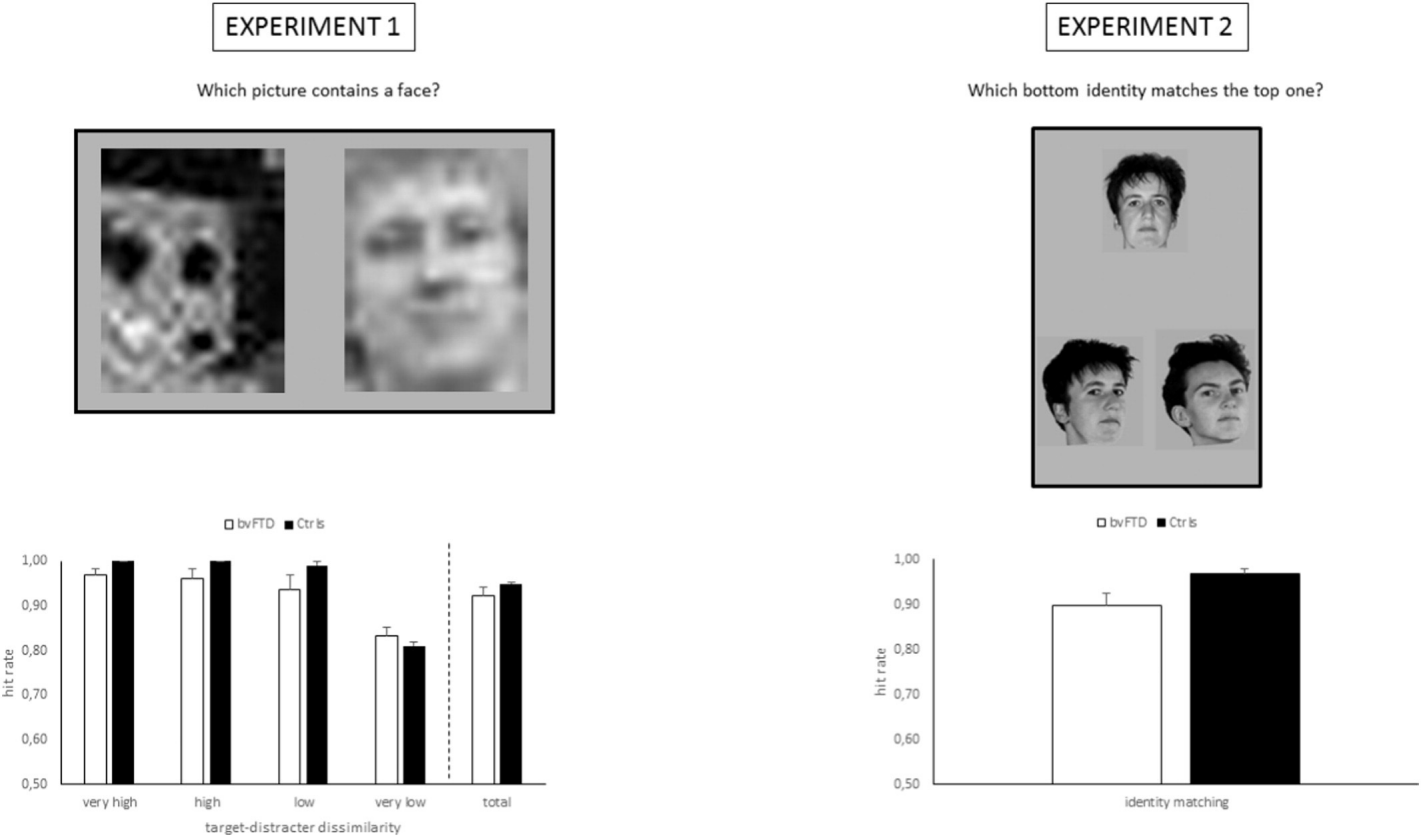
Stimulus examples (top row) and results (bottom row) of Experiment 1 (left column) and Experiment 2 (right column).

The procedure differed from the one described by Meng et al. (2012). A trial consisted of simultaneous presentation of 2 images next to each other. One of the images always was from the category with the highest face-semblance, and the second image was from one of the 4 remaining categories. This resulted in 4 conditions of increasing similarity between both stimuli regarding face shape content. Subjects were instructed to indicate by a button press which of the 2 images showed the highest face-semblance. The experiment consisted of 20 trials. Stimulus order and positions were counterbalanced. Viewing time was unlimited. See Fig. 1 for a stimulus example.

### 2.3 Experiment 2: unfamiliar face identity matching

The stimuli and procedure have been described in detail elsewhere (Van den Stock et al., 2008). In short, a stimulus consisted of a picture displaying a front view of a face presented on top, with 2 pictures displaying 3/4 views of a face presented below. One of the bottom faces showed the same identity as the one on top. Participants were instructed to indicate by a button press which identity of the two bottom pictures matched the one on top. The experiment consisted of 32 trials. Viewing time was unlimited. See Fig. 1 for a stimulus example. The face pictures were not confined to the inner part and included hairstyles in order to maintain a naturalistic picture.

### 2.4 Experiment 3: familiarity categorization and famous face-name matching

Photographs of 43 faces (26 male) of celebrities were downloaded from the internet. The identities were selected for a population aged over 50, based on their fame three to five decades ago. Additionally, 20 photographs of non-famous faces were selected. The photographs were selected and edited to display as few distinctive non-face features (e.g. hat, glasses) as possible. We selected the famous identities from a range of domains (e.g. sports, music, cinema, and politics). Another criterion for the selection of the famous identities was that the person was renowned for at least 10 years. Additionally, we selected for every celebrity two distractor names of other celebrities (not included in the picture-set) from the same gender, race and age range.

**Fig. 2:**
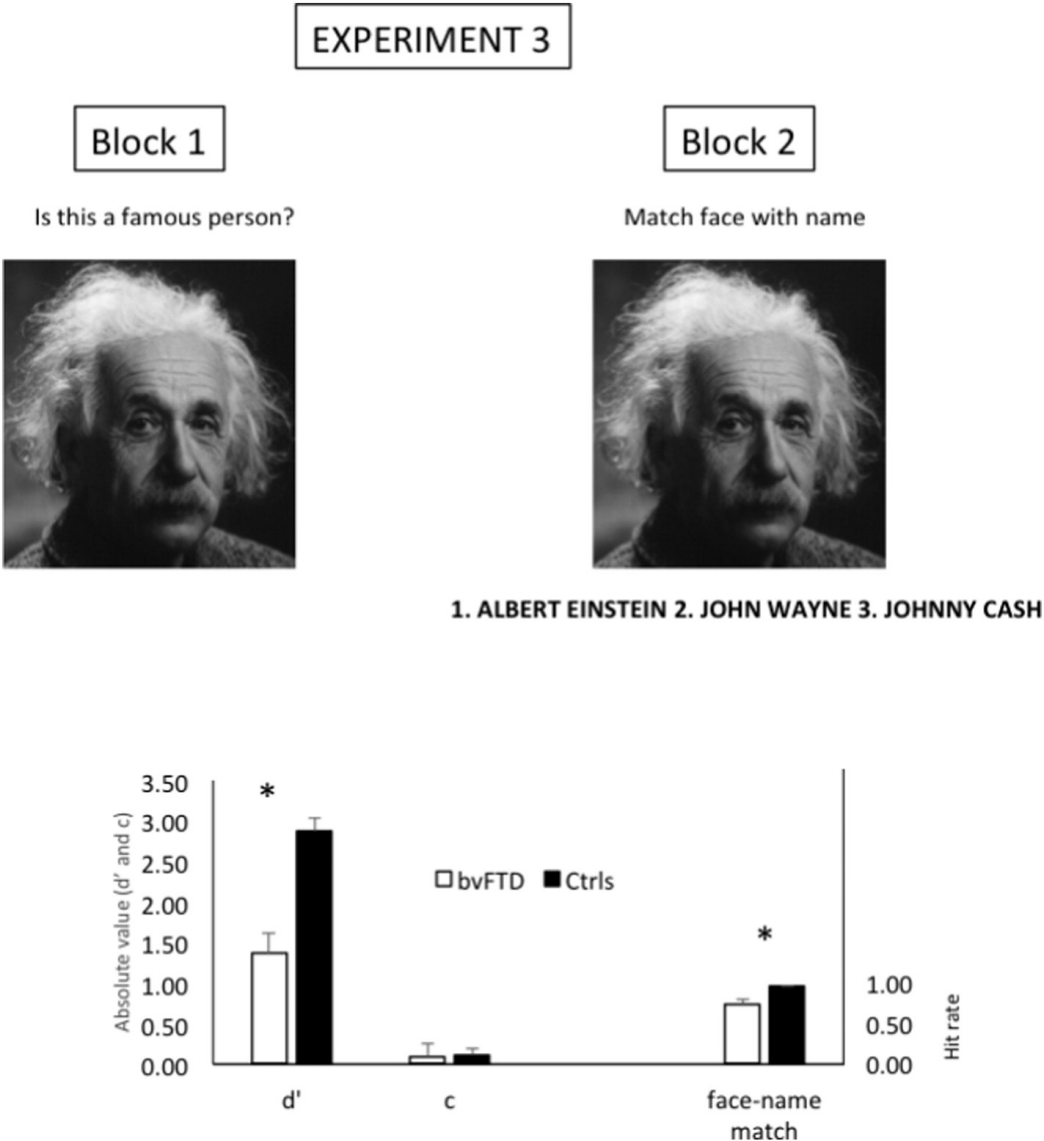
Stimulus examples (top row) and results (bottom row) of block 1 (familiarity recognition; left column) and block 2 (face-name matching; right column) of Experiment 3. *:*p* < 0.001; Face-name match is expressed in hit rate (i.e. minimum = 0 and maximum = 1). d′ = dprime; c = criterion according to signal detection theory.

Stimulus selection was based on a pilot study in which 39 healthy community dwelling elderly (19 men) (Mean (SD) age = 64.6 (5.1); age range = 60–83) participated. The pilot study mimicked the actual experiment and consisted of 2 blocks. In the first block all faces were randomly presented one by one. Participants were instructed to indicate whether the presented face was famous or not. There was no response time limit. The results showed that all faces were accurately recognized by more than half of the subjects, except for 1 famous face that was only recognized as familiar by 10 subjects (26%). This latter stimulus was not selected for the Experiment. The average familiarity recognition of all remaining faces was high (90%, STD = 8.0). In the second block, the famous faces were presented randomly one by one, with three names printed underneath (see Fig. 2). One of the names corresponded to the identity of the face above and participants performed a three alternative forced-choice face-name matching task. The results showed that all remaining famous faces were accurately matched with their written identity by at least 26 subjects (67%), with a high overall matching performance (96%, STD = 5.2).

The remaining 62 faces (42 famous, 35 male) were selected for Experiment 3, which followed the same procedure as the pilot study and is purely visual in nature. The full stimulus set is provided in the supplementary materials.

### 2.5 Imaging

Scanning of all subjects was performed on a single 3T Philips Achieva system equipped with a 32-channel head coil. A high-resolution T1-weighted anatomical image (voxel size = 0.98 × 0.98 × 1.20 mm^3^) was acquired using a 3D turbo field echo sequence (TR = 9.6 ms; TE = 4.6 ms; matrix size = 256 × 256; 182 slices). Analysis of local grey matter (GM) volume was performed with SPM8 (Wellcome Trust Centre for Neuroimaging, UCL, London, United Kingdom) within MatLab R2008a (Mathworks, Natick, MA). Preprocessing included image segmentation, spatial normalization, modulation and smoothing. Segmentation was performed using SPM8's unified segmentation routine in combination with in-house developed algorithms to address suboptimal segmentation results in the most atrophic regions, primarily the right temporal pole. Next, the images were spatially normalized by creating a customized group-specific template using SPM8's DARTEL routine and warping each of the individual GM segmentations onto this template. The warped GM segmentations were modulated to account for local shape differences and smoothed using a Gaussian kernel of 8 mm at FWHM. To investigate regional group differences in grey matter volume, we performed a two samples *t*-test on the grey matter voxels (*p*_height_ < 0.005, minimal cluster size k_E_ = 100 voxels).

The GM maps were subsequently used in a regression analysis in which behavioral performance was entered as covariate in order to investigate correlations between performance and voxel-wise GM volume (*p*_heigh_ < 0.005, minimal cluster size k_E_ = 100 voxels). As the primary focus of the present study was to gain insight into face recognition in behavioral variant fronto-temporal dementia and its associated structural neuro-anatomy, rather than into face recognition per se, we opted to confine the regression analysis to the patient group and hence not to combine it with the data from the control group. Although this does not benefit statistical power, it excludes contamination of the results by non-behavioral variant fronto-temporal dementia data. While the alternative approach has proven valuable (Kumfor et al., 2013, Kumfor et al., 2014b), the current method provides complementary evidence to it as well as to region of interest analyses (Bertoux et al., 2012b, Couto et al., 2013). Furthermore, we did not a priori include demographic or cognitive disease-related confounding variables in the regression analysis, in line with previous volumetric studies in neurodegenerative syndromes (Bertoux et al., 2012b, Cerami et al., 2014, Couto et al., 2013, Eslinger et al., 2007, Gefen et al., 2013, Kipps et al., 2009, Kumfor et al., 2014a, Werner et al., 2007). However, we correlated age and MMSE-score with the behavioral variables of interest and included them as confounding variable in case the correlation was significant.

## 3 Results

### 3.1 Behavior

Trials in which the reaction time differed more than three standard deviations from the subject-specific mean reaction time were defined as outliers. These trials were excluded from all further analyses. All subsequent analyses are performed on accuracy data. To test for normality of the data, Shapiro-Wilk tests were performed on the relevant variables. In cases where a normal distribution could not be assumed, we performed non-parametric Independent-Samples Mann-Whitney *U* tests to investigate group differences. If a normal distribution could be assumed, we performed Independent-Samples *t*-tests to investigate group differences. For the latter, Levene's tests was used to test homoscedasticity. If the null hypothesis of equal variances was rejected, Welch's *t*-test was used (an adaptation of Student's *t*-test which accounts for unequal variances).

#### 3.1.1 Experiment 1

Two patients did not participate in Experiment 1. A total of 25 outlier trials were identified (out of 820 = 3.0%; maximum/participant = 1). The results are displayed in Fig. 1. The controls showed a ceiling effect on the 3 conditions in which the distracter images showed the lowest face semblance (only 1 trial on a total of 300 was incorrect). Therefore, we investigated the significance of the group difference on the condition in which the difference in face semblance between both images was minimal, i.e. the condition with the highest task difficulty (only 1 control subject performed flawless on this condition) and the total score. An Independent-Samples Mann-Whitney *U* test did not reveal a significant group difference for the high difficulty condition (*p* = 0.092), nor for the total score (*p* = 0.74). To aid the interpretation of the results, i.e. to examine whether better performance for the very low dissimilarity condition is balanced out by worse performance for other conditions, we subsequently compared the remaining 3 conditions. The results did not reveal a significant group difference for any of the conditions (*p* ≥ 0.17). Notably, the behavioral variant fronto-temporal dementia group outperformed the control group on the high difficulty condition, although this difference was not significant.

#### 3.1.2 Experiment 2

Two patients did not participate in Experiment 2. A total of 24 outlier trials were identified (out of 1312 = 1.8%; maximum/participant = 1). The results are displayed in Fig. 1. Independent-Samples Mann-Whitney *U* tests on the total score did not reveal a significant group difference (*p* = 0.119).

#### 3.1.3 Experiment 3

Two patients did not participate in Experiment 3. The results are displayed in Fig. 2.

##### 3.1.3.1 Familiarity sensitivity

The results from the first block (familiarity categorization) were analyzed according to signal detection theory. Signal detection analysis allows to calculate a sensitivity index d′ which accounts for response bias. We calculated d′ (= Z(hit rate) − Z(false alarm rate)) as an index of familiarity detection sensitivity (Stanislaw and Todorov, 1999). One control subject obtained a maximal hit rate and 13 subjects (4 behavioral variant fronto-temporal dementia) obtained a minimal false alarm rate. These extreme values were transposed to 1 − 1 / (2 ∗ N_(famous faces)_) and 1 / (2 ∗ N_(unfamiliar faces)_ respectively, i.e. 1 − 1/(2 ∗ 42) = 0.988 and 1 / (2 ∗ 20) = 0.025. An Independent-Samples Mann-Whitney *U* test on d′ revealed a significant group difference (*p* < 0.001). To investigate whether the impaired familiarity recognition was driven by a difference in response bias, we calculated criterion (c) (= −[(Z(hit rate + Z(false alarm rate) / 2]). A negative value of c reflects a liberal response bias, whereas a positive c reflects a conservative response bias. An Independent-Samples Mann-Whitney *U* tests revealed no significant group difference for the value of c (*p* = 0.584).

##### 3.1.3.2 Face-name matching sensitivity

Secondly, we compared performance between groups on the second block (famous face-name matching). A Mann-Whitney *U* test on the proportion correct responses revealed a significant group difference (*p* < 0.001). The results are displayed in Fig. 2.

### 3.2 Imaging

#### 3.2.1 VBM group comparison

**Fig. 3:**
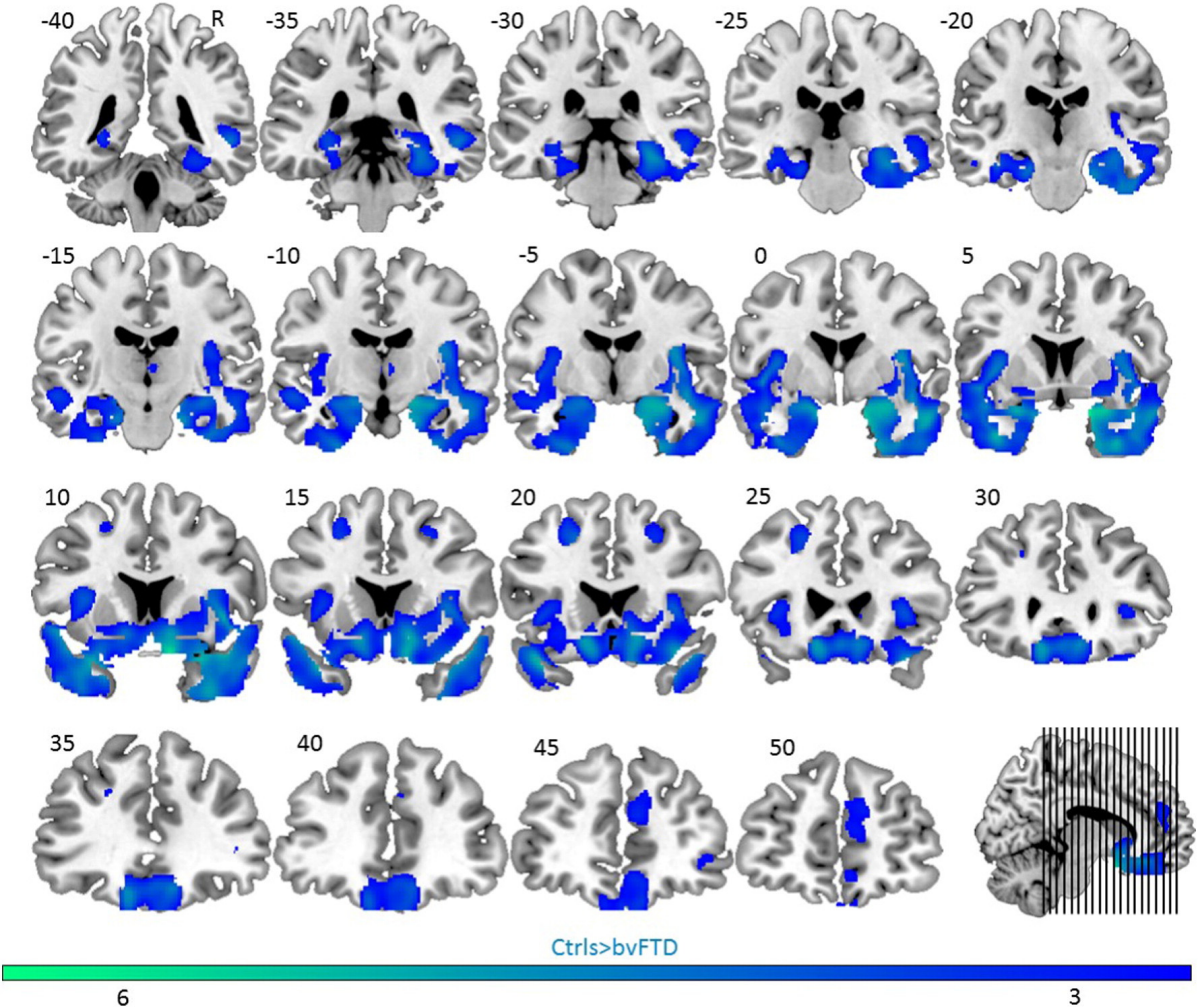
Atrophic topography of patient group. Statistical map (*p* < 0.005, minimal cluster size = 100 voxels) of group differences in grey matter volume, represented on coronal slices from posterior (top left) to anterior (bottom right) (Controls > bvFTD). Numbers refer to MNI Y-coordinates. Color coding refers to t-values.

The imaging results of four patients could not be included due to excessive motion in the scanner. A two samples *t*-test (*p*_height_ < 0.005, minimal clustersize k_E_ = 100 voxels) revealed a large bilateral cluster covering the anterior half of the temporal lobes, insula, ventral striatum and orbitofrontal cortex. In addition, the bilateral dorsolateral prefrontal cortex and a cluster in the medial prefrontal cortex was atrophic, consistent with previous studies (Seeley et al., 2008, Whitwell et al., 2009) (Fig. 3).

#### 3.2.2 VBM multiple regression

VBM regression analyses were performed on the behavioral variables that showed significant group differences, i.e. performance on Experiment 3. These were entered as predictors in a regression analysis with GM volume.

##### 3.2.2.1 Familiarity sensitivity

**Fig. 4:**
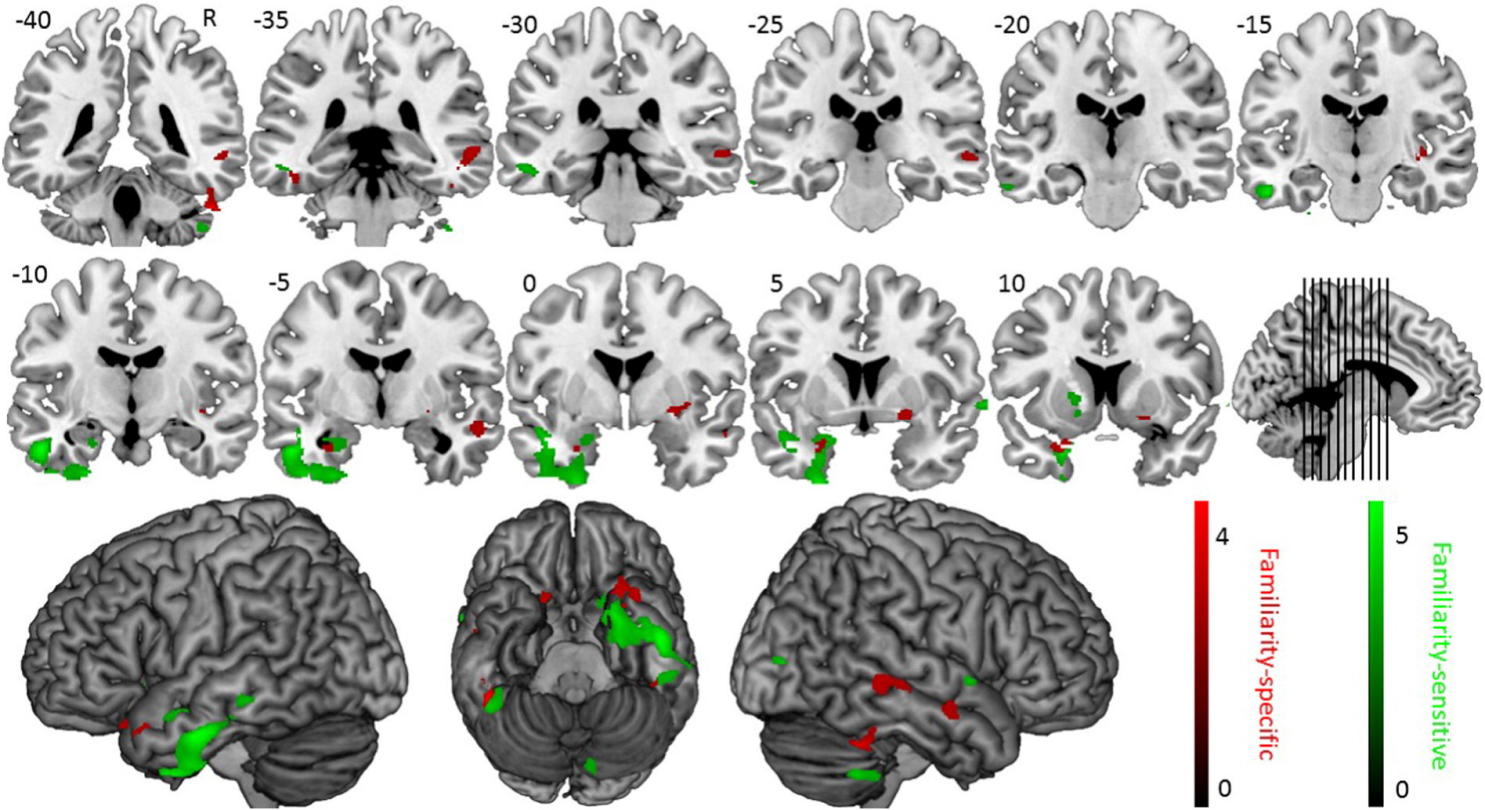
Multiple regression results for familiarity-sensitivity (i.e. positive correlation with d′ of block 1 of Experiment 3) and familiarity-specificity (i.e. negative correlation with the number of celebrities for which the familiarity was not accurately recognized in block 1 of Experiment 3 and for which the name was accurately matched with the face in block 2 of Experiment 3).

We first investigated familiarity-sensitive associations with GM volume. For this purpose, we performed a regression analysis with d′ as single predictor of interest (*p* < 0.005, minimal cluster size k_E_ = 100 voxels). As age (ρ(21) = − 0.438, *p* = 0.047), but not MMSE-score (ρ(21) = − 0.437, *p* = 0.054) correlated with d′, we included age as a confounding variable in the regression analysis. The results are displayed in Fig. 4 and Table 2.

##### 3.2.2.2 Face-name matching sensitivity

**Fig. 5:**
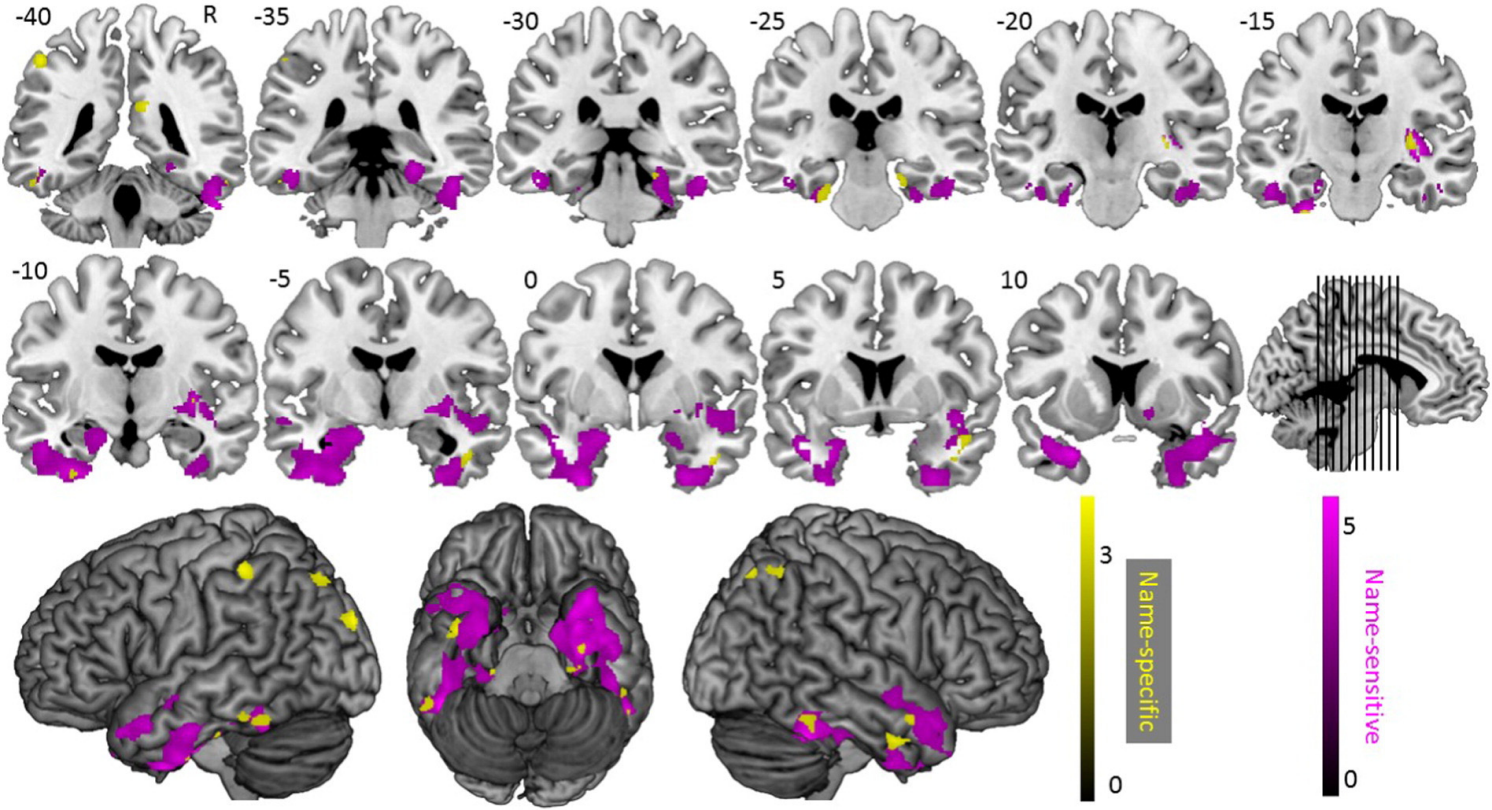
Multiple regression results for face-name matching-sensitivity (i.e. positive correlation with proportion correct responses in block 2 of Experiment 3) and face-name matching-specificity (i.e. negative correlation with the number of celebrities for which the familiarity was accurately recognized in block 1 of Experiment 3 and for which the name was not accurately matched with the face in block 2 of Experiment 3).

Subsequently, we investigated face-name matching-sensitive associations with GM volume by performing a regression analysis with face-name matching score as single predictor (*p* < 0.005, minimal cluster size k_E_ = 100 voxels). Neither age (ρ(21) = − 0.337, *p* = 0.14) nor MMSE-score (ρ(20) = 0.415, *p* = 0.069) showed a significant correlation with the predictor of interest and were therefore not included as confounding variables. The results are displayed in Fig. 5 and Table 2.

##### 3.2.2.3 Familiarity specificity

Secondly, we investigated familiarity-specific deficit associations with GM volume. For this purpose, we computed the number of celebrities for which the familiarity was not accurately recognized (in block 1), but the name was (in block 2).

##### 3.2.2.4 Face-name matching specificity

Similarly, to investigate the structural neuro-anatomy specifically associated with face-name matching deficit in behavioral variant fronto-temporal dementia, we computed the number of celebrities for which the familiarity was accurately recognized (in block 1), but not the name (in block 2). Age did not correlate significantly with any of both predictors of interest (ρ(20) ≤ │0.352│, *p* ≥ 0.118). MMSE-score correlated significantly with the number of name-specific errors (ρ(20) = − 0.541, *p* = 0.014), but not with the number of familiarity-specific errors (ρ(20) = 0.074, *p* = 0.76). Hence, MMSE-score was included as a confounding predictor in the regression analysis with number of name-specific errors. The results are displayed in Fig. 4, Fig. 5 and Table 2. Behaviorally, there was no significant correlation between the number of familiarity-specific and name-specific errors (ρ(21) = − 0.198, *p* = 0.39).

### 3.3 Correlation of name matching indices with language assessment

Finally, we computed Spearman correlations between the three variables involving performance on block 2 of Experiment 3 (i.e. total face-name matching score, familiarity specific index and face-name matching specific index) on the one hand and performance on the three language tests included in the neuropsychological test battery (i.e. Animal Verbal Fluency (AVF), Boston Naming Test (BNT) and the comprehension subtest from the Aachen Aphasia Test (AAT_comp)) on the other hand. This revealed a significant correlation between the total score on face-name matching and Boston Naming Test (ρ(20) = 0.522, *p* = 0.021), but not between any other combination (lowest *p* = 0.16).

## 4 Discussion

The aim of the present study was to investigate recognition of face shape and identity in behavioral variant fronto-temporal dementia and more specifically how any deficits relate to familiarity recognition and fame of the face. Although several studies have addressed face emotion processing in behavioral variant fronto-temporal dementia, there is only limited evidence on how familiarity and identity processing are affected. For this purpose, we recruited a group of behavioral variant fronto-temporal dementia patients with only mild general cognitive decline, as evidenced by an average MMSE above 26 and displaying an atrophic pattern in anterior temporal, orbitofrontal, medial prefrontal and insular regions, typically associated with early behavioral variant fronto-temporal dementia (Seeley et al., 2008). The study investigated both perceptual and semantic face processing. The first experiment assessed detection of facial shape in noisy images (Meng et al., 2012) and the second experiment primarily tapped into recognizing unfamiliar face identities from different viewpoints. Both these tasks are predominantly perceptual in nature, while the third experiment made use of semantically unique items to assess semantic and affective face associations like familiarity and identification of famous faces. Several tests have been developed assessing recognition of famous people (e.g. Albert et al., 1980, Albert et al., 1979, Gefen et al., 2013, Hamsher and Roberts, 1985, Hodges et al., 1993), but many are outdated and all of them are by definition culturally dependent. The validity of the famous face assessment we performed in Experiment 3 was established by means of a pilot study in which demographically matched control group rated the stimuli. We considered this essential as recognition of famous faces is highly dependent on factors like age and geographic location. In addition, we included a familiarity categorization task as familiarity processing has been proposed as a key mechanism in person identification and associated deficits (Bartolomeo et al., 1998, Barton, 2003, Barton, 2008, Barton et al., 2006, Hirstein and Ramachandran, 1997). Furthermore, a 3 alternative forced-choice face-name matching task was employed rather than a free naming task, as lexical retrieval is a dominant cognitive process in the latter task, while the purpose of the study was to examine the recognition of semantic associations of faces. Furthermore, face identification based on lexical retrieval (as opposed to lexical recognition) is a frequent subjective complaint of healthy elderly (Bolla et al., 1991).

The main result is that behavioral variant fronto-temporal dementia patients perform equal to controls in perceptual tasks but not in familiarity or name-matching tasks. The neuropsychological pattern in the behavioral variant fronto-temporal dementia group is compatible with the profile of associative prosopagnosia (De Renzi et al., 1991), consisting of relatively intact perceptual coding of faces (in this case evidenced by intact face shape detection and intact unfamiliar face identity matching), in combination with impaired recognition of associative facial attributes (in this case evidenced by a deficit in familiarity recognition and face-name matching) (de Gelder and Van den Stock, 2015). While a (selective) deficit in famous face recognition has been reported in language (Gefen et al., 2013, Snowden et al., 2004, Snowden et al., 2012) and temporal (Gorno-Tempini et al., 2004, Omar et al., 2011b) variants of fronto-temporal lobar degeneration, to our knowledge there have hitherto not been any reports on famous face recognition deficits in behavioral variant fronto-temporal dementia.

Secondly, the deficits in familiarity and name recognition correlate with temporal volume loss. We first investigated the areas that were volumetrically associated with a measure of familiarity recognition that controlled for response tendencies, i.e. d′ (Stanislaw and Todorov, 1999). The results revealed a set of clusters that have previously been associated with deficits in face familiarity recognition, i.e. a large cluster in the left ventral ATL (Gefen et al., 2013), right temporal pole (Gefen et al., 2013) and cerebellar vermis (Van den Stock et al., 2012b). Face-name matching was primarily associated with grey matter volume of the bilateral ATLs, as reported previously in fronto-temporal lobar degeneration language variants (Gefen et al., 2013). In addition, we investigated the structural neuro-anatomy associated with familiarity-specific and name-specific deficits in famous face recognition. The results for familiarity-specific processing again included the left temporal pole (Gefen et al., 2013) and cerebellar vermis (Van den Stock et al., 2012b), but also clusters in the left hippocampus, right superior temporal sulcus (STS) and middle temporal gyrus (MTG), which have also been associated with face familiarity recognition (Smith et al., 2014, Von Der Heide et al., 2013). Name-specific processing was again associated with grey matter volume of regions in the bilateral anterior temporal cortices, as well as parietal areas. The latter have also been associated with covert lexical retrieval of famous face names (Gesierich et al., 2012, Gorno-Tempini et al., 1998).

The imaging results are in line with clinical reports that document the involvement of the ATLs in famous face recognition deficits (Gefen et al., 2013, Snowden et al., 2004, Snowden et al., 2012). It has been hypothesized that the left ATL is primarily involved in lexical-semantic processing, while the right ATL is mainly associated with non-verbal representations (Gainotti, 2015). The present results are in line with this and additionally suggest that familiarity recognition deficits in behavioral variant fronto-temporal dementia are also associated with left anterior temporal atrophy in addition to right middle and anterior STS, while name recognition deficits show a bilateral association with structural integrity of anterior temporal cortices. These results extend the observations of Gefen et al. (2013), who reported a left lateralization for naming famous faces and a bilateral association for recognizing famous faces in primary progressive aphasia. Our findings provide two nuances to these results: first, the involvement of the left ATL in famous face recognition deficits extends beyond free naming and includes familiarity detection; and second, atrophy of the right ATL is involved in name recognition deficits in fronto-temporal lobar degeneration.

These results, in combination with the correlation with confrontation naming ability, support the notion of the ATL as a semantic hub. While familiarity-sensitive results do not exclude semantic processing, familiarity-specific results constitutes pre-semantic processing. The present results document the involvement of more posterior temporal areas in familiarity-specific processes.

In addition to the ATLs, the imaging results include areas (also outside the atrophic clusters) that are functionally connected to the ATLs and show reduced (task free) connectivity in semantic variant primary progressive aphasia (Guo et al., 2013). In line with this, functional changes during semantic tasks have been reported in semantic variant primary progressive aphasia in distant regions from the ATL, like the posterior inferior temporal gyrus and temporo-parietal junction (Mummery et al., 1999). The volumetric association of distant regions of ATL with performance during famous face recognition is compatible with the proposed semantic hub-function of the ATL (Guo et al., 2013), where distant areas may partly compensate for neurodegenerative damage to the ATL.

Some negative results and limitations of the present study should be noted. Although the experimental set-up was designed with increasing complexity along a perceptual-semantic dimension regarding face processing, the procedure did not include a name-comprehension task. A purely verbal comprehension task (e.g. a proper name task without faces) would assess mainly semantic processing and constitute a more extreme position along the perceptual-semantic dimension. However, as the aim of the study was to investigate face-processing, an experimental verbal comprehension task was not included in the design. Instead we related the face-name matching results to performance on conventional language tests included in the neuropsychological test battery. The results provide indirect support that the face-name matching performance reflected face identification abilities (i.e. the significant correlation with confrontation naming on Boston Naming Test), rather than proper name comprehension (i.e. no significant correlation with language comprehension on AAT_comp).

The intact identity processing we observed here contrasts with recent evidence for impaired identity processing in behavioral variant fronto-temporal dementia (Kumfor et al., 2015). This discrepancy might be explained by two factors. First, Kumfor et al. (2015) made use of an identity discrimination task with facial stimuli containing only the inner face, i.e. with identifying features like hair and ears removed. Secondly, the identity processing task in Kumfor et al. (2015) consisted of emotional mixed with neutral stimuli. Although the emotional information was task irrelevant, there is accumulating evidence that task irrelevant facial emotion influences facial identity recognition (Chen et al., 2011, Gallegos and Tranel, 2005, Kaufmann and Schweinberger, 2004, Levy and Bentin, 2008, Van den Stock and de Gelder, 2012, Van den Stock and de Gelder, 2014, Van den Stock et al., 2008). These differences may account for the discrepancy with the present results showing intact matching of neutral whole face identities.

We did not include any control conditions for the face conditions and have hence no indication of the face-specificity of the results. Famous buildings have typically been used as control category for famous faces and the results show primarily common, but also distinct neural and behavioral effects (Gorno-Tempini and Price, 2001, Van den Stock and de Gelder, 2012).

Finally, as we did not include a clinical control group, we have no indications of the syndrome-specific characteristics of the results. It remains to be shown that neural damage with a similar topography but with different symptomatology, results in comparable behavioral profiles. For instance, patients with right-lateralized semantic variant primary progressive aphasia and patients with behavioral variant fronto-temporal dementia show overlap in atrophic topography, but distinct clinical and neuropsychological profiles (Kamminga et al., 2014).

In conclusion, the results point to a deficit in familiarity and name recognition of famous faces in behavioral variant fronto-temporal dementia, in combination with intact unfamiliar face shape and identity recognition. Familiarity recognition was primarily associated with left ATL atrophy, while name recognition was predominantly associated with bilateral ATL atrophy. These findings extent previous results by documenting the involvement of the left ATL in familiarity detection and the right ATL in name recognition deficits in fronto-temporal lobar degeneration.

The following are the supplementary data related to this article.

Table S1 . Case summaries of demographic and behavioral data. YDD = disease duration based on heteroanamnesis; MMSE = Mini-Mental State Examination; A1-A5 = sum of scores on trials A1 to A5 of the RAVLT (Rey’s Auditory Verbal Learning Test); %Recall = [score on trial A7 (delayed recall) / (maximum of trials A1 to A5) of the RAVLT]*100; Recog = correct hits − false hits on trial A8 (recognition) of the RAVLT; TMT = Trail Making Test; AVF = Animal Verbal Fluency (1 minute); RCPMT = Raven's Colored Progressive Matrices Test (sets A & B); Compr = Score on Comprehension subtest of the Aachen Aphasia Test; BNT = Boston Naming Test; BORB = Birmingham Object Recognition Battery; Le = Length matching; Si = Size matching; Or = Orientation matching; DiagnCrit_A = Diagnostic Criterium (Rascovsky et al., 2011); A = Early behavioral disinhibition; B = Early apathy or inertia; C = Early loss of sympathy or empathy; D = Early perseverative, stereotyped or compulsive/ritualistic behavior; E = Hyperorality and dietary changes; F = Neuropsychological profile: executive/generation deficits with relative sparing of memory and visuospatial functions. Table S1

Suppl. S1 Stimuli of Experiment 3 - block 1 (familiarity detection). Suppl. S1

Suppl. S2 Stimuli of Experiment 3 - block 2 (face-name matching). Suppl. S2

## Figures and Tables

**Table 1:** Demographic and neuropsychological test results. MMSE = Mini-Mental-State Examination; RAVLT = Rey Auditory Verbal Learning Test; A1–A5 = the sum of scores on trials A1 to A5 of the RAVLT; Recognition = the recognition score constitutes the difference between the number of correct hits and false hits on the recognition trial; BNT = Boston Naming Test; AVF = Animal Verbal Fluency; TMT = Trail Making Test; BORB =  Birmingham Object Recognition Battery; RCPMT = Raven Colored Progressive Matrices Test; AAT = Aachen Aphasia Test. ^£^ = (*N* = 21); ^%^ = (*N* = 20); ^$^ = (*N* = 19); ^§^ = (*N* = 17); ^&^ = (*N* = 15).

		bvFTD (*N* = 23)	Controls (*N* = 20)		
		t (χ^2^)	p
Age (SD)		64.5 (9.8)	66.6 (6.1)	0.854	0.398
Sex (M/F)		13/10	12/8	(0.000)	1.000
MMSE		26.7 (1.5)^£^	29.2 (0.6)	7.124	0.001
RAVLT	A1–A5	29.0 (11.3)^%^	50.8 (7.3)	7.262	0.001
% recall	56.1 (31.9)^%^	80.9 (17.4)	3.060	0.005
Recognition	6.5 (7.5)^%^	14.0 (1.3)	2.135	0.043
BNT		40.3 (12.7)^%^	54.4 (2.9)	4.861	0.001
AVF		15.0 (5.5)^%^	22.1 (5.8)	4.016	0.001
TMT	A (secs)	63.5 (42.7)^$^	32.5 (9.4)	3.099	0.006
B (secs)	193.1 (141.2)^&^	89.8 (42.3)	2.742	0.015
BORB	Length	87.6 (7.3)^§^	90.1 (4.5)	1.262	0.218
Size	85.5 (6.9)^§^	88.9 (6.3)	1.569	0.126
Orientation	81.4 (9.2)^§^	86.1 (6.0)	1.845	0.074
RCPMT		16.4 (3.9)^%^	20.8 (2.8)	4.214	0.001
AAT	Comprehension	93. 9 (12.3)^$^	109.5 (5.3)	5.093	0.001

**Table 2:** Imaging results. ITS: inferior temporal sulcus; TP_mid: middle temporal pole; TP_sup: superior temporal pole; MOG: middle occipital gyrus; FG: fusiform gyrus; PHC: parahippocampal cortex; ITG: inferior temporal gyrus; IFG_orb: inferior frontal gyrus, pars orbitalis; MTG: middle temporal gyrus; STS: superior temporal sulcus; SOG: superior occipital gyrus; IPL: inferior parietal lobule; ACC: anterior cingulate cortex; PCC: posterior cingulate cortex; SPL: superior parietal lobule; AG: angular gyrus. XYZ refer to MNI-coordinates.

Predictor		N	T	p	X	Y	Z	BA
*d′ (familiarity-sensitive; confound variable: age)*								
ITS	L	8599	5,06	0,000074	− 50	− 7	− 28	20
TP_mid	L		4,57	0,00019	− 24	6	− 46	36
TP_sup	L		4,26	0,00034	− 23	10	− 30	28
TP_sup	R	218	3,95	0,00064	69	6	− 2	38
ITS	L	445	3,64	0,0012	− 55	− 30	− 12	20
MOG	R	106	3,98	0,00061	32	− 81	6	18
Putamen	L	1878	4,49	0,00022	− 22	19	− 1	
Putamen	L		3,39	0,0020	− 20	17	− 10	
Caudate nucleus	L		3,17	0,0031	− 11	19	− 2	
Cerebellum crus 2	R	658	3,96	0,00062	48	− 47	− 45	
Cerebellum crus 2	L	262	3,30	0,0024	− 9	− 81	− 26	

*Face-name matching score (name-sensitive)*								
FG	L	17112	5,23	0,000043	− 28	− 16	− 38	20
TP_sup	L		4,83	0,000092	− 34	16	− 28	38
TP_sup	L		4,62	0,00014	− 27	9	− 30	28
PHC	R	353	3,12	0,0033	20	2	− 20	34
Cerebellum, crus 1/ITG	R	20259	5,62	0,000021	52	− 43	− 33	20
Insula	R		5,45	0,000034	33	− 18	1	48
Pallidum	R		4,16	0,00037	25	− 5	− 5	
Putamen	R	133	3,25	0,0025	15	9	− 7	

*Familiarity-specific errors (familiarity-specific)*								
ITG/cerebellum, crus 1	R	534	4,79	0,00010	50	− 43	− 33	20
ITG	R		3,02	0,0041	47	− 37	− 23	20
IFG_orb	L	1709	3,76	0,00086	− 25	19	− 23	38
TP_sup	L		3,69	0,00099	− 36	18	− 26	38
TP_sup	L		3,60	0,0012	− 24	9	− 26	28
MTG	R	1046	3,46	0,0016	55	− 38	− 6	21
MTG	R		3,23	0,0026	60	− 27	− 4	21
STS	R	379	3,27	0,0024	54	− 4	− 16	21
ITG	L	109	3,15	0,0030	− 45	− 35	− 17	20
olfactory cortex	R	207	3,55	0,0013	16	15	− 26	11
Insula	R	210	3,25	0,0025	37	− 16	− 5	48
Pallidum	R	637	3,43	0,0017	25	− 0	− 6	
Gyrus rectus	R		3,24	0,0026	20	17	− 12	
Putamen	R		2,99	0,0043	34	− 1	− 2	
Hippocampus	L	155	3,07	0,0036	− 30	− 4	− 26	

*Name matching-specific errors (name-specific; confound variable: MMSE)*								
SOG	L	551	4,04	0,00054	− 25	− 93	27	18
IPL	L	380	3,89	0,00073	− 51	− 38	52	40
ITS	R	296	3,76	0,00095	43	− 2	− 31	20
ACC	L	149	3,75	0,00097	− 3	47	6	10
PCC	R	177	3,63	0,0012	11	− 39	24	26
FG	L	104	3,59	0,0013	− 30	− 12	− 40	20
SPL	R	231	3,58	0,0014	36	− 72	51	7
PHC	R	196	3,49	0,0016	20	− 27	− 18	30
STS	R	197	3,40	0,0020	47	6	− 22	21
IPL	L	362	3,37	0,0021	− 31	− 76	48	7
IPL	L		3,14	0,0034	− 27	− 84	45	7
AG	R	343	3,34	0,0022	35	− 57	51	7
PHC	L	233	3,25	0,0027	− 26	− 25	− 28	30
ITG	R	329	3,25	0,0027	58	− 45	− 21	20
ITG	L	469	3,23	0,0028	− 55	− 38	− 21	20
ITG	L		3,22	0,0029	− 52	− 47	− 22	20
ITS	L		3,00	0,0045	− 51	− 42	− 14	20
Putamen	R	302	3,75	0,00096	31	− 18	3	
Putamen	R		3,02	0,0043	33	− 7	− 3	

## References

[bb0005] Albert M.S., Butters N., Levin J. (1979). Temporal gradients in the retrograde amnesia of patients with alcoholic Korsakoff's disease. Arch. Neurol..

[bb0010] Albert M.S., Butters N., Brandt J. (1980). Memory for remote events in alcoholics. J. Stud. Alcohol.

[bb0015] Baez S., Manes F., Huepe D., Torralva T., Fiorentino N., Richter F., Ibanez A. (2014). Primary empathy deficits in frontotemporal dementia. Front. Aging Neurosci..

[bb0020] Bartolomeo P., Bachoud-Levi A.C., De Gelder B., Denes G., Dalla Barba G., Brugieres P., Degos J.D. (1998). Multiple-domain dissociation between impaired visual perception and preserved mental imagery in a patient with bilateral extrastriate lesions. Neuropsychologia.

[bb0025] Barton J.J. (2003). Disorders of face perception and recognition. Neurol. Clin..

[bb0030] Barton J.J. (2008). Structure and function in acquired prosopagnosia: lessons from a series of 10 patients with brain damage. J. Neuropsychol..

[bb0035] Barton J.J., Radcliffe N., Cherkasova M.V., Edelman J., Intriligator J.M. (2006). Information processing during face recognition: the effects of familiarity, inversion, and morphing on scanning fixations. Perception.

[bb0040] Bediou B., Ryff I., Mercier B., Milliery M., Henaff M.A., D'Amato T., Krolak-Salmon P. (2009). Impaired social cognition in mild Alzheimer disease. J. Geriatr. Psychiatry Neurol..

[bb0045] Bertoux M., Delavest M., de Souza L.C., Funkiewiez A., Lepine J.P., Fossati P., Sarazin M. (2012). Social cognition and emotional assessment differentiates frontotemporal dementia from depression. J. Neurol. Neurosurg. Psychiatry.

[bb0050] Bertoux M., Volle E., Funkiewiez A., de Souza L.C., Leclercq D., Dubois B. (2012). Social cognition and emotional assessment (SEA) is a marker of medial and orbital frontal functions: a voxel-based morphometry study in behavioral variant of frontotemporal degeneration. J. Int. Neuropsychol. Soc..

[bb0055] Bertoux M., de Souza L.C., Sarazin M., Funkiewiez A., Dubois B., Hornberger M. (2014). How preserved is emotion recognition in Alzheimer disease compared with behavioral variant frontotemporal dementia?. Alzheimer Dis. Assoc. Disord..

[bb0060] Bobes M.A., Lage Castellanos A., Quinones I., Garcia L., Valdes-Sosa M. (2013). Timing and tuning for familiarity of cortical responses to faces. PLoS One.

[bb0065] Bolla K.I., Lindgren K.N., Bonaccorsy C., Bleecker M.L. (1991). Memory complaints in older adults. Fact or fiction?. Arch. Neurol..

[bb0070] Burton A.M., Jenkins R., Calder A.J., Rhodes G., Johnson M.H., Haxby J.V. (2011). Unfamiliar Face Perception. The Oxford Handbook of Face Perception.

[bb0075] Cerami C., Dodich A., Canessa N., Crespi C., Marcone A., Cortese F., Cappa S.F. (2014). Neural correlates of empathic impairment in the behavioral variant of frontotemporal dementia. Alzheimers Dement..

[bb0080] Chen W., Lander K., Liu C.H. (2011). Matching faces with emotional expressions. Front. Psychol..

[bb0085] Couto B., Manes F., Montanes P., Matallana D., Reyes P., Velasquez M., Ibanez A. (2013). Structural neuroimaging of social cognition in progressive non-fluent aphasia and behavioral variant of frontotemporal dementia. Front. Hum. Neurosci..

[bb0090] de Gelder B., Van den Stock J., Wright J.D. (2015). Prosopagnosia. International Encyclopedia of the Social & Behavioral Sciences.

[bb0095] De Renzi E., Faglioni P., Grossi D., Nichelli P. (1991). Apperceptive and associative forms of prosopagnosia. Cortex.

[bb0100] Diehl-Schmid J., Pohl C., Ruprecht C., Wagenpfeil S., Foerstl H., Kurz A. (2007). The Ekman 60 Faces Test as a diagnostic instrument in frontotemporal dementia. Arch. Clin. Neuropsychol..

[bb0105] Eslinger P.J., Moore P., Troiani V., Antani S., Cross K., Kwok S., Grossman M. (2007). Oops! Resolving social dilemmas in frontotemporal dementia. J. Neurol. Neurosurg. Psychiatry.

[bb0110] Fernandez-Duque D., Black S.E. (2005). Impaired recognition of negative facial emotions in patients with frontotemporal dementia. Neuropsychologia.

[bb0115] Gainotti G. (2015). Is the difference between right and left ATLs due to the distinction between general and social cognition or between verbal and non-verbal representations?. Neurosci. Biobehav. Rev..

[bb0120] Gallegos D.R., Tranel D. (2005). Positive facial affect facilitates the identification of famous faces. Brain Lang..

[bb0125] Gefen T., Wieneke C., Martersteck A., Whitney K., Weintraub S., Mesulam M.M., Rogalski E. (2013). Naming vs knowing faces in primary progressive aphasia: a tale of 2 hemispheres. Neurology.

[bb0130] Gesierich B., Jovicich J., Riello M., Adriani M., Monti A., Brentari V., Gorno-Tempini M.L. (2012). Distinct neural substrates for semantic knowledge and naming in the temporoparietal network. Cereb. Cortex.

[bb0135] Gorno-Tempini M.L., Price C.J. (2001). Identification of famous faces and buildings: a functional neuroimaging study of semantically unique items. Brain.

[bb0140] Gorno-Tempini M.L., Price C.J., Josephs O., Vandenberghe R., Cappa S.F., Kapur N., Frackowiak R.S. (1998). The neural systems sustaining face and proper-name processing. Brain.

[bb0145] Gorno-Tempini M.L., Rankin K.P., Woolley J.D., Rosen H.J., Phengrasamy L., Miller B.L. (2004). Cognitive and behavioral profile in a case of right anterior temporal lobe neurodegeneration. Cortex.

[bb0150] Gorno-Tempini M.L., Hillis A.E., Weintraub S., Kertesz A., Mendez M., Cappa S.F., Grossman M. (2011). Classification of primary progressive aphasia and its variants. Neurology.

[bb0155] Guo C.C., Gorno-Tempini M.L., Gesierich B., Henry M., Trujillo A., Shany-Ur T., Seeley W.W. (2013). Anterior temporal lobe degeneration produces widespread network-driven dysfunction. Brain.

[bb0160] Hamsher K.D., Roberts R.J. (1985). Memory for recent U.S. presidents in patients with cerebral disease. J. Clin. Exp. Neuropsychol..

[bb0165] Haxby J.V., Gobbini M.I., Calder A.J., Rhodes G., Johnson M. (2011). Distributed neural systems for face perception. The Oxford Handbook of Face Perception.

[bb0170] Hirstein W., Ramachandran V.S. (1997). Capgras syndrome: a novel probe for understanding the neural representation of the identity and familiarity of persons. Proc. Biol. Sci..

[bb0175] Hodges J.R., Salmon D.P., Butters N. (1993). Recognition and naming of famous faces in Alzheimer's disease: a cognitive analysis. Neuropsychologia.

[bb0180] Hsieh S., Hodges J.R., Piguet O. (2013). Recognition of positive vocalizations is impaired in behavioral-variant frontotemporal dementia. J. Int. Neuropsychol. Soc..

[bb0185] Kamminga J., Kumfor F., Burrell J.R., Piguet O., Hodges J.R., Irish M. (2014). Differentiating between right-lateralised semantic dementia and behavioural-variant frontotemporal dementia: an examination of clinical characteristics and emotion processing. J. Neurol. Neurosurg. Psychiatry.

[bb0190] Kaufmann J.M., Schweinberger S.R. (2004). Expression influences the recognition of familiar faces. Perception.

[bb0195] Keane J., Calder A.J., Hodges J.R., Young A.W. (2002). Face and emotion processing in frontal variant frontotemporal dementia. Neuropsychologia.

[bb0200] Kipps C.M., Nestor P.J., Acosta-Cabronero J., Arnold R., Hodges J.R. (2009). Understanding social dysfunction in the behavioural variant of frontotemporal dementia: the role of emotion and sarcasm processing. Brain.

[bb0205] Kumfor F., Piguet O. (2012). Disturbance of emotion processing in frontotemporal dementia: a synthesis of cognitive and neuroimaging findings. Neuropsychol. Rev..

[bb0210] Kumfor F., Miller L., Lah S., Hsieh S., Savage S., Hodges J.R., Piguet O. (2011). Are you really angry? The effect of intensity on facial emotion recognition in frontotemporal dementia. Soc. Neurosci..

[bb0215] Kumfor F., Irish M., Hodges J.R., Piguet O. (2013). Discrete neural correlates for the recognition of negative emotions: insights from frontotemporal dementia. PLoS One.

[bb0220] Kumfor F., Sapey-Triomphe L.A., Leyton C.E., Burrell J.R., Hodges J.R., Piguet O. (2014). Degradation of emotion processing ability in corticobasal syndrome and Alzheimer's disease. Brain.

[bb0225] Kumfor F., Irish M., Leyton C., Miller L., Lah S., Devenney E., Piguet O. (2014). Tracking the progression of social cognition in neurodegenerative disorders. J. Neurol. Neurosurg. Psychiatry.

[bb0230] Kumfor F., Hutchings R., Irish M., Hodges J.R., Rhodes G., Palermo R., Piguet O. (2015). Do I know you? Examining face and object memory in frontotemporal dementia. Neuropsychologia.

[bb0235] Lavenu I., Pasquier F., Lebert F., Petit H., Van der Linden M. (1999). Perception of emotion in frontotemporal dementia and Alzheimer disease. Alzheimer Dis. Assoc. Disord..

[bb0240] Levy Y., Bentin S. (2008). Interactive processes in matching identity and expressions of unfamiliar faces: evidence for mutual facilitation effects. Perception.

[bb0245] Lough S., Kipps C.M., Treise C., Watson P., Blair J.R., Hodges J.R. (2006). Social reasoning, emotion and empathy in frontotemporal dementia. Neuropsychologia.

[bb0250] Meng M., Cherian T., Singal G., Sinha P. (2012). Lateralization of face processing in the human brain. Proc. Biol. Sci..

[bb0255] Miller L.A., Hsieh S., Lah S., Savage S., Hodges J.R., Piguet O. (2012). One size does not fit all: face emotion processing impairments in semantic dementia, behavioural-variant frontotemporal dementia and Alzheimer's disease are mediated by distinct cognitive deficits. Behav. Neurol..

[bb0260] Mummery C.J., Patterson K., Wise R.J., Vandenberghe R., Price C.J., Hodges J.R. (1999). Disrupted temporal lobe connections in semantic dementia. Brain.

[bb0265] Neary D., Snowden J.S., Gustafson L., Passant U., Stuss D., Black S., Benson D.F. (1998). Frontotemporal lobar degeneration: a consensus on clinical diagnostic criteria. Neurology.

[bb0270] Oldfield R.C. (1971). The assessment and analysis of handedness: the Edinburgh inventory. Neuropsychologia.

[bb0275] Oliver L.D., Virani K., Finger E.C., Mitchell D.G. (2014). Is the emotion recognition deficit associated with frontotemporal dementia caused by selective inattention to diagnostic facial features?. Neuropsychologia.

[bb0280] Omar R., Henley S.M., Bartlett J.W., Hailstone J.C., Gordon E., Sauter D.A., Warren J.D. (2011). The structural neuroanatomy of music emotion recognition: evidence from frontotemporal lobar degeneration. NeuroImage.

[bb0285] Omar R., Rohrer J.D., Hailstone J.C., Warren J.D. (2011). Structural neuroanatomy of face processing in frontotemporal lobar degeneration. J. Neurol. Neurosurg. Psychiatry.

[bb0290] Rascovsky K., Hodges J.R., Knopman D., Mendez M.F., Kramer J.H., Neuhaus J., Miller B.L. (2011). Sensitivity of revised diagnostic criteria for the behavioural variant of frontotemporal dementia. Brain.

[bb0295] Rosen H.J., Perry R.J., Murphy J., Kramer J.H., Mychack P., Schuff N., Miller B.L. (2002). Emotion comprehension in the temporal variant of frontotemporal dementia. Brain.

[bb0300] Rosen H.J., Pace-Savitsky K., Perry R.J., Kramer J.H., Miller B.L., Levenson R.W. (2004). Recognition of emotion in the frontal and temporal variants of frontotemporal dementia. Dement. Geriatr. Cogn. Disord..

[bb0305] Seeley W.W., Crawford R., Rascovsky K., Kramer J.H., Weiner M., Miller B.L., Gorno-Tempini M.L. (2008). Frontal paralimbic network atrophy in very mild behavioral variant frontotemporal dementia. Arch. Neurol..

[bb0310] Smith C.N., Jeneson A., Frascino J.C., Kirwan C.B., Hopkins R.O., Squire L.R. (2014). When recognition memory is independent of hippocampal function. Proc. Natl. Acad. Sci. U. S. A..

[bb0315] Snowden J.S., Thompson J.C., Neary D. (2004). Knowledge of famous faces and names in semantic dementia. Brain.

[bb0320] Snowden J.S., Austin N.A., Sembi S., Thompson J.C., Craufurd D., Neary D. (2008). Emotion recognition in Huntington's disease and frontotemporal dementia. Neuropsychologia.

[bb0325] Snowden J.S., Thompson J.C., Neary D. (2012). Famous people knowledge and the right and left temporal lobes. Behav. Neurol..

[bb0330] Stanislaw H., Todorov N. (1999). Calculation of signal detection theory measures. Behav. Res. Methods Instrum. Comput..

[bb0335] Van den Stock J., de Gelder B. (2012). Emotional information in body and background hampers recognition memory for faces. Neurobiol. Learn. Mem..

[bb0340] Van den Stock J., de Gelder B. (2014). Face identity matching is influenced by emotions conveyed by face and body. Front. Hum. Neurosci..

[bb0345] Van den Stock J., van de Riet W.A., Righart R., de Gelder B. (2008). Neural correlates of perceiving emotional faces and bodies in developmental prosopagnosia: an event-related fMRI-study. PLoS One.

[bb0350] Van den Stock J., de Gelder B., De Winter F.L., Van Laere K., Vandenbulcke M. (2012). A strange face in the mirror. face-selective self-misidentification in a patient with right lateralized occipito-temporal hypo-metabolism. Cortex.

[bb0355] Van den Stock J., Vandenbulcke M., Zhu Q., Hadjikhani N., de Gelder B. (2012). Developmental prosopagnosia in a patient with hypoplasia of the vermis cerebelli. Neurology.

[bb0360] Van den Stock J., de Gelder B., Van Laere K., Vandenbulcke M. (2013). Face-selective hyper-animacy and hyper-familiarity misperception in a patient with moderate Alzheimer's disease. J. Neuropsychiatr. Clin. Neurosci..

[bb0365] Von Der Heide R.J., Skipper L.M., Olson I.R. (2013). Anterior temporal face patches: a meta-analysis and empirical study. Front. Hum. Neurosci..

[bb0370] Werner K.H., Roberts N.A., Rosen H.J., Dean D.L., Kramer J.H., Weiner M.W., Levenson R.W. (2007). Emotional reactivity and emotion recognition in frontotemporal lobar degeneration. Neurology.

[bb0375] Whitwell J.L., Przybelski S.A., Weigand S.D., Ivnik R.J., Vemuri P., Gunter J.L., Josephs K.A. (2009). Distinct anatomical subtypes of the behavioural variant of frontotemporal dementia: a cluster analysis study. Brain.

