# Supplementary material for: Face shape and face identity processing in behavioral variant fronto-temporal dementia: A specific deficit for familiarity and name recognition of famous faces
Source: Neuroimage Clin. 2016 Mar 10;11:368–77. doi: 10.1016/j.nicl.2016.03.001 (PMC4893012; doi:10.1016/j.nicl.2016.03.001)
Supplement: Table S1 — . Case summaries of demographic and behavioral data. YDD = disease duration based on heteroanamnesis; MMSE = Mini-Mental State Examination; A1-A5 = sum of scores on trials A1 to A5 of the RAVLT (Rey’s Auditory Verbal Learning Test); %Recall = [score on trial A7 (delayed recall) / (maximum of trials A1 to A5) of the RAVLT]*100; Recog = correct hits − false hits on trial A8 (recognition) of the RAVLT; TMT = Trail Making Test; AVF = Animal Verbal Fluency (1 minute); RCPMT = Raven's Colored Progressive Matrices Test (sets A & B); Compr = Score on Comprehension subtest of the Aachen Aphasia Test; BNT = Boston Naming Test; BORB = Birmingham Object Recognition Battery; Le = Length matching; Si = Size matching; Or = Orientation matching; DiagnCrit_A = Diagnostic Criterium (Rascovsky et al., 2011); A = Early behavioral disinhibition; B = Early apathy or inertia; C = Early loss of sympathy or empathy; D = Early perseverative, stereotyped or compulsive/ritualistic behavior; E = Hyperorality and dietary changes; F = Neuropsychological profile: executive/generation deficits with relative sparing of memory and visuospatial functions. [file mmc1.docx]

Table S1. Case summaries of demographic and behavioral data. YDD=disease duration based on heteroanamnesis; MMSE=Mini-Mental State Examination; A1-A5= sum of scores on trials A1 to A5 of the RAVLT (Rey’s Auditory Verbal Learning Test); %Recall=[score on trial A7 (delayed recall)/(maximum of trials A1 to A5) of the RAVLT]*100; Recog= correct hits – false hits on trial A8 (recognition) of the RAVLT; TMT = Trail Making Test; AVF=Animal Verbal Fluency (1 minute); RCPMT= Raven’s Colored Progressive Matrices Test (sets A & B); Compr= Score on Comprehension subtest of the Aachen Aphasia Test; BNT= Boston Naming Test; BORB=Birmingham Object Recognition Battery; Le=Length matching; Si=Size matching; Or=Orientation matching; DiagnCrit_A=Diagnostic Criterium (Rascovsky, Hodges et al. 2011); A=Early behavioral disinhibition; B=Early apathy or inertia; C=Early loss of sympathy or empathy; D=Early perseverative, stereotyped or compulsive/ritualistic behavior; E=Hyperorality and dietary changes; F=Neuropsychological profile: executive/generation deficits with relative sparing of memory and visuospatial functions.

|  | 1 | 2 | 3 | 4 | 5 | 6 | 7 | 8 | 9 | 10 | 11 | 12 | 13 | 14 | 15 | 16 | 17 | 18 | 19 | 20 | 21 | 22 | 23 |
| --- | --- | --- | --- | --- | --- | --- | --- | --- | --- | --- | --- | --- | --- | --- | --- | --- | --- | --- | --- | --- | --- | --- | --- |
| age | 59,3 | 58,9 | 48,7 | 70,2 | 56,3 | 40,3 | 69,2 | 63,6 | 76,6 | 63,2 | 73,0 | 77,6 | 59,1 | 65,0 | 82,4 | 61,9 | 55,0 | 67,2 | 67,0 | 54,5 | 70,0 | 71,2 | 73,7 |
| sex | ♀ | ♂ | ♂ | ♂ | ♂ | ♀ | ♂ | ♂ | ♂ | ♀ | ♀ | ♂ | ♀ | ♂ | ♀ | ♂ | ♀ | ♀ | ♂ | ♂ | ♀ | ♂ | ♀ |
| YDD | 2,0 | 2,0 | 1,0 | 0,5 | 1,0 | 0,5 | 0,5 | 2,0 | 3,0 | 4,0 | 3,0 | 1,0 | 2,0 | 2,0 | 4,0 | 2,0 | 2,0 | 2,0 | 1,0 | 2,0 | 3,0 | 4,0 | 2,0 |
| MMSE | 25 | na | 28 | na | 27 | 28 | 27 | 27 | 24 | 26 | 24 | 30 | 28 | 27 | 26 | 25 | 28 | 26 | 27 | 25 | 28 | 28 | 26 |
| A1-A5 | na | na | 11 | na | 18 | 59 | 26 | 26 | 15 | 29 | 27 | 39 | 26 | 32 | 29 | 21 | 47 | 38 | 37 | 26 | 26 | 32 | 15 |
| %Recall | na | na | 25 | na | 50 | 93 | 13 | 38 | 0 | 75 | 71 | 90 | 14 | 67 | 75 | 29 | 83 | 80 | 67 | 33 | 44 | 125 | 50 |
| Recog | na | na | 5 | na | -2 | 15 | 4 | 11 | 2 | 12 | -11 | 15 | 8 | 10 | 8 | -6 | 14 | 13 | 7 | -5 | 13 | 11 | 5 |
| TMT A | na | na | 56 | na | 56 | na | 40 | 29 | 218 | 53 | 53 | 51 | 31 | 70 | 84 | 48 | 25 | 106 | 74 | 43 | 43 | 44 | 83 |
| TMT B | na | na | 307 | na | na | na | 86 | 92 | na | 597 | na | 137 | 66 | 140 | 340 | na | 78 | 258 | 250 | 125 | 128 | 104 | 188 |
| AVF | na | na | 14 | na | 13 | 13 | 21 | 10 | 4 | 18 | 8 | 10 | 22 | 23 | 20 | 8 | 20 | 20 | 13 | 20 | 16 | 17 | 9 |
| RCPMT | na | na | 16 | na | 17 | 13 | 18 | 21 | 14 | 11 | 14 | 19 | 21 | 24 | 12 | 17 | 10 | 19 | 17 | 21 | 13 | 17 | 12 |
| Compr | na | na | 98 | na | 85 | na | 98 | 67 | 90 | 107 | 73 | 100 | 104 | 96 | 100 | 100 | 95 | 100 | 82 | 106 | 101 | 109 | 73 |
| BNT | na | na | 49 | na | 50 | 42 | 36 | 35 | 34 | 48 | 14 | 55 | 45 | 23 | 41 | 56 | 46 | 44 | 34 | 45 | 48 | 51 | 9 |
| BORB_Le | na | na | na | na | 83 | na | 83 | 90 | 80 | 87 | na | 93 | 97 | 83 | 89 | 80 | 73 | 97 | 100 | 93 | 93 | 87 | 80 |
| BORB_Si | na | na | na | na | 80 | na | 93 | 87 | 73 | 80 | na | 90 | 90 | 90 | 70 | 80 | 87 | 93 | 93 | 87 | 87 | 90 | 83 |
| BORB_Or | na | na | na | na | 83 | na | 88 | 83 | 53 | 83 | na | 83 | 87 | 87 | 77 | 70 | 83 | 80 | 87 | 93 | 87 | 87 | 73 |
| DiagnCrit_A | 1 | 1 | 1 | 1 | 1 | 1 | 1 | 1 | 1 | 1 | 1 | 1 | 1 | 1 | 1 | 1 | 1 | 1 | 1 | 1 | 1 | 0 | 1 |
| DiagnCrit_B | 1 | 1 | 0 | 0 | 0 | 0 | 1 | 1 | 1 | 1 | 1 | 1 | 1 | 0 | 1 | 1 | 1 | 1 | 1 | 1 | 1 | 1 | 1 |
| DiagnCrit_C | 1 | 1 | 1 | 1 | 1 | 1 | 1 | 1 | 1 | 1 | 1 | 1 | 1 | 1 | 1 | 1 | 1 | 1 | 1 | 1 | 1 | 1 | 1 |
| DiagnCrit_D | 1 | 1 | 1 | 1 | 1 | 1 | 0 | 0 | 0 | 0 | 1 | 0 | 1 | 1 | 0 | 0 | 0 | 1 | 0 | 0 | 0 | 0 | 0 |
| DiagnCrit_E | 0 | 0 | 0 | 1 | 1 | 1 | 0 | 0 | 1 | 1 | 0 | 0 | 0 | 1 | 0 | 0 | 1 | 1 | 1 | 0 | 1 | 0 | 0 |
| DiagnCrit_F | 1 | 0 | 1 | 1 | 1 | 1 | 0 | 1 | 1 | 1 | 0 | 1 | 0 | 1 | 0 | 0 | 1 | 1 | 1 | 0 | 1 | 1 | 1 |
