# Supplementary figures and images for: Face shape and face identity processing in behavioral variant fronto-temporal dementia: A specific deficit for familiarity and name recognition of famous faces
Source: Neuroimage Clin. 2016 Mar 10;11:368–77. doi: 10.1016/j.nicl.2016.03.001 (PMC4893012; doi:10.1016/j.nicl.2016.03.001)

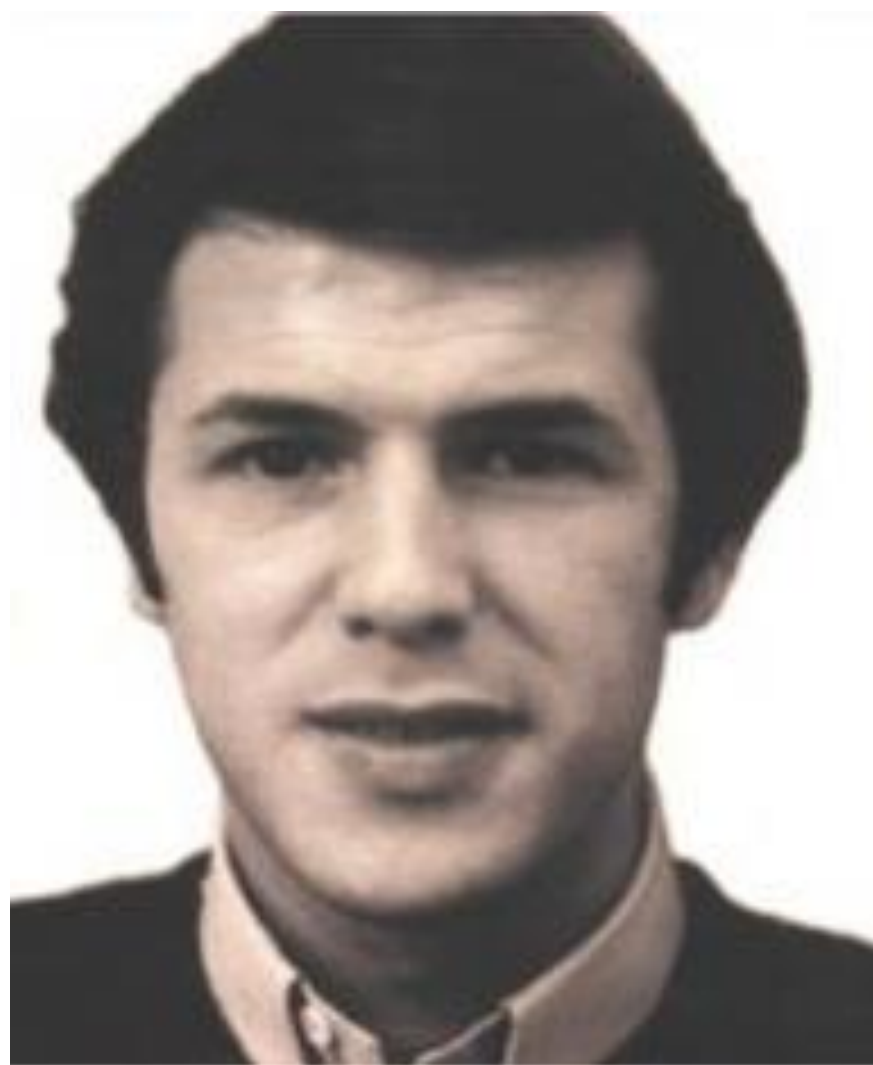

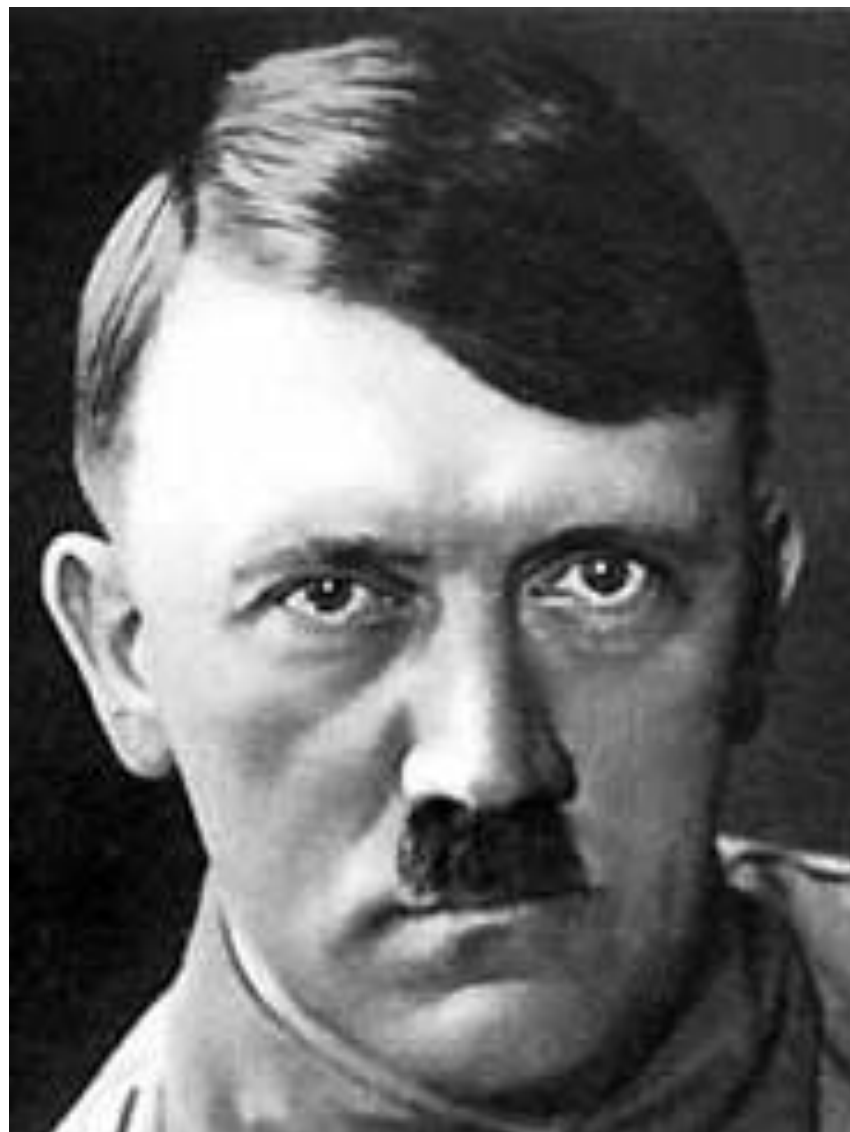

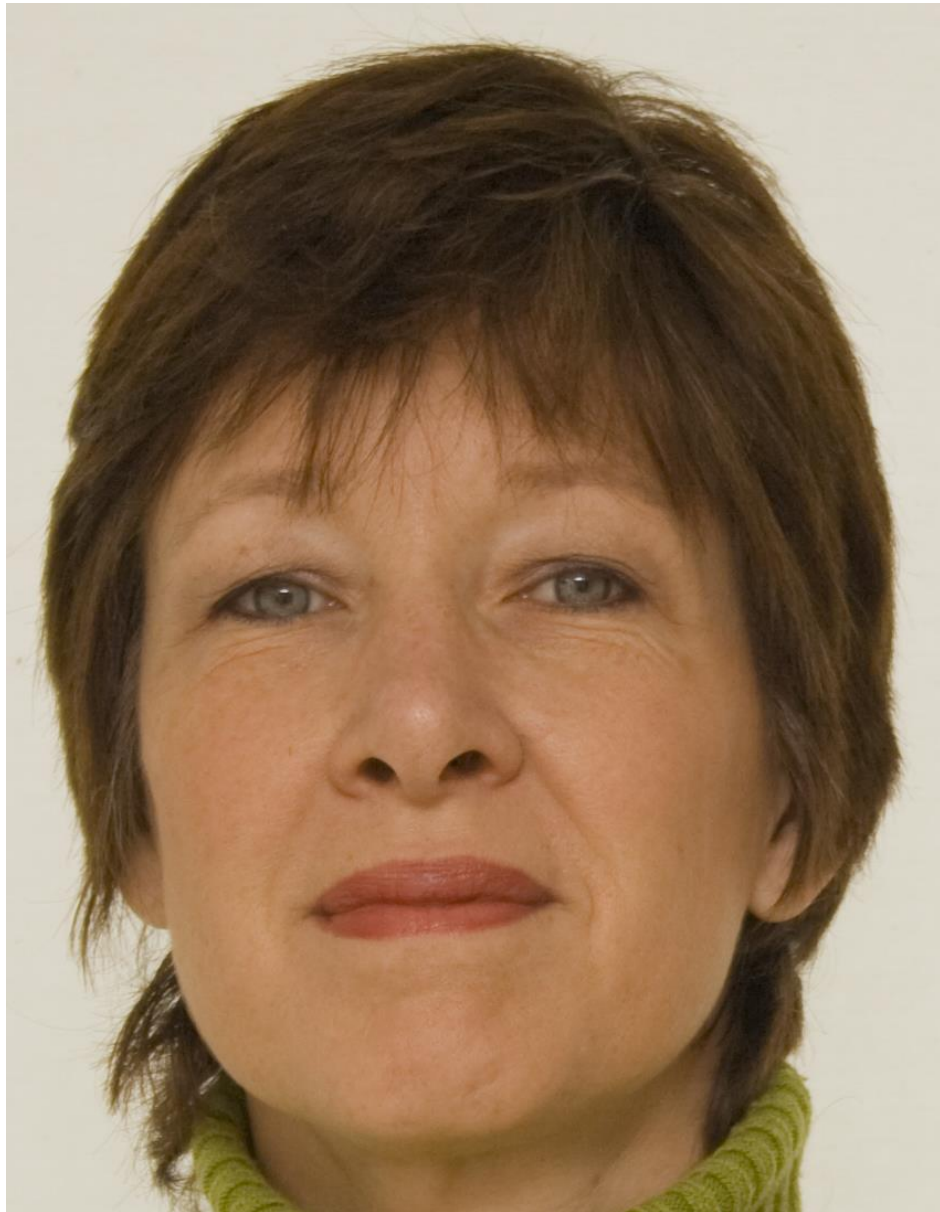

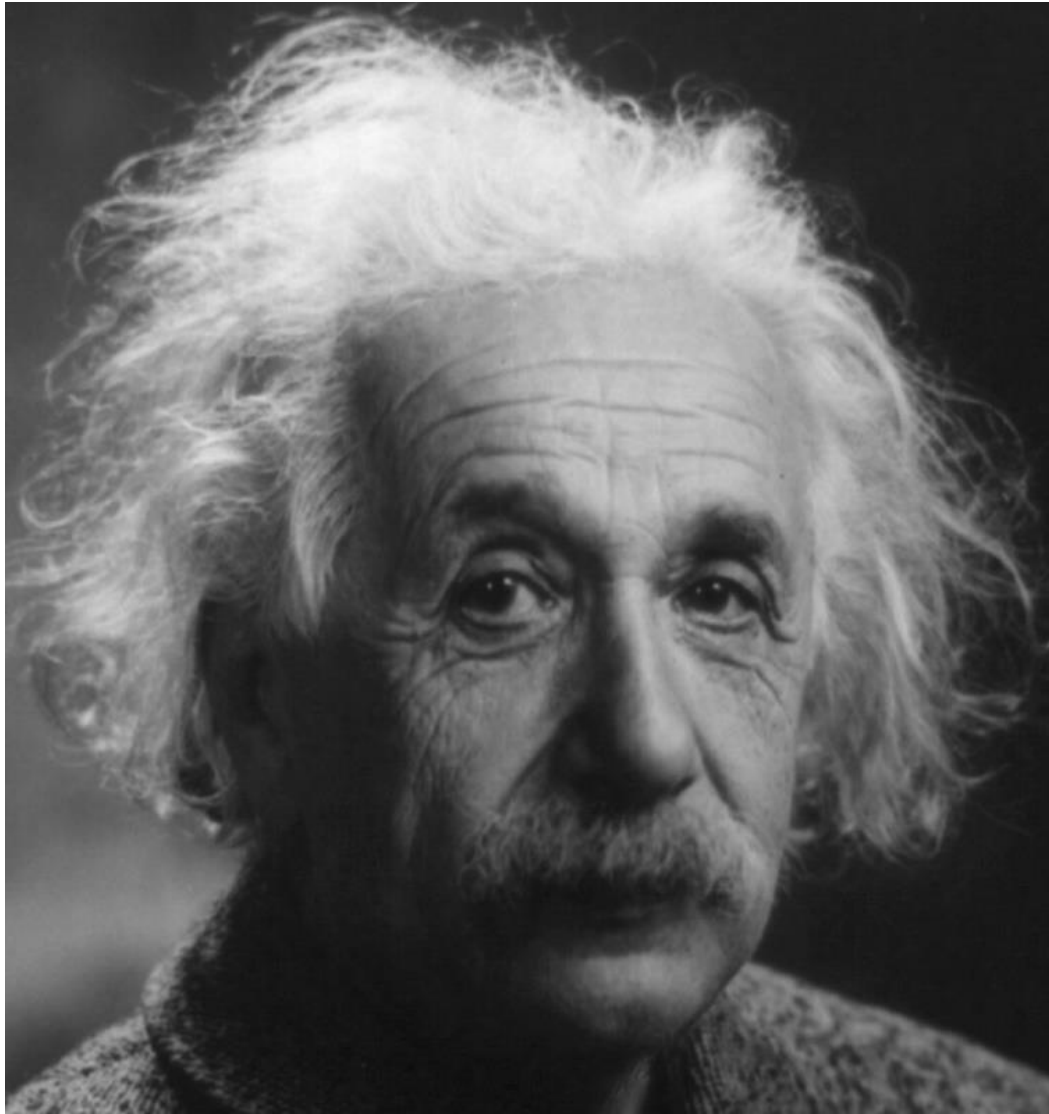

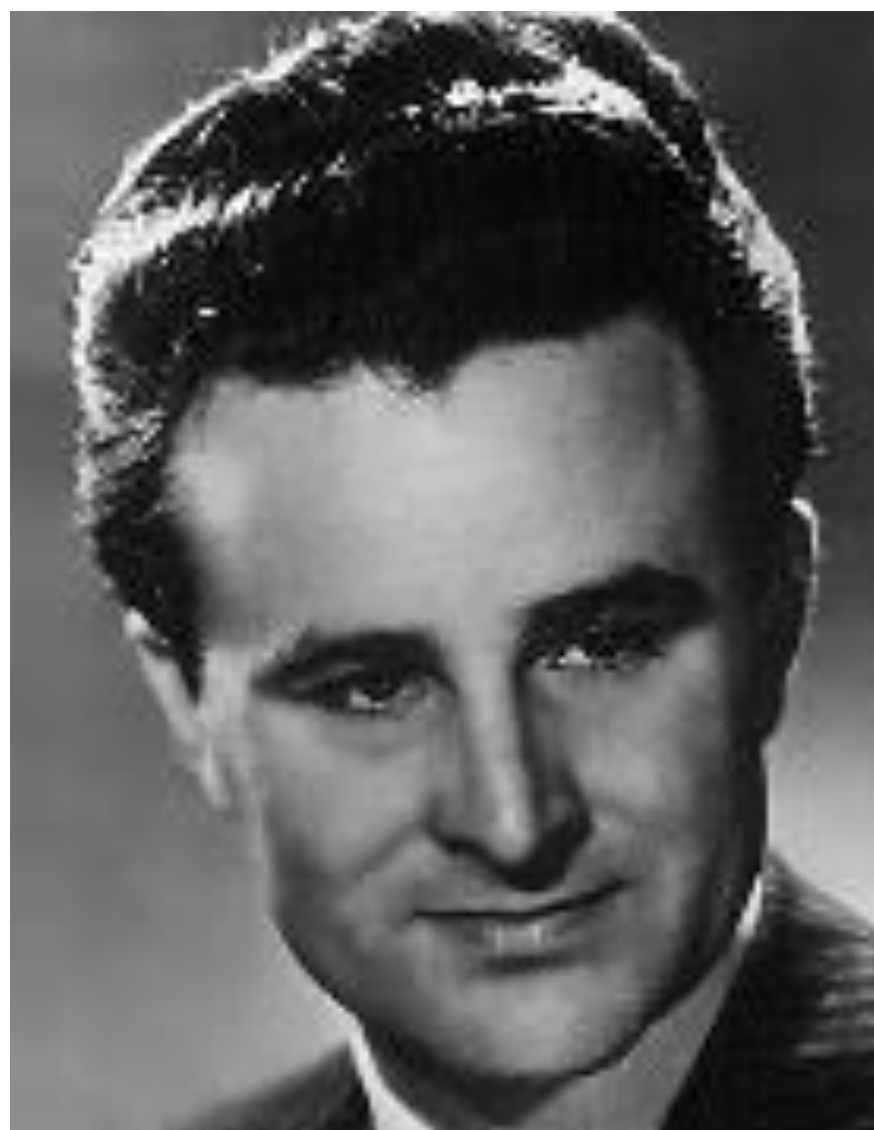

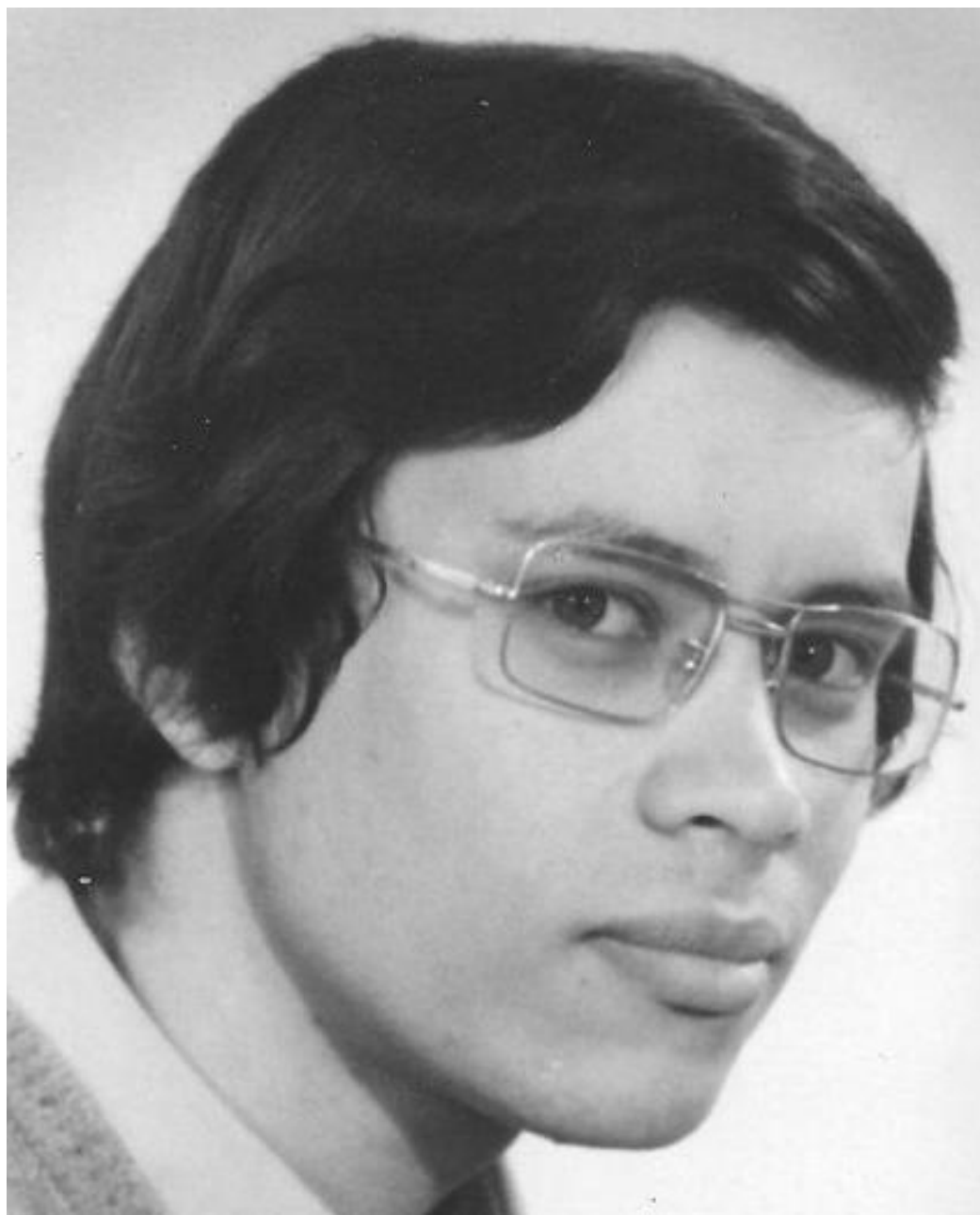

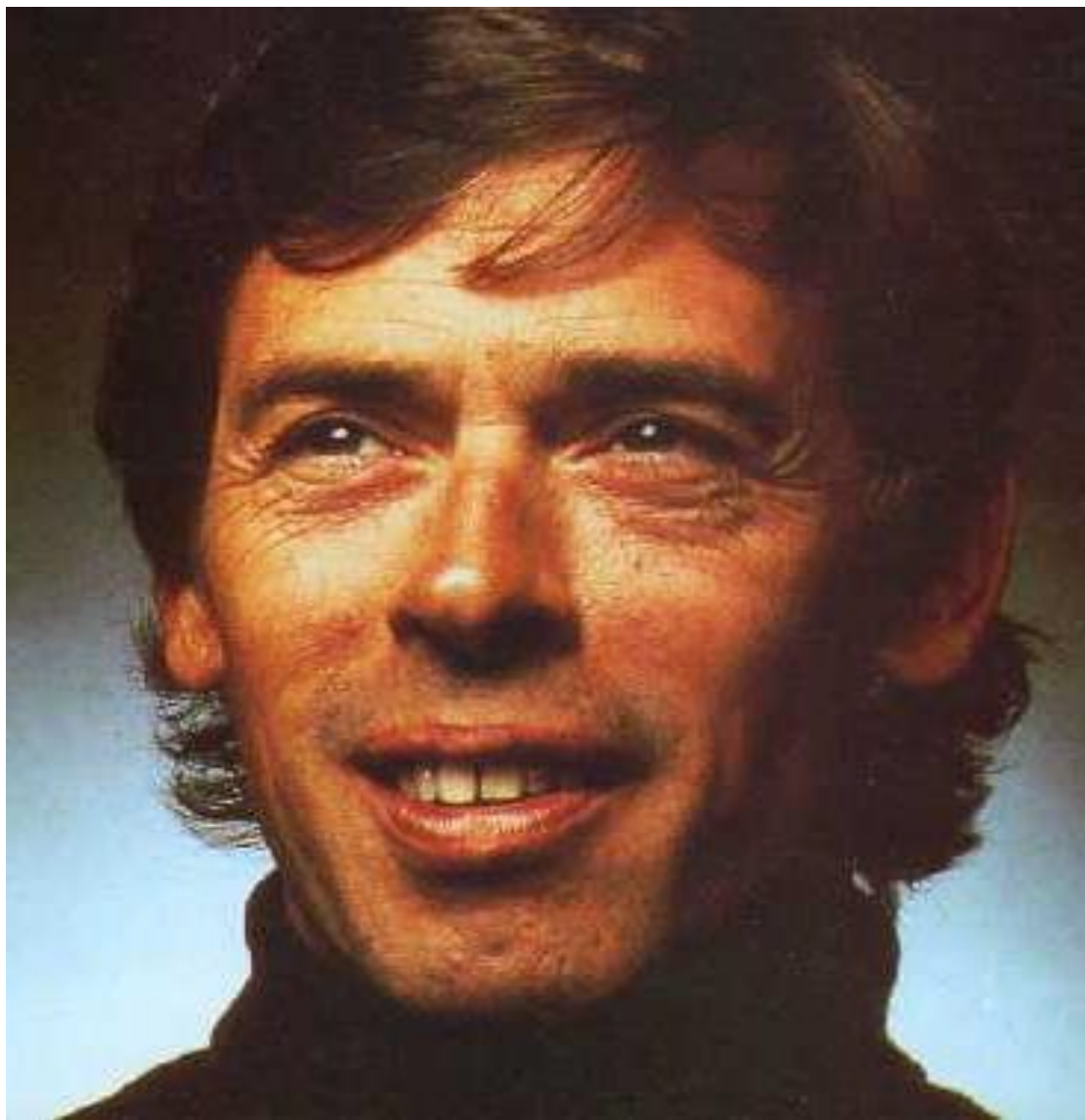

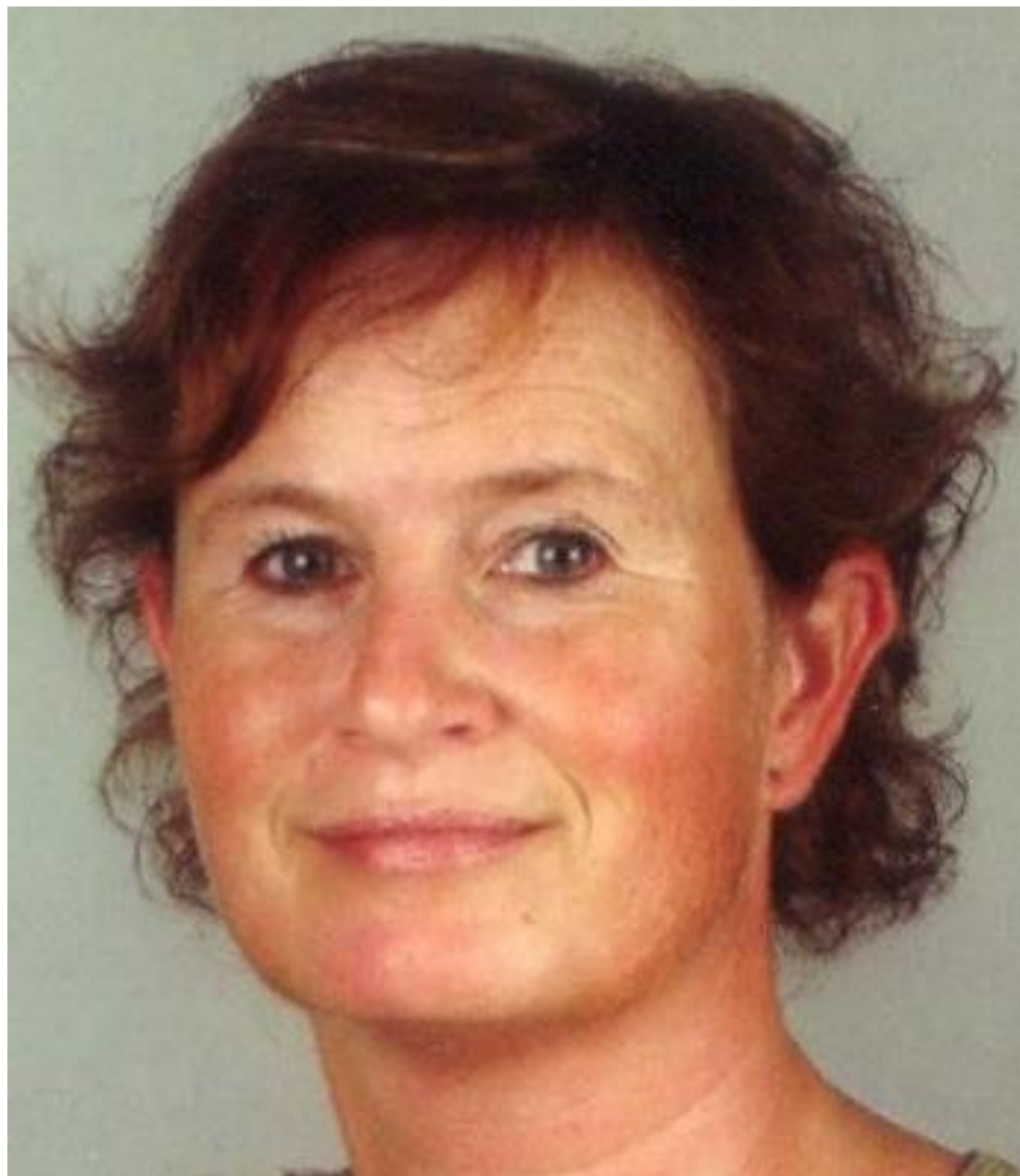

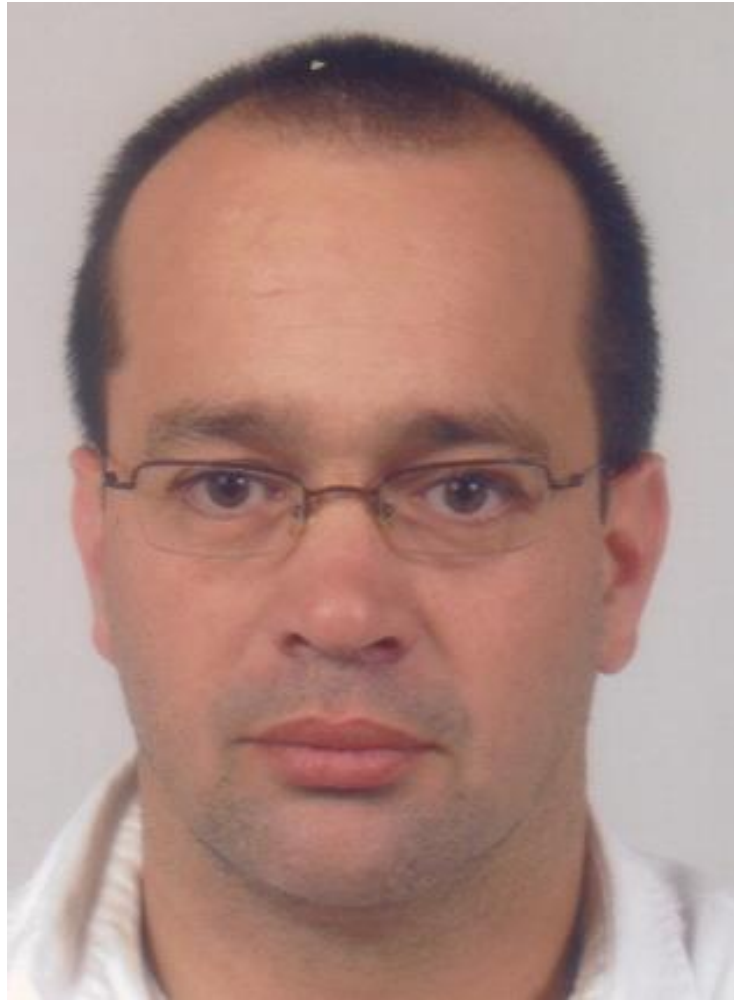

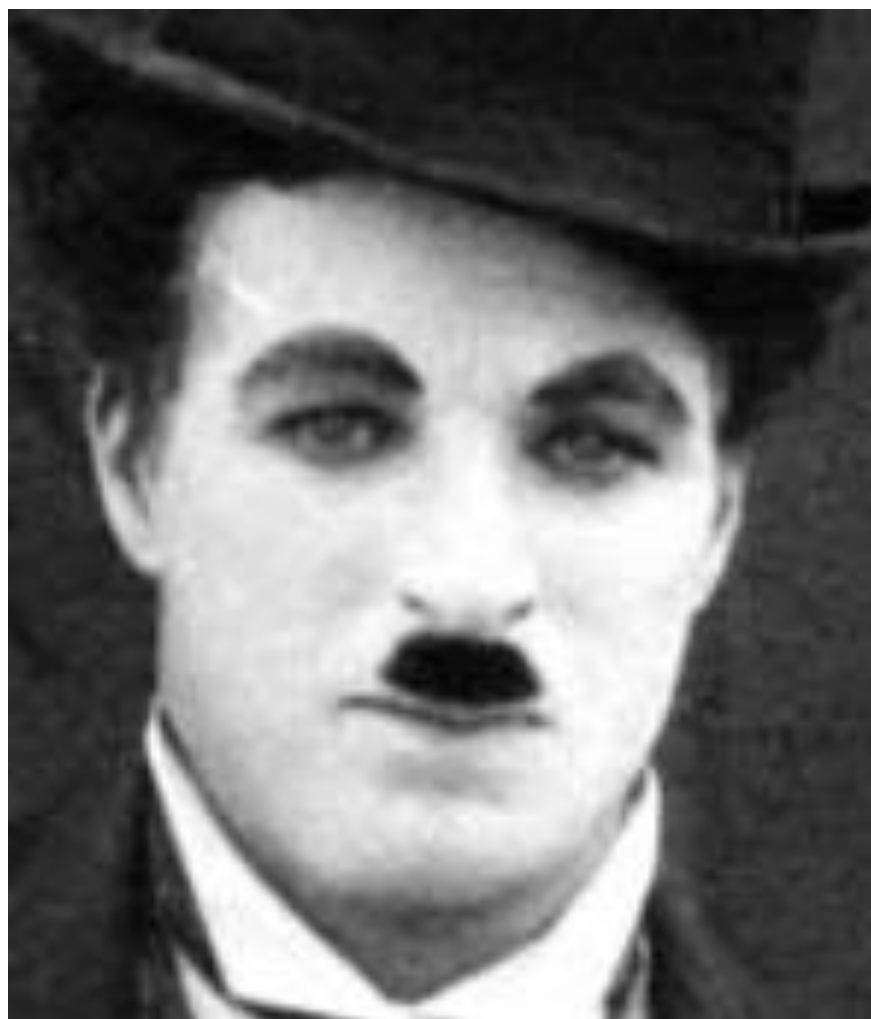

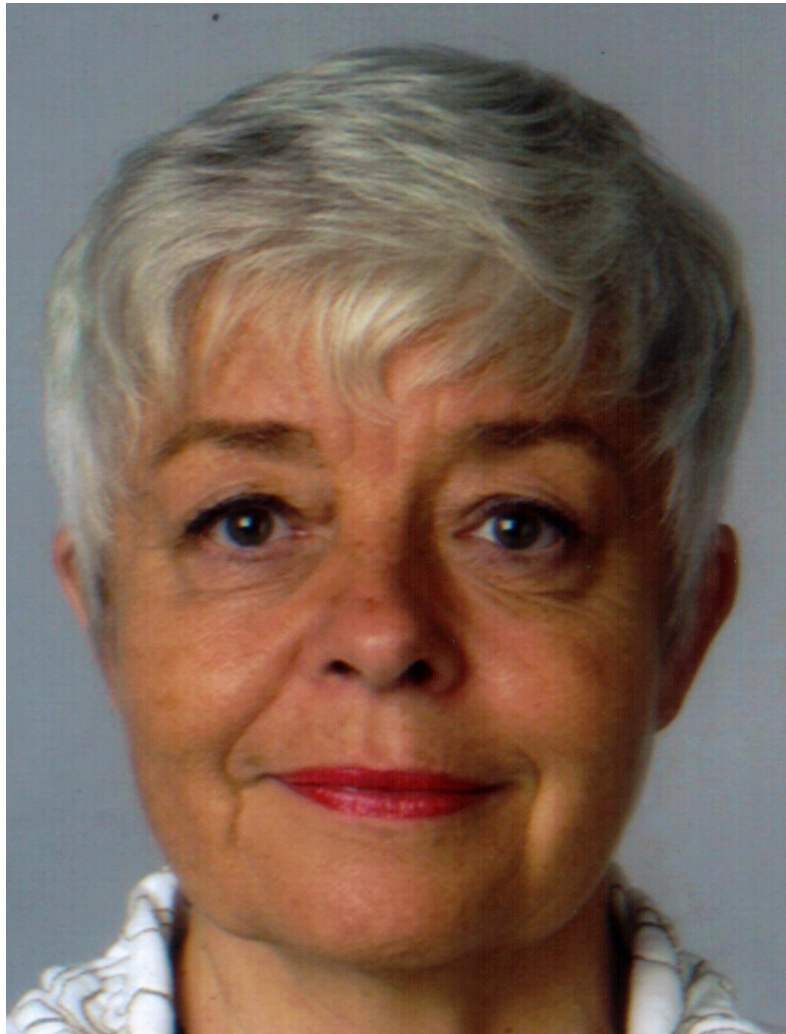

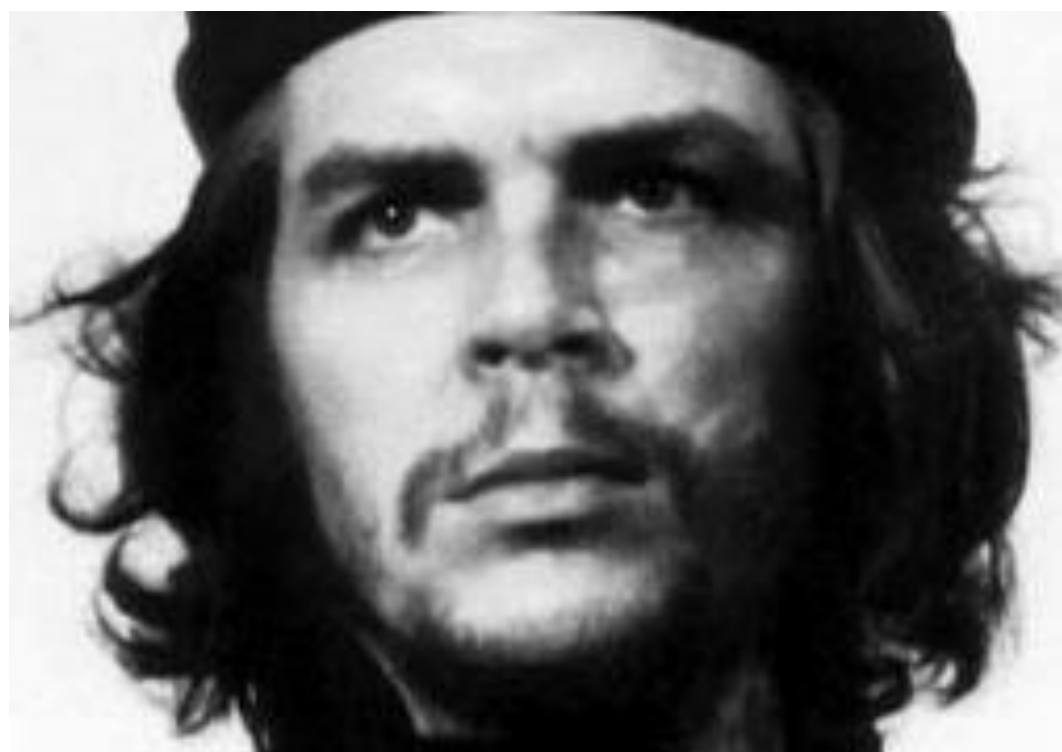

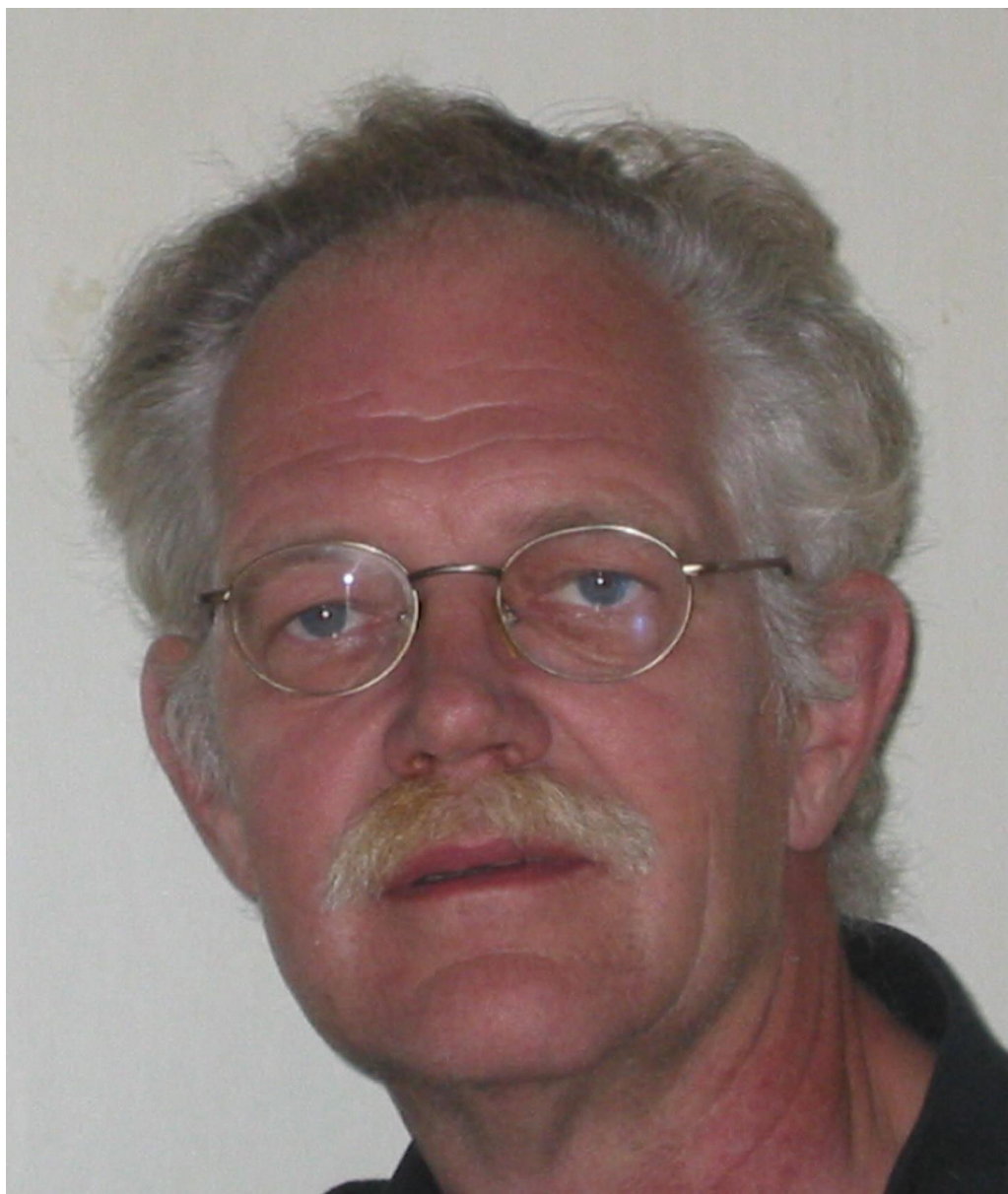

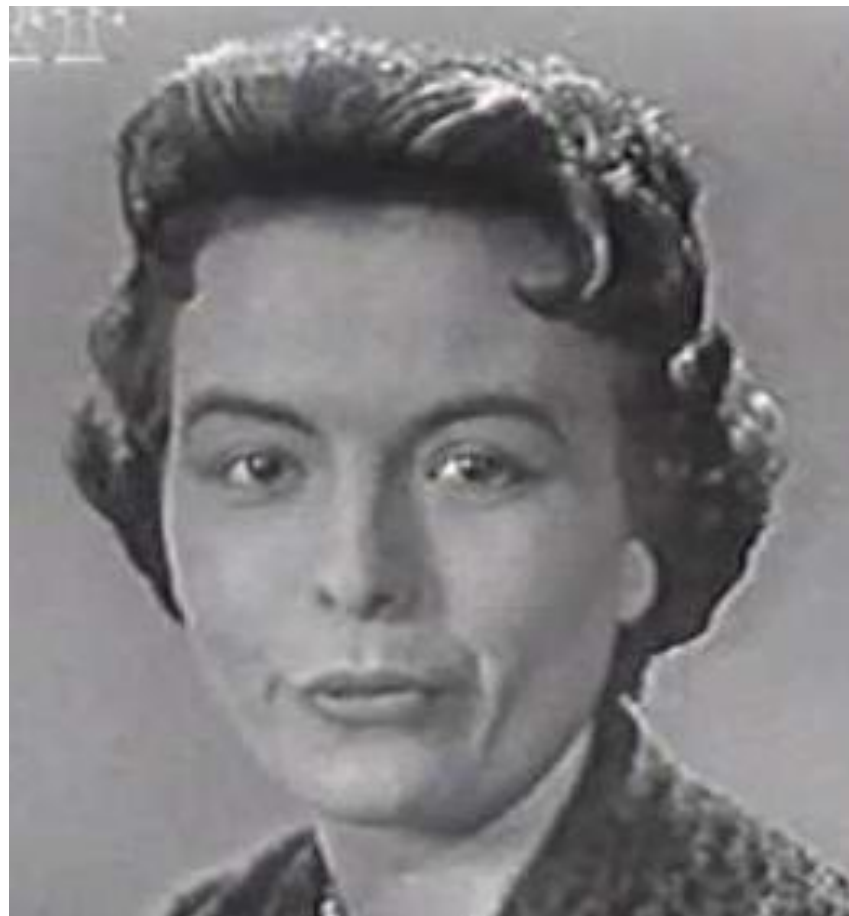

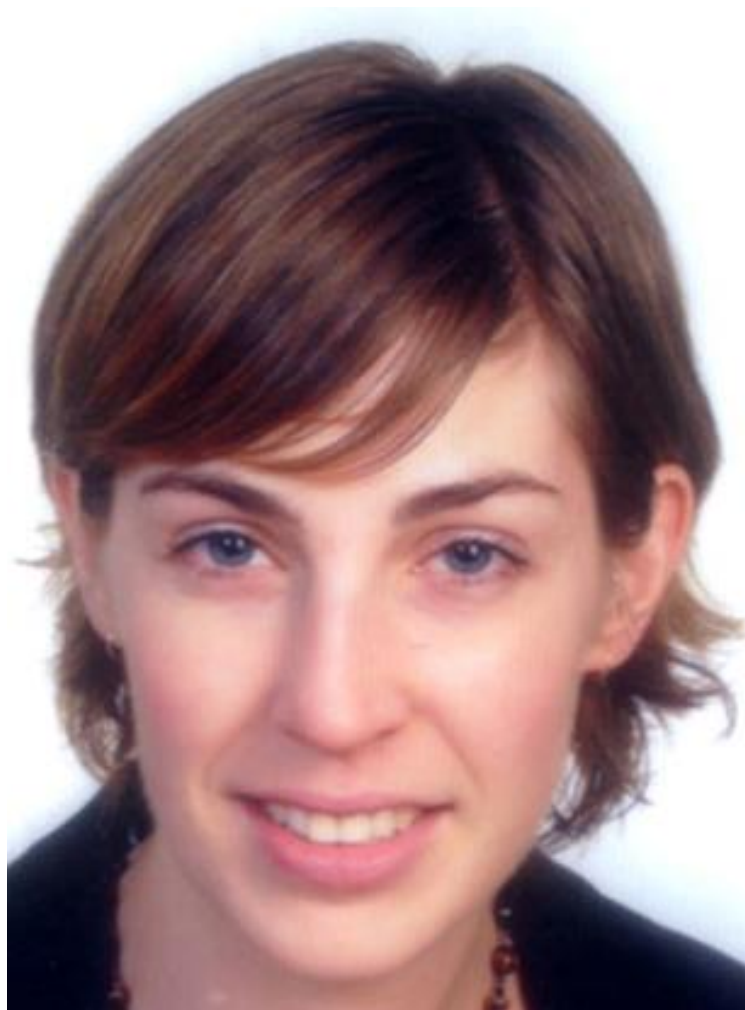

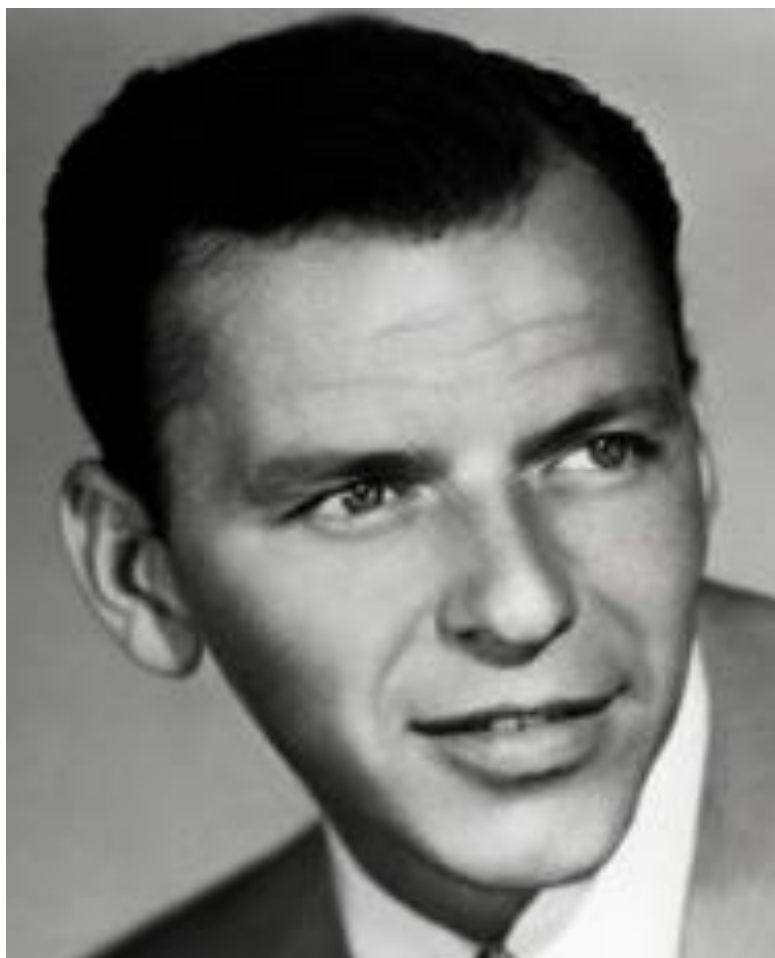

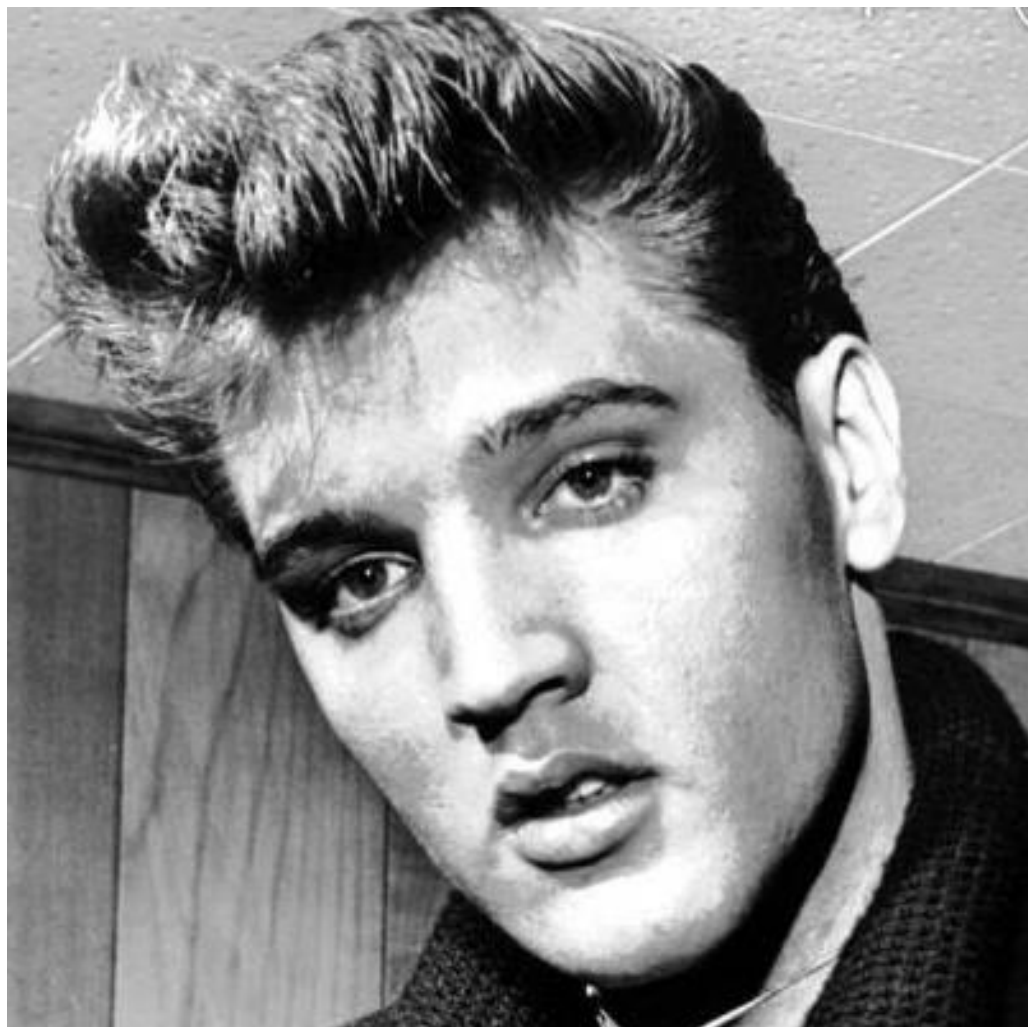

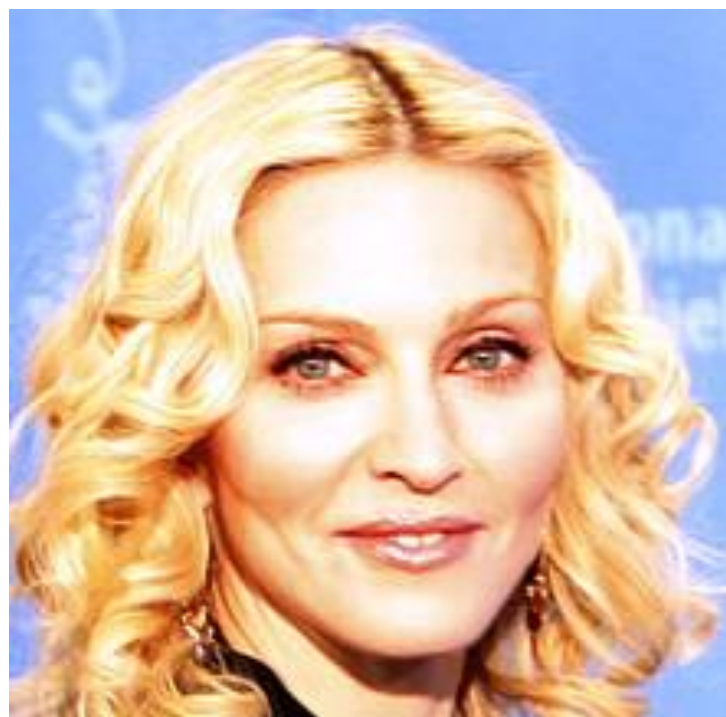

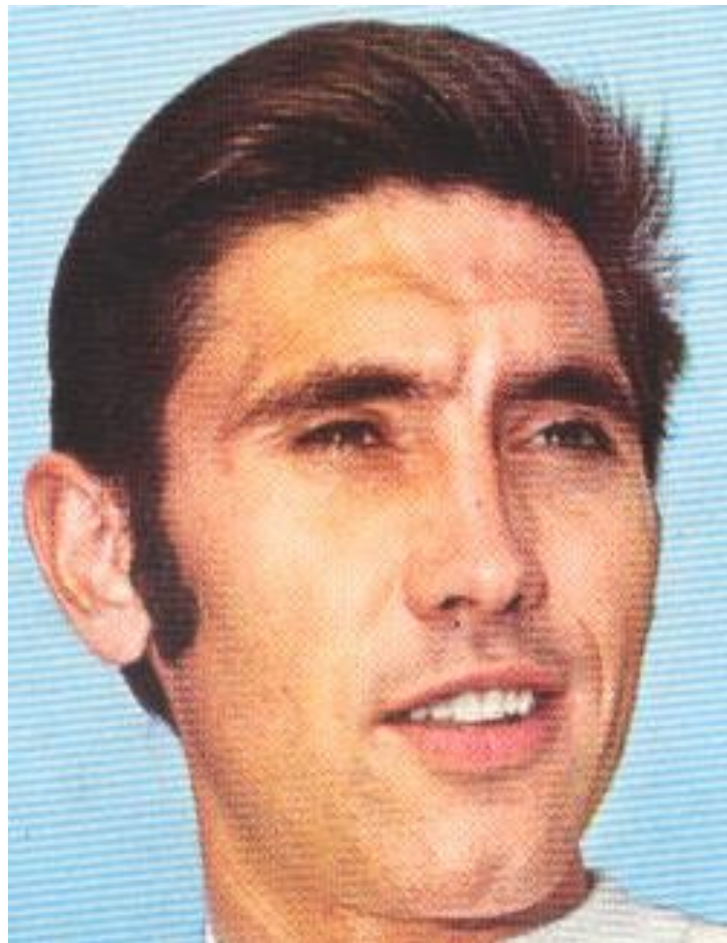

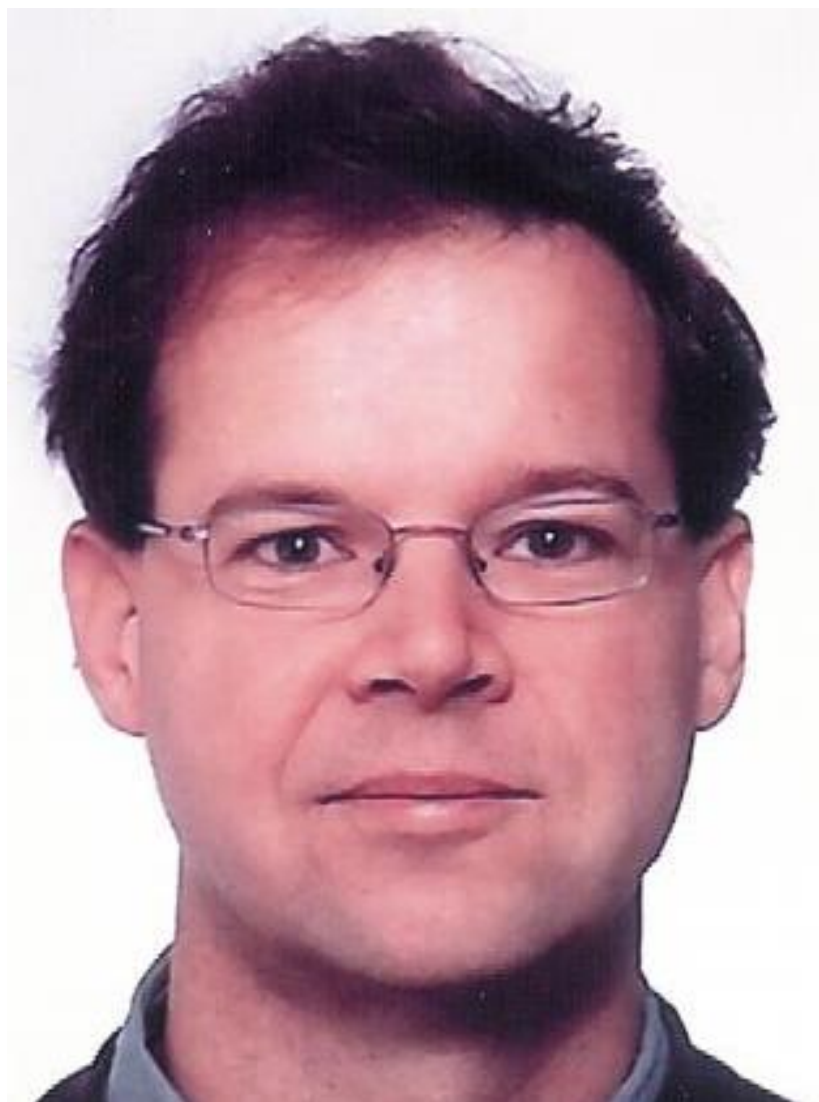

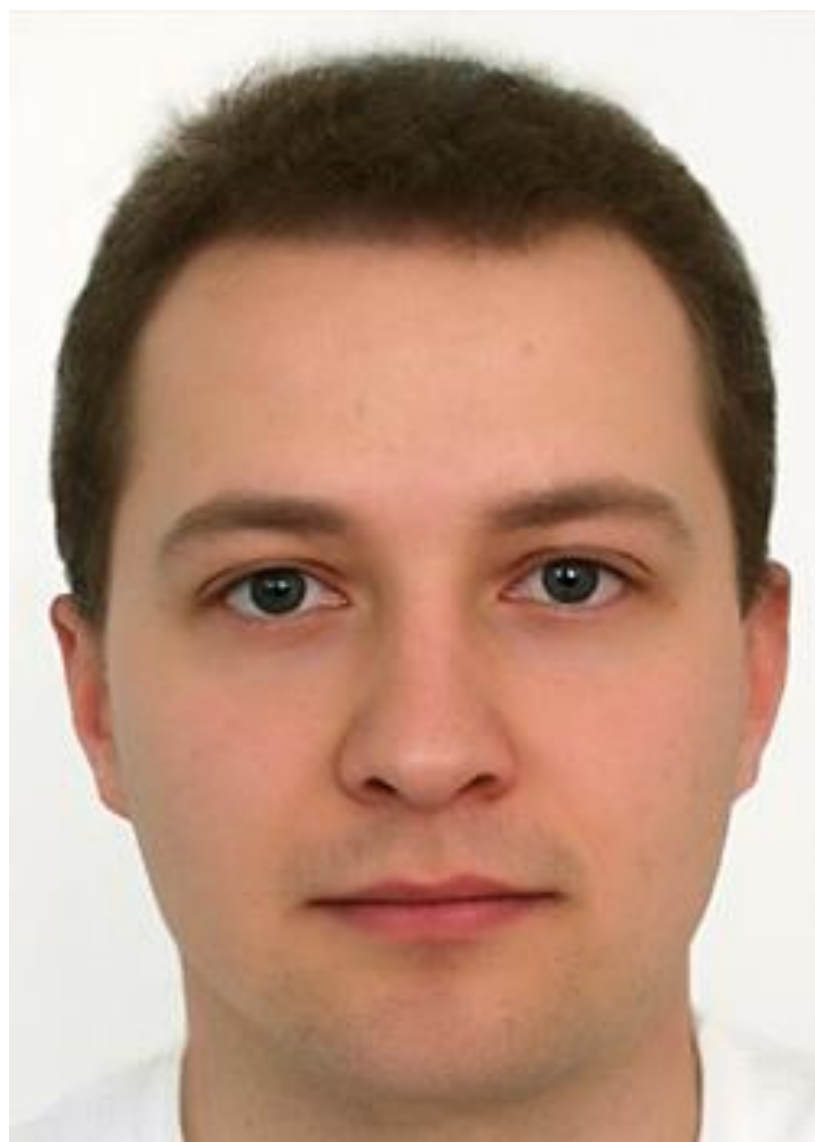

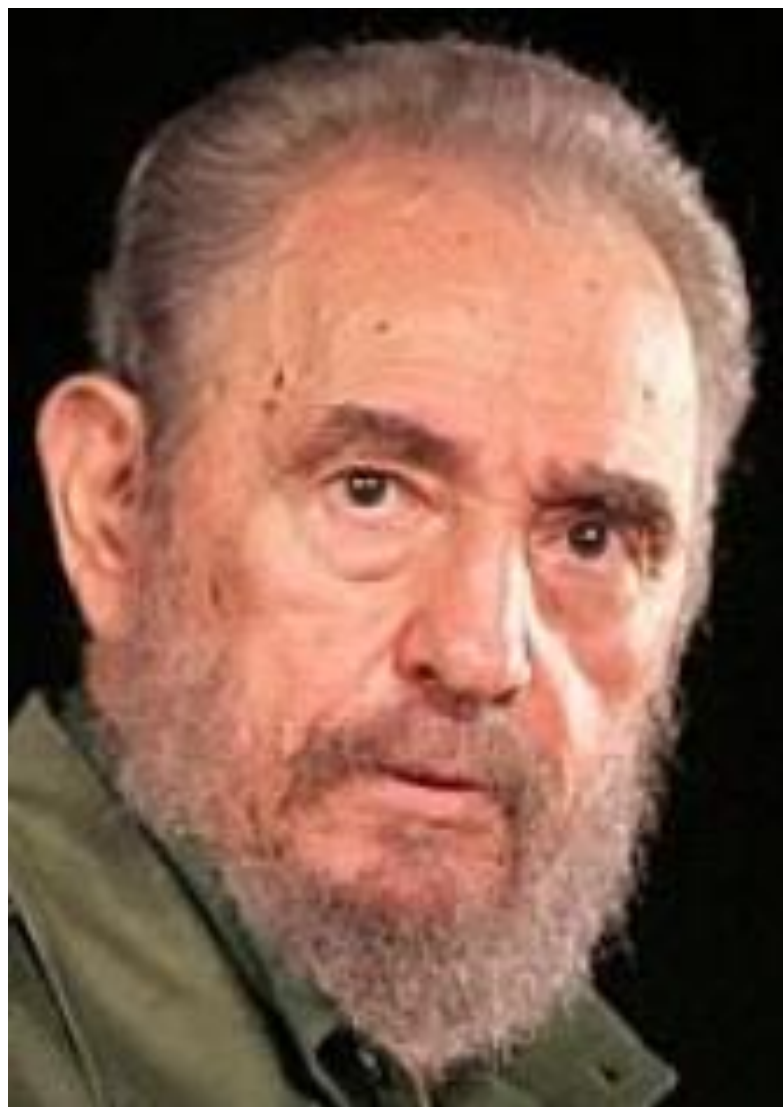

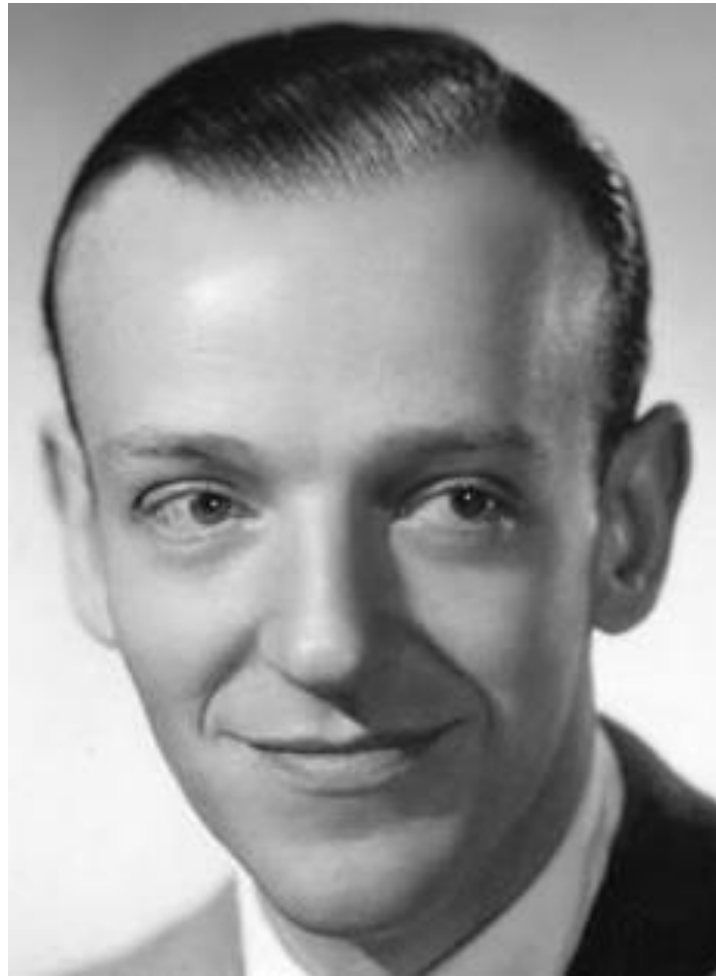

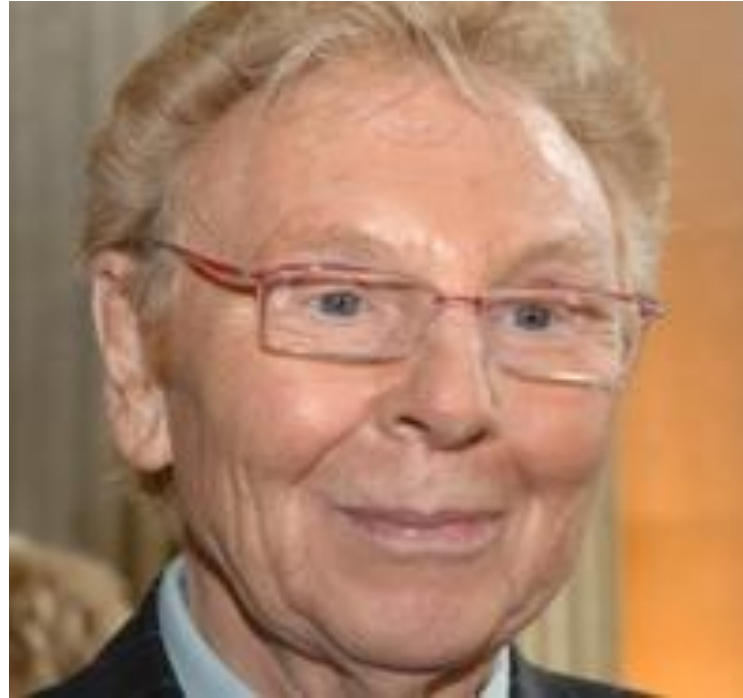

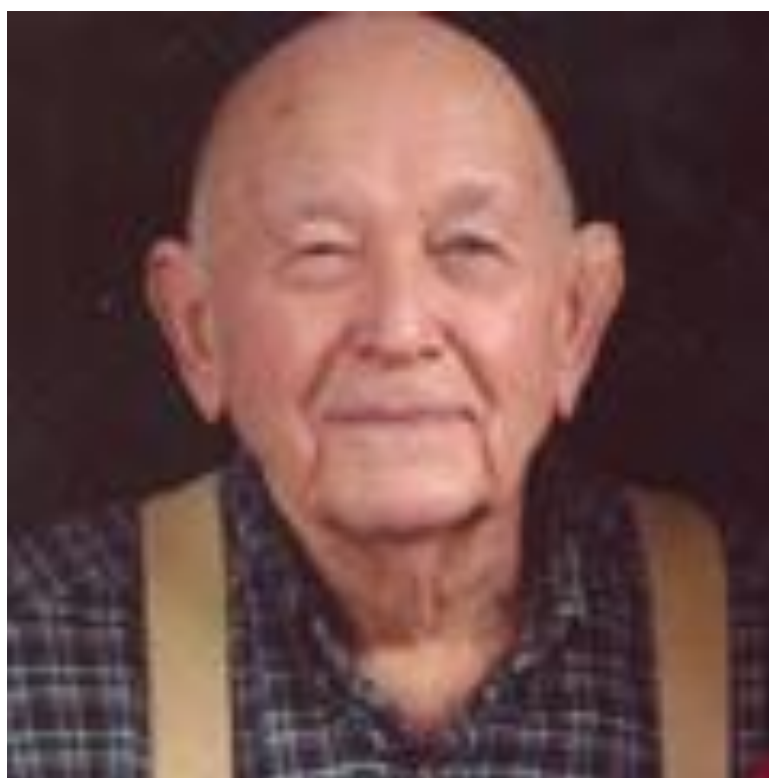

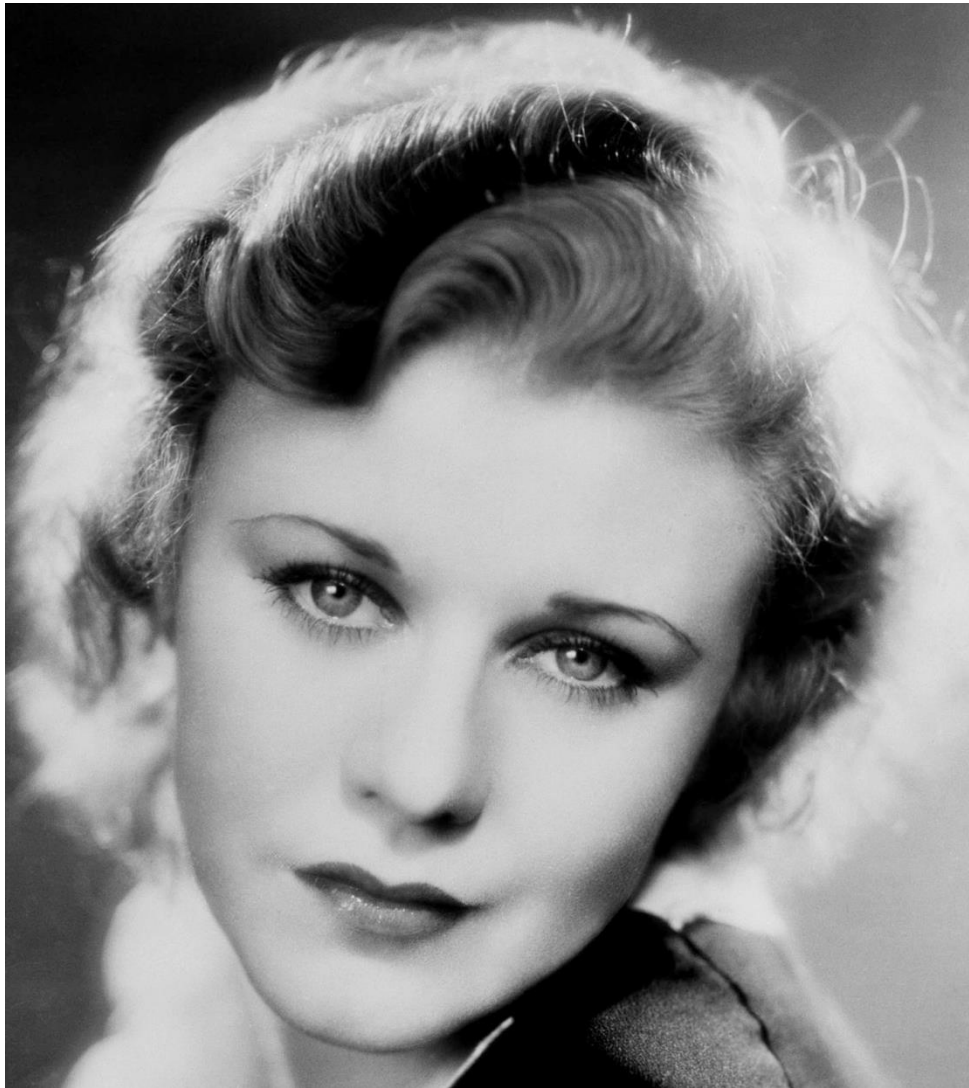

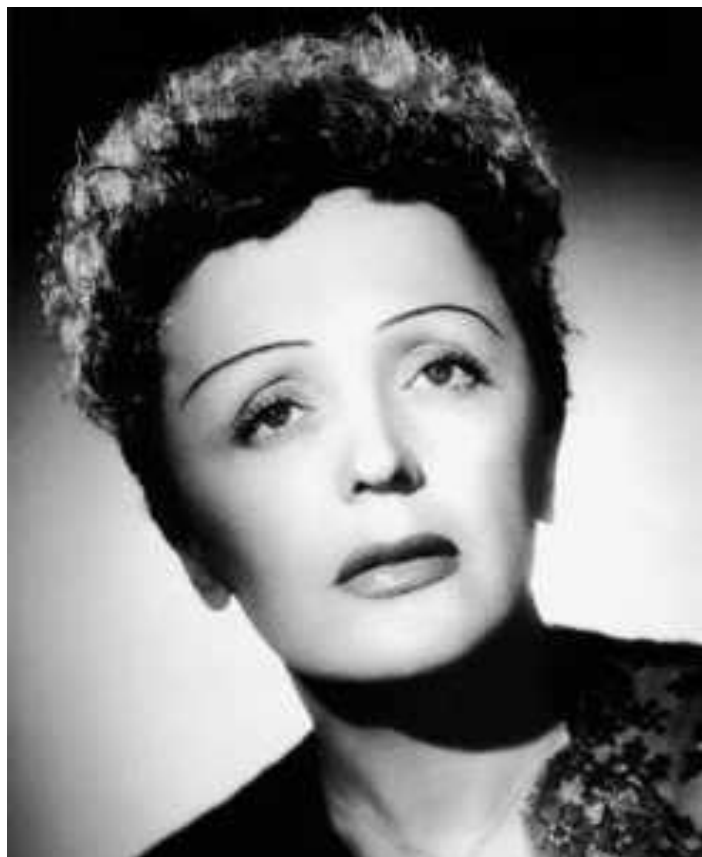

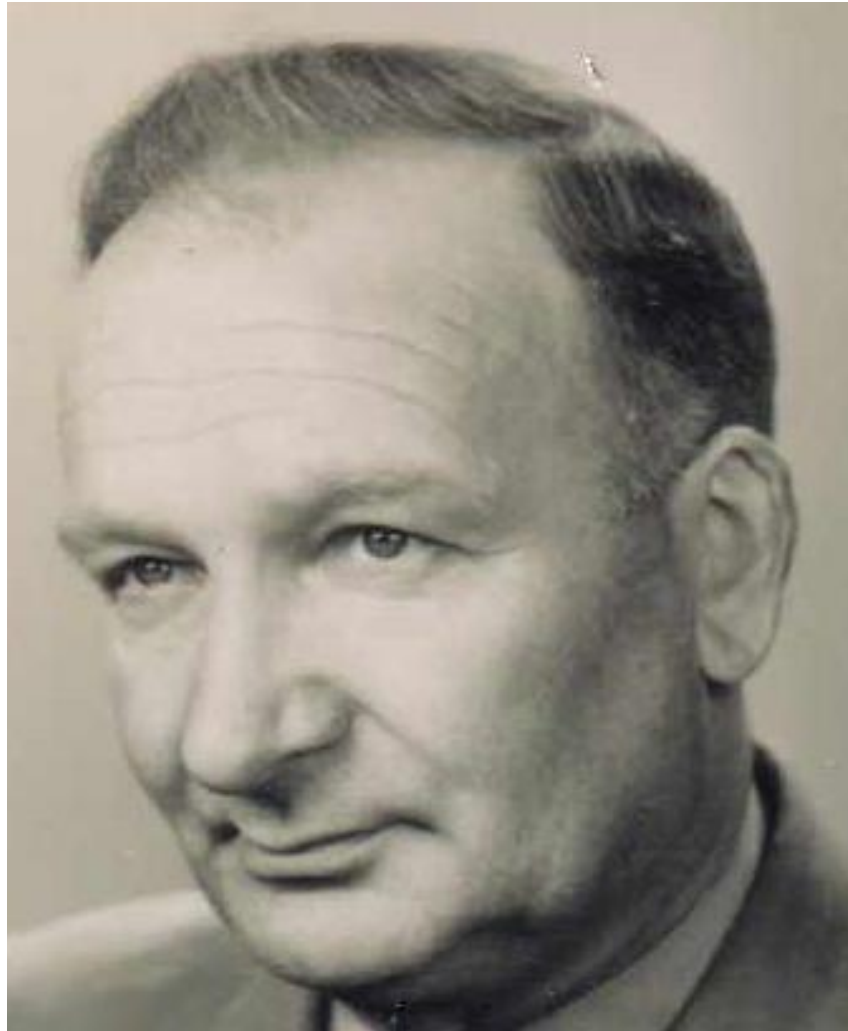

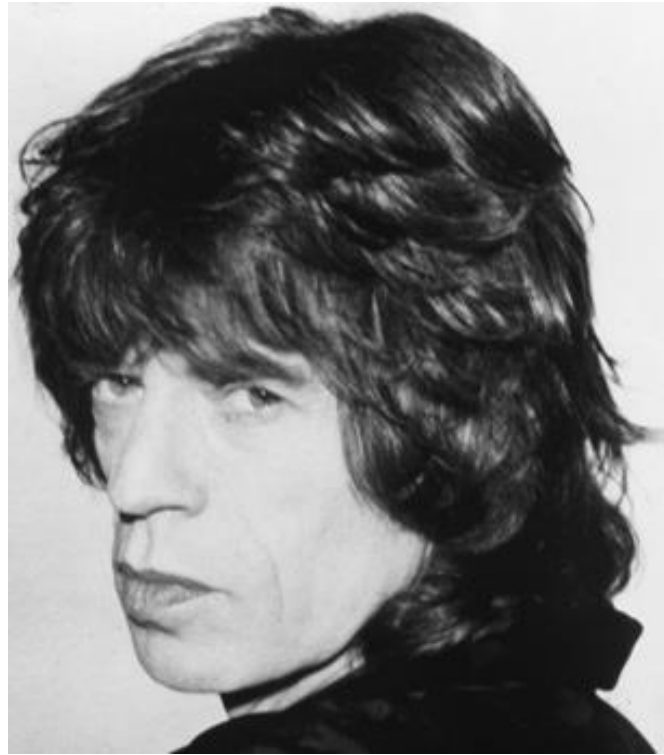

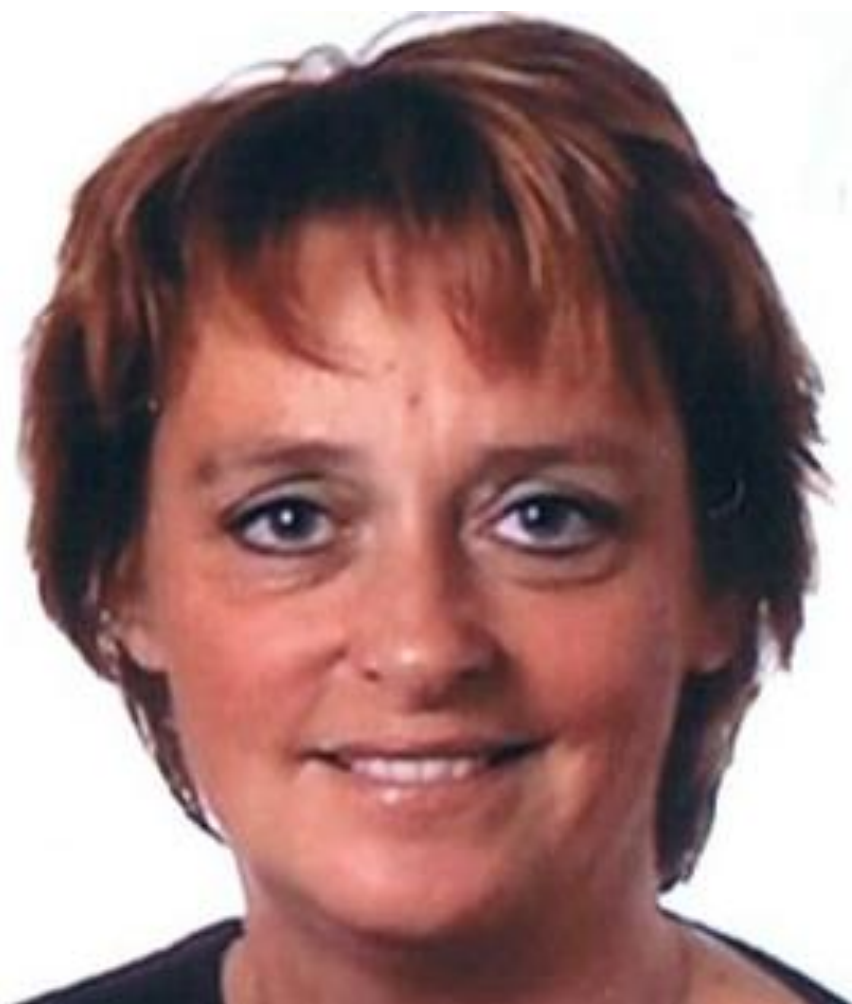

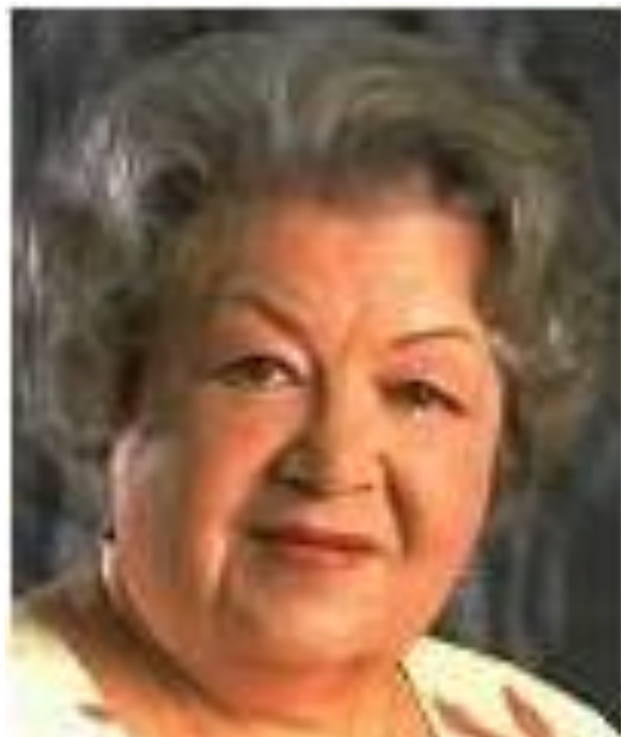

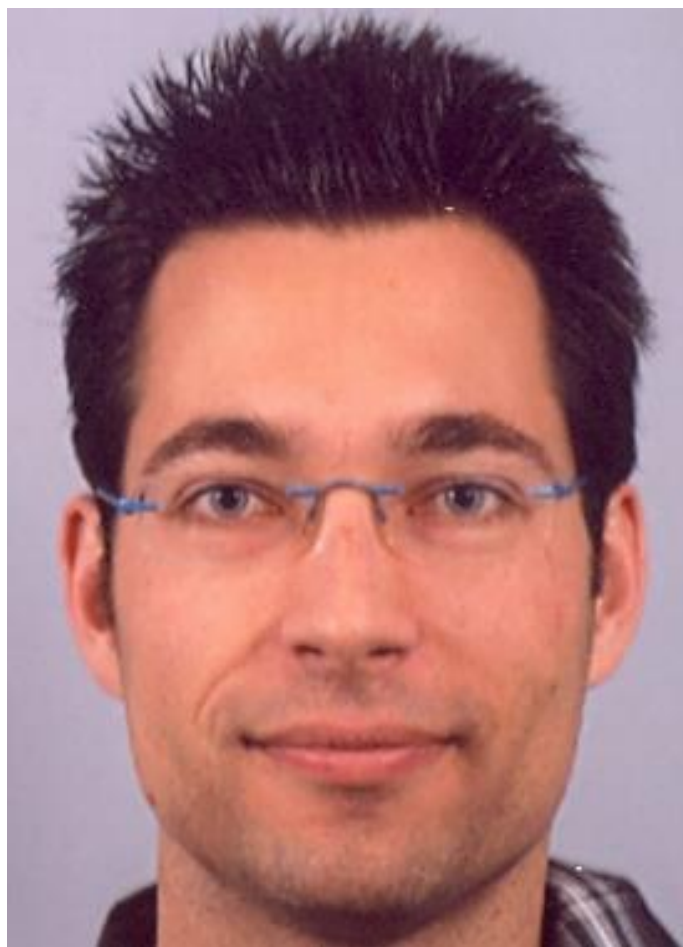

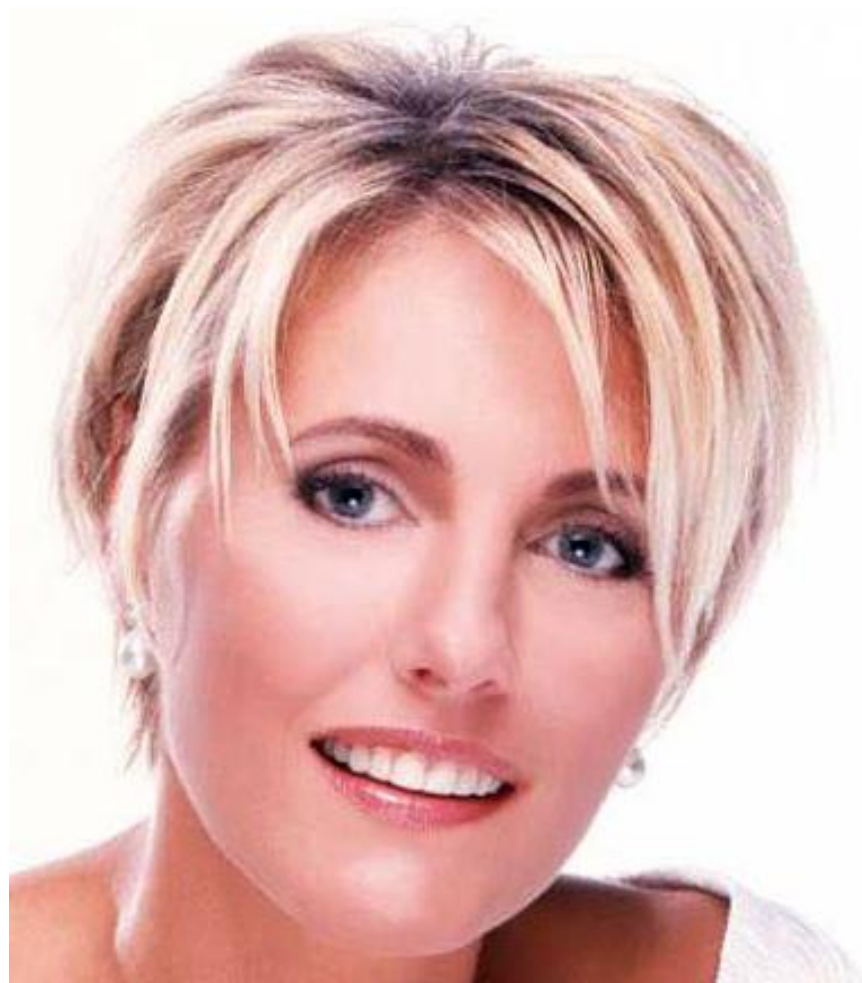

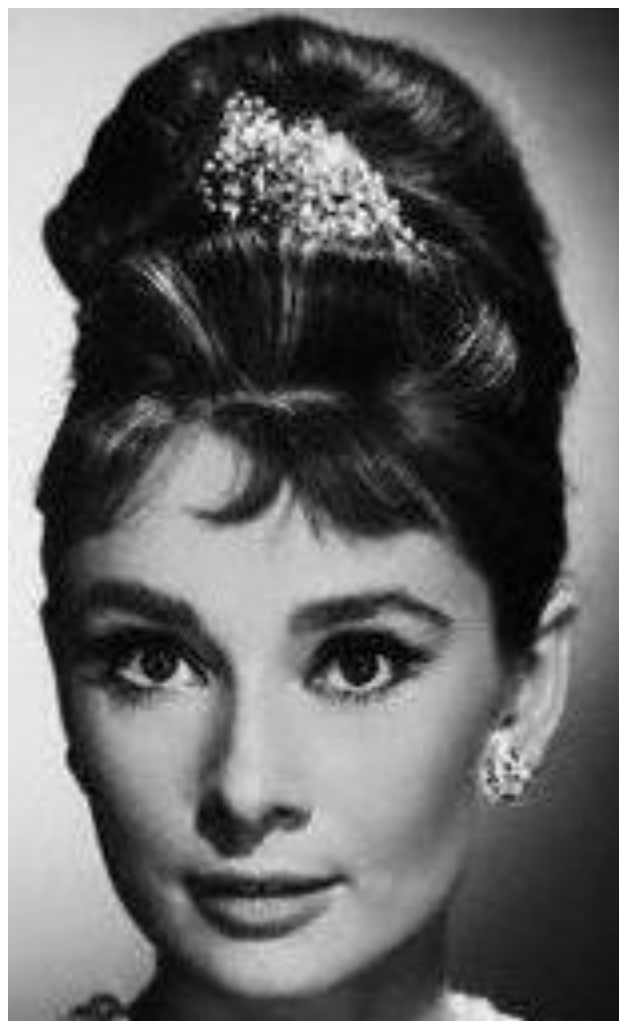

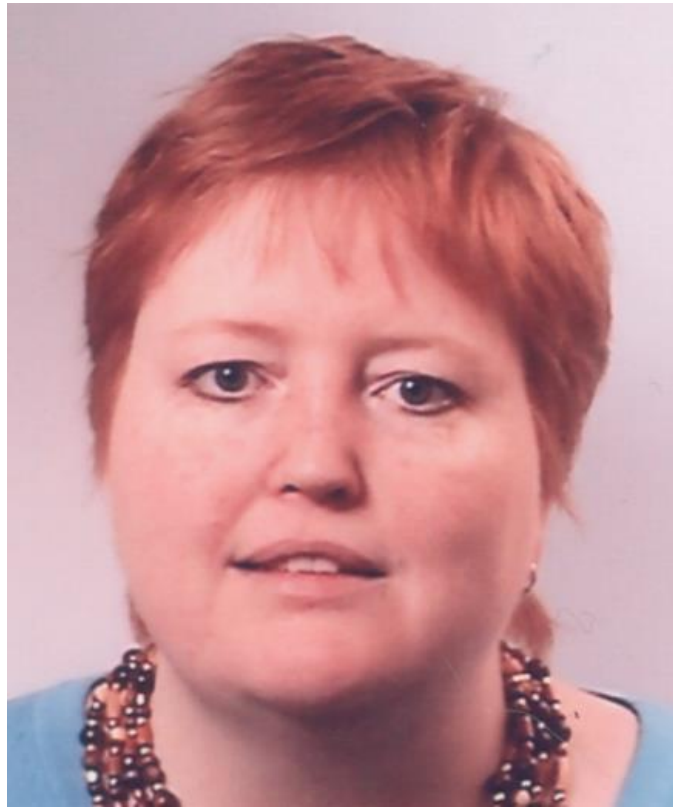

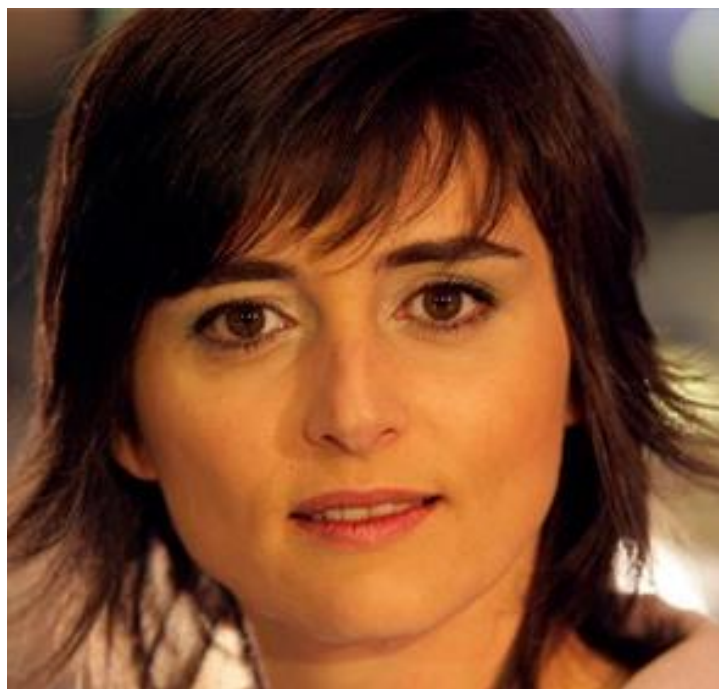

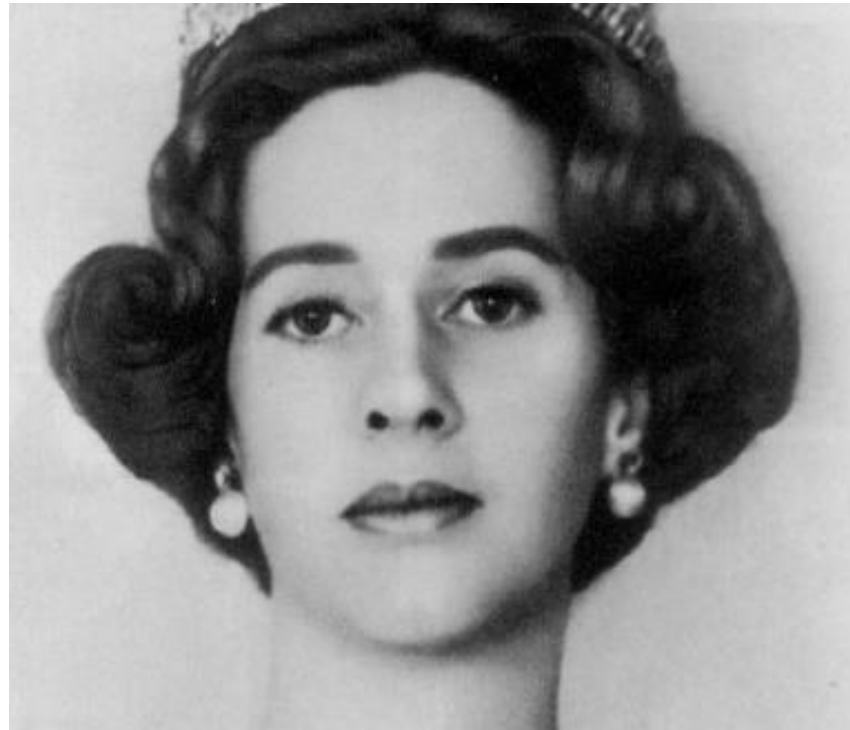

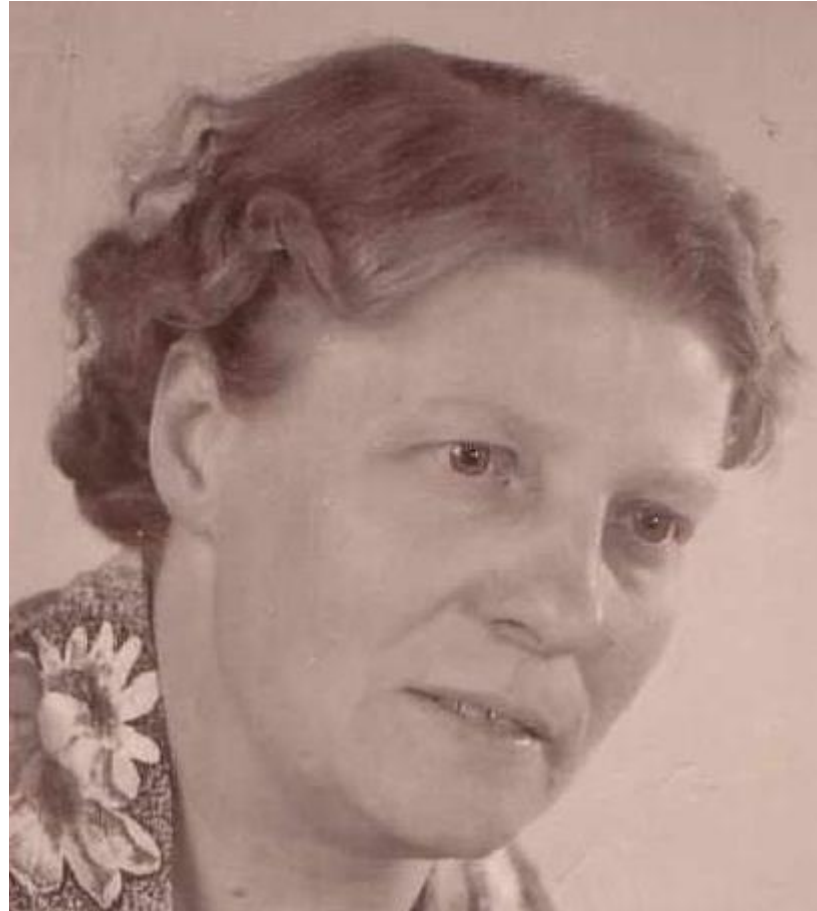

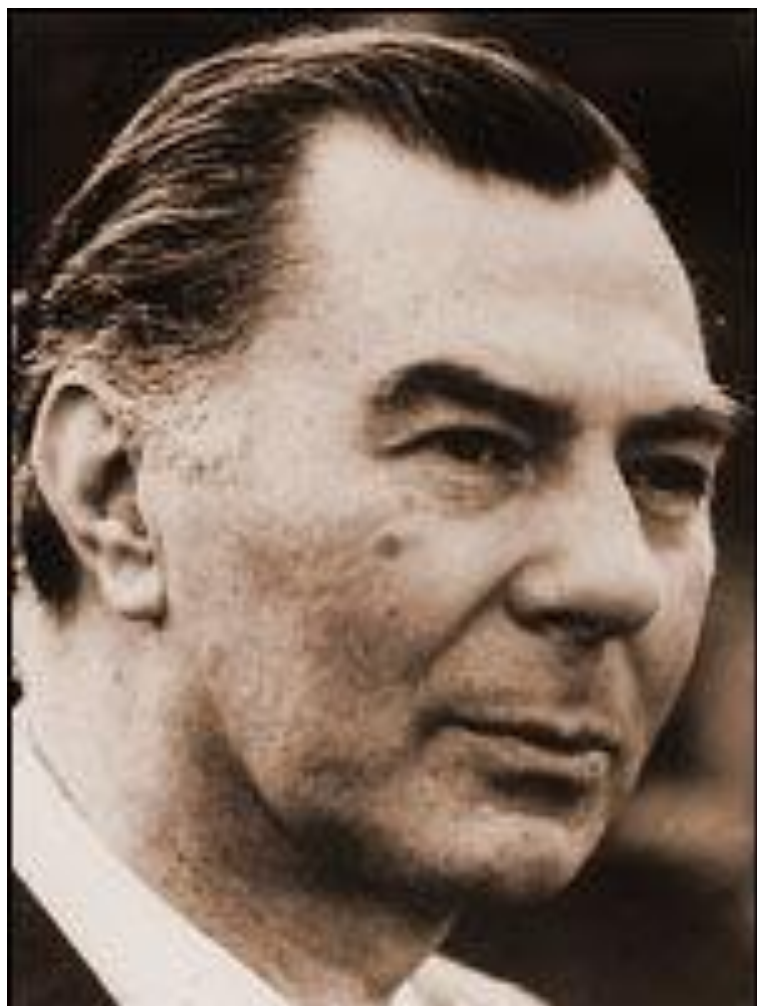

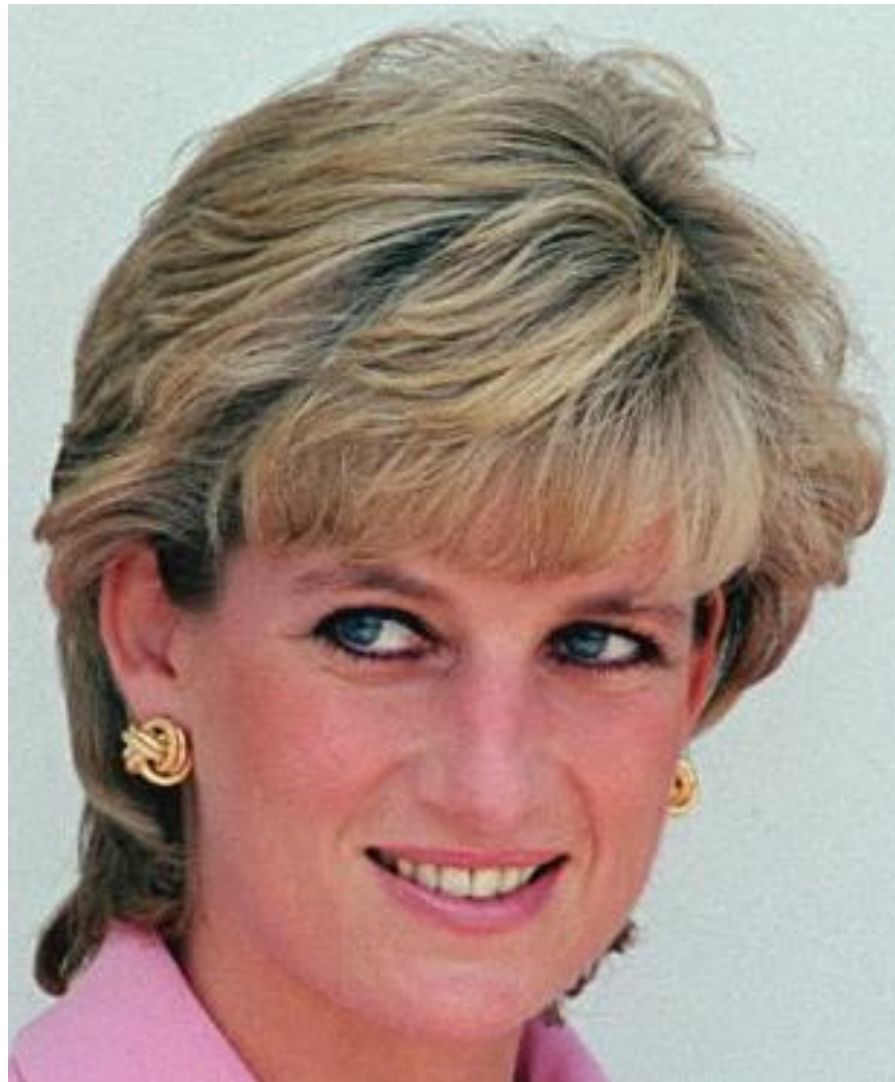

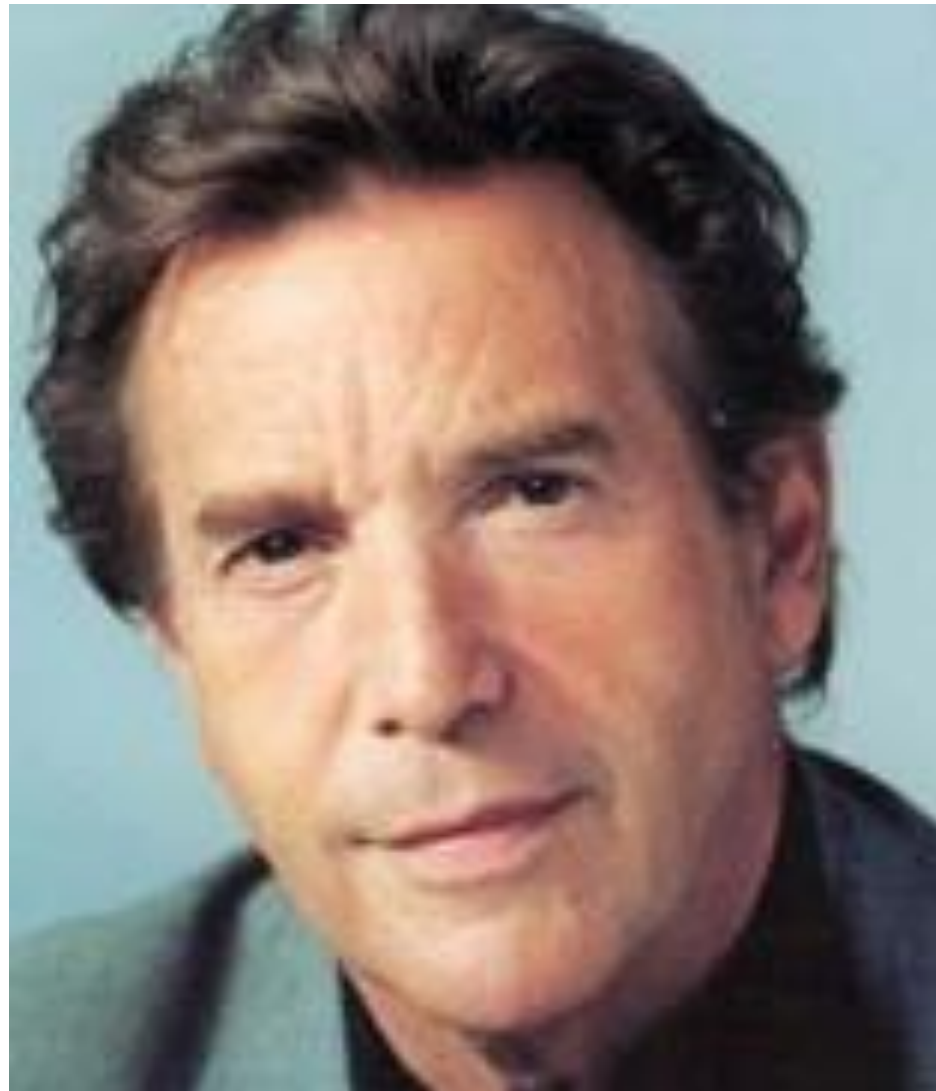

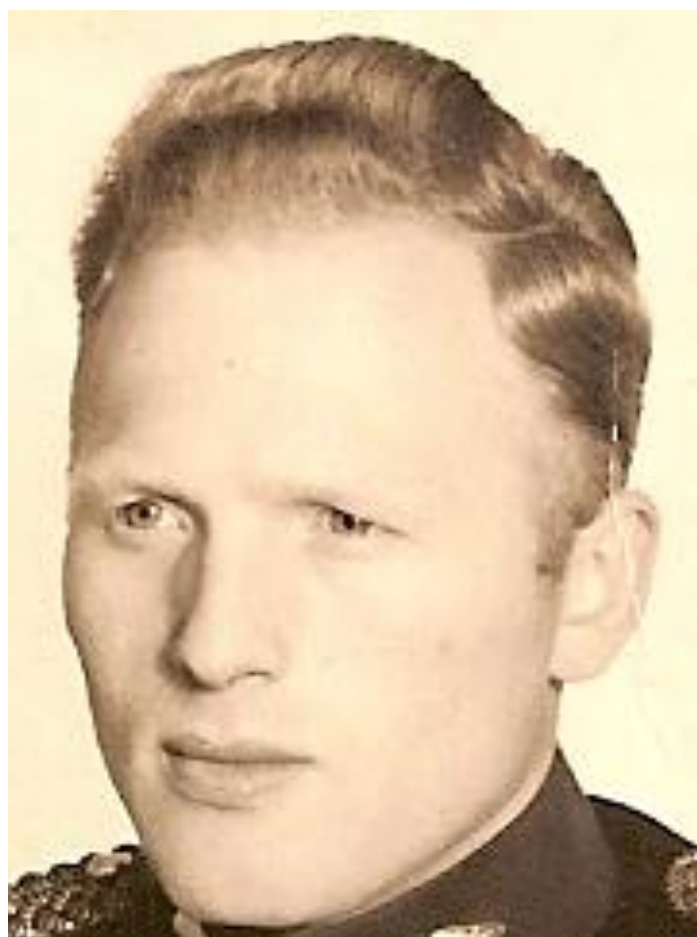

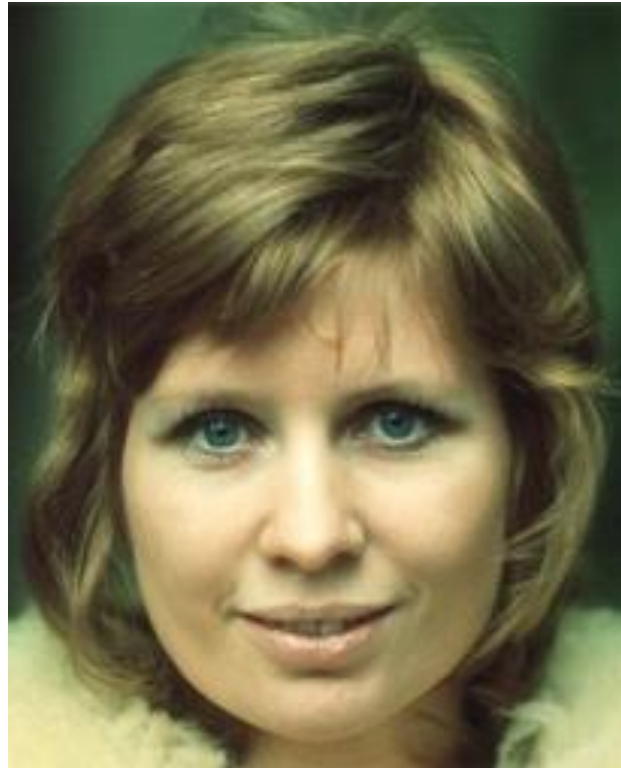

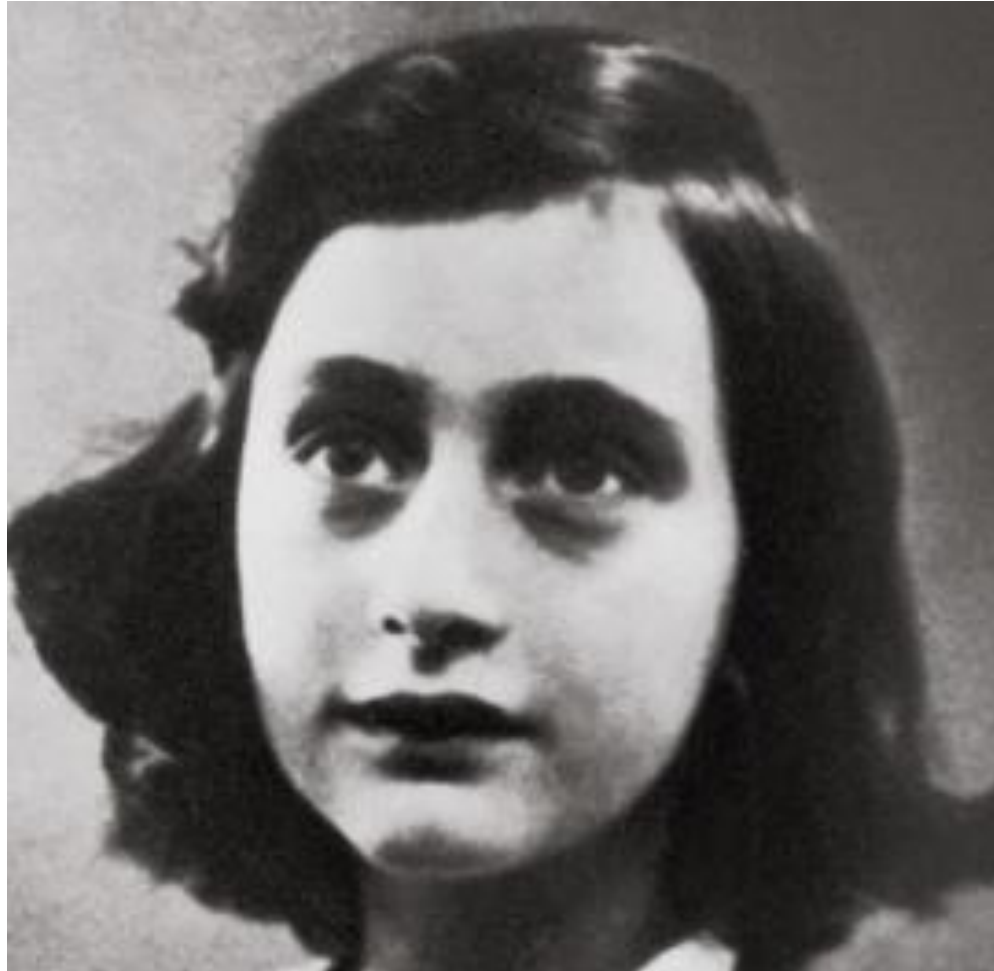

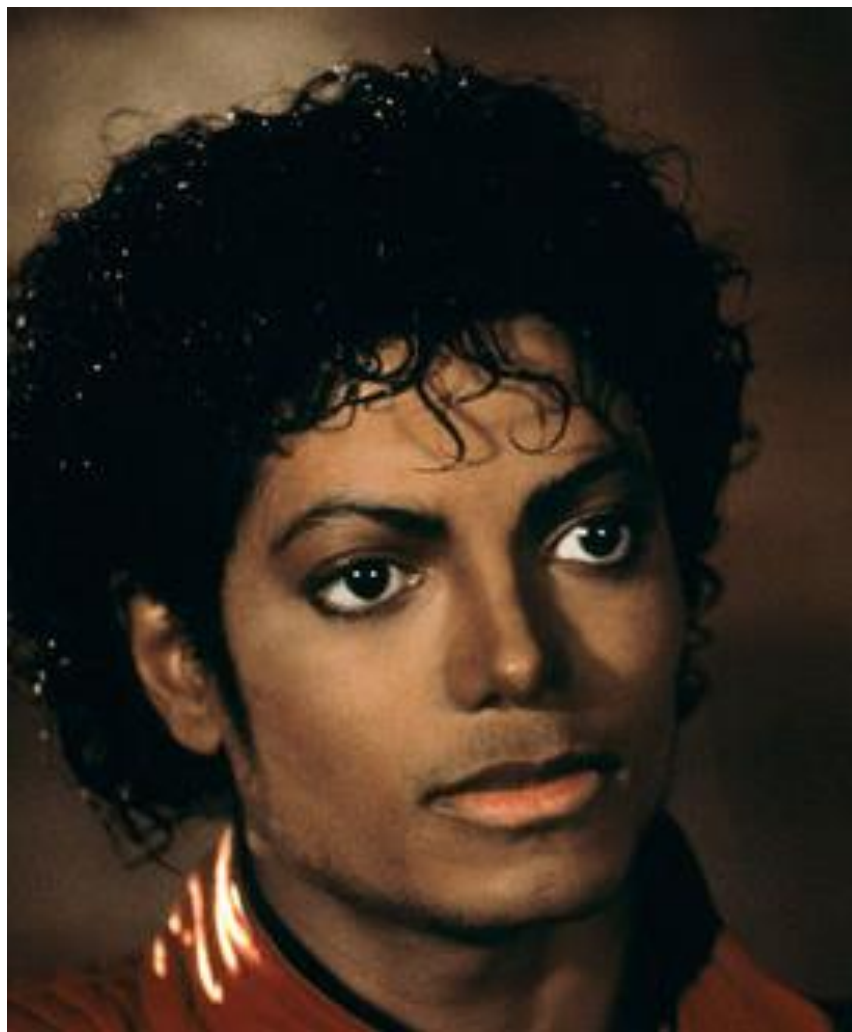

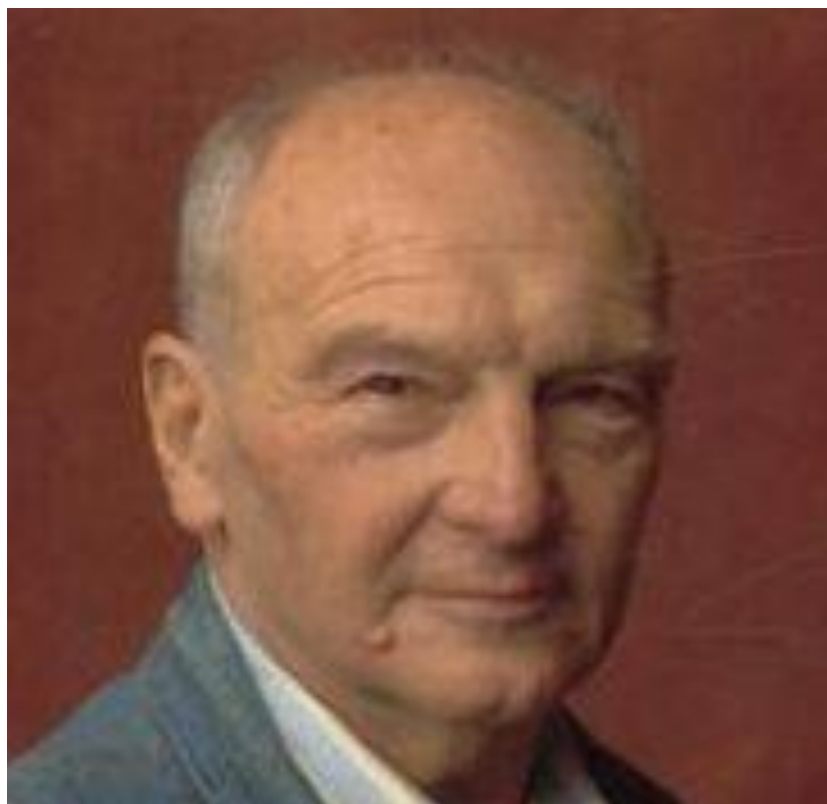

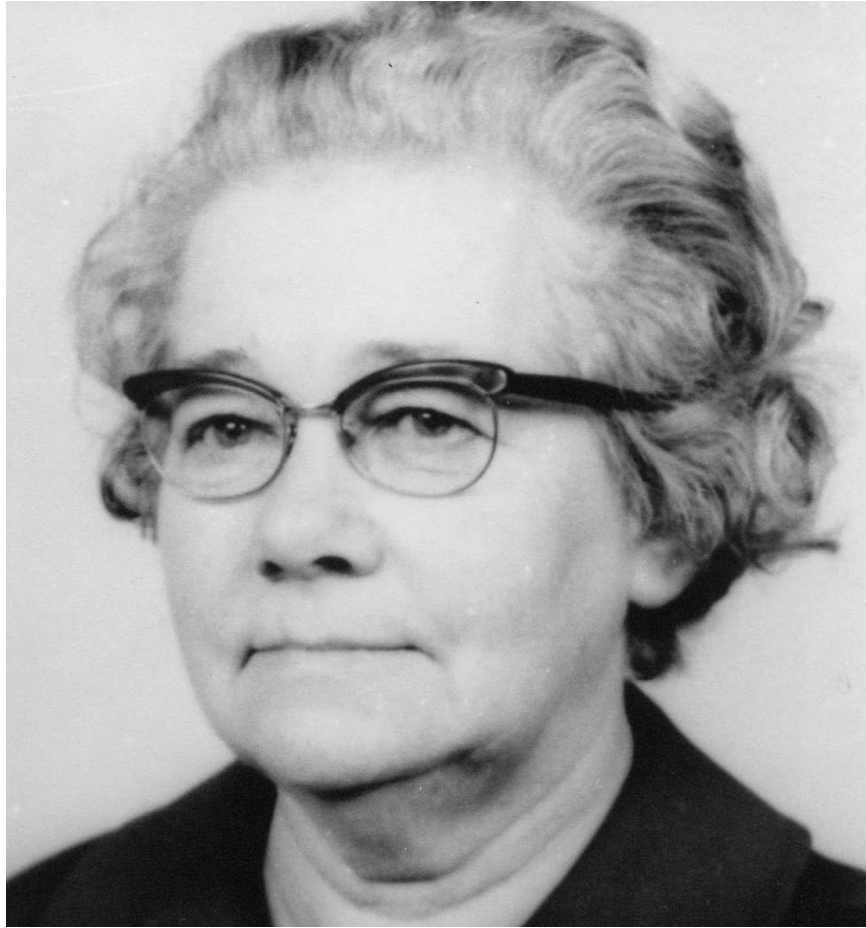

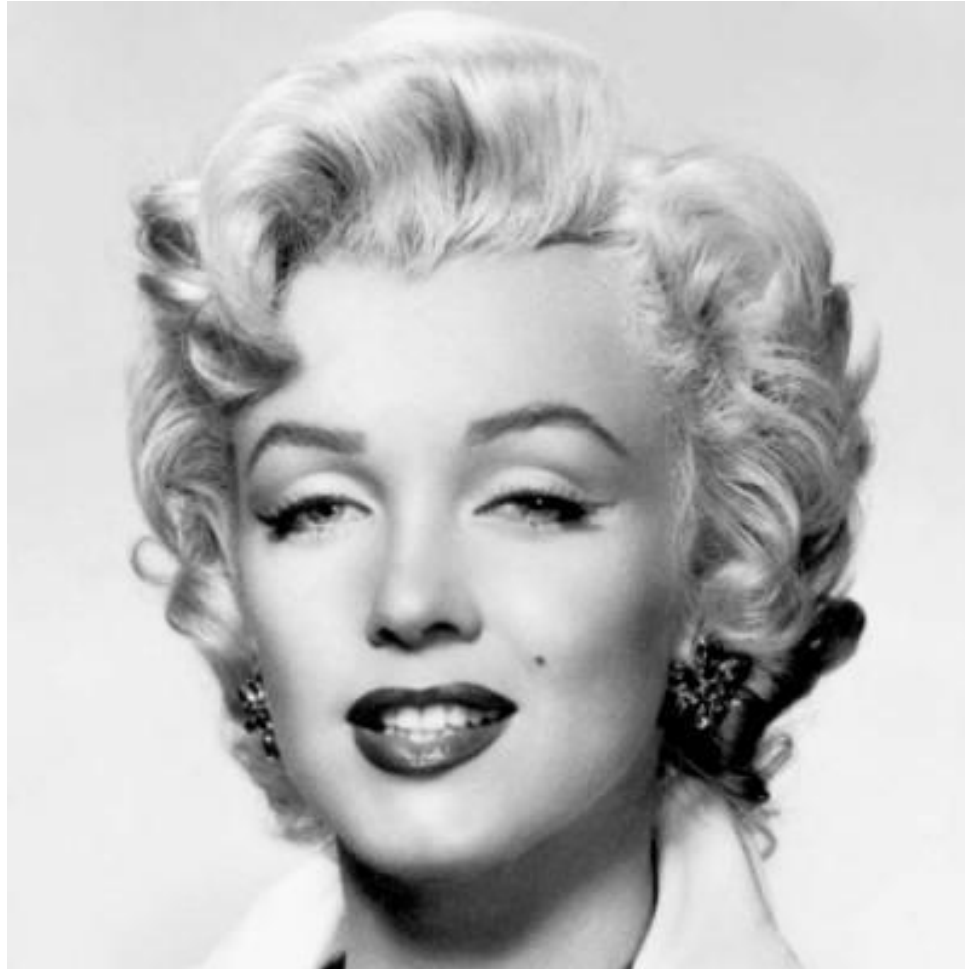

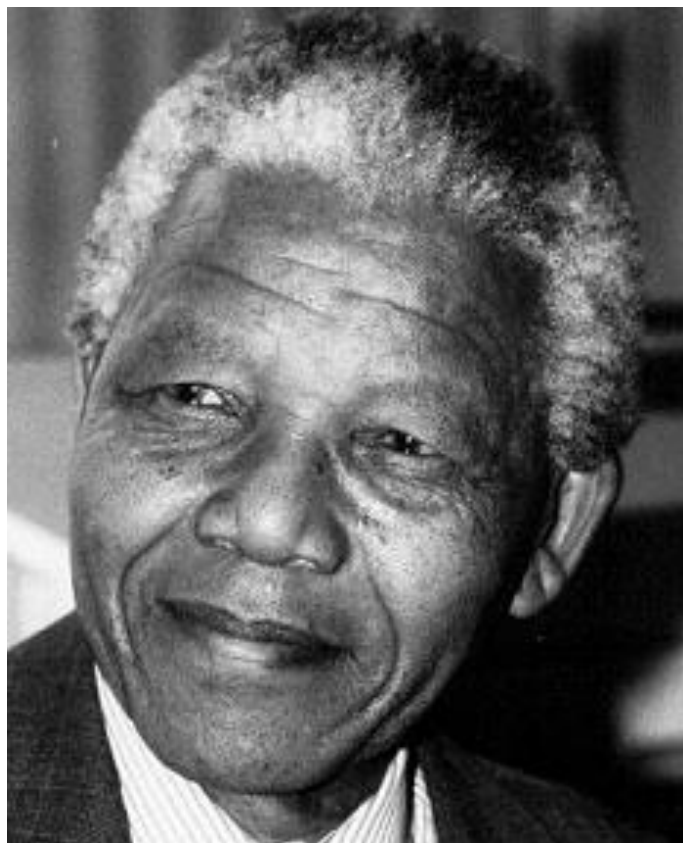

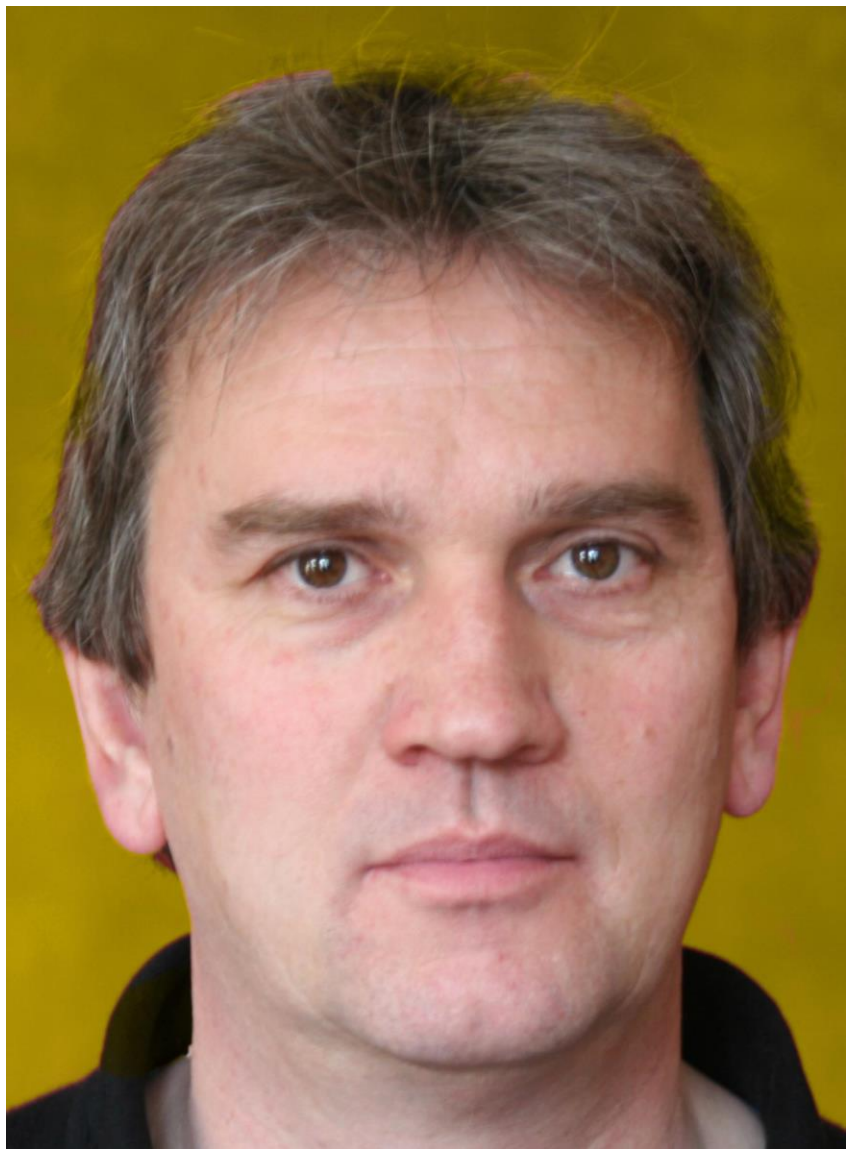

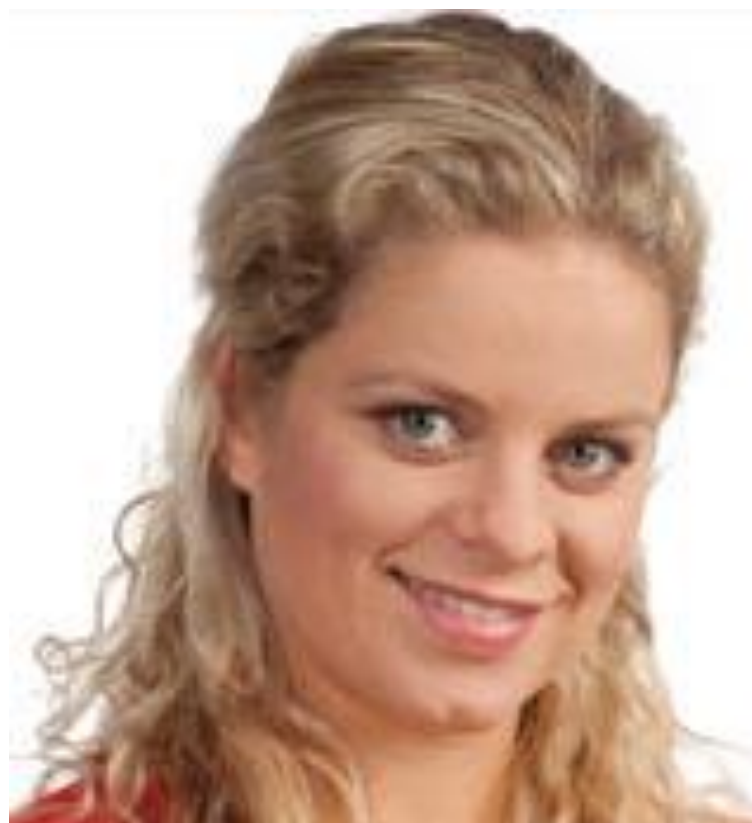

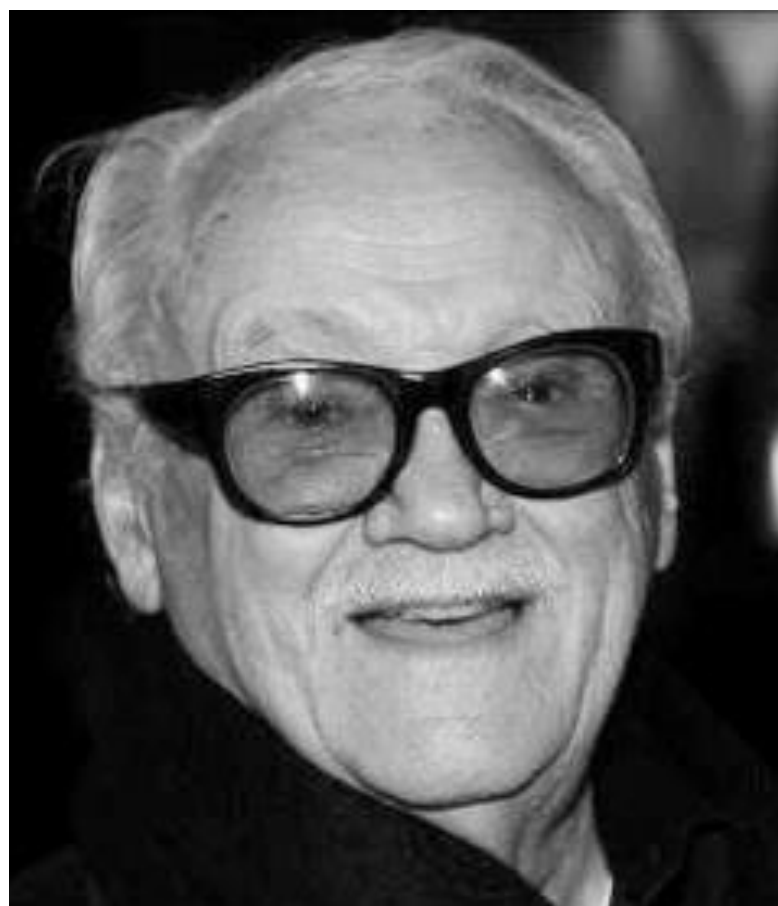

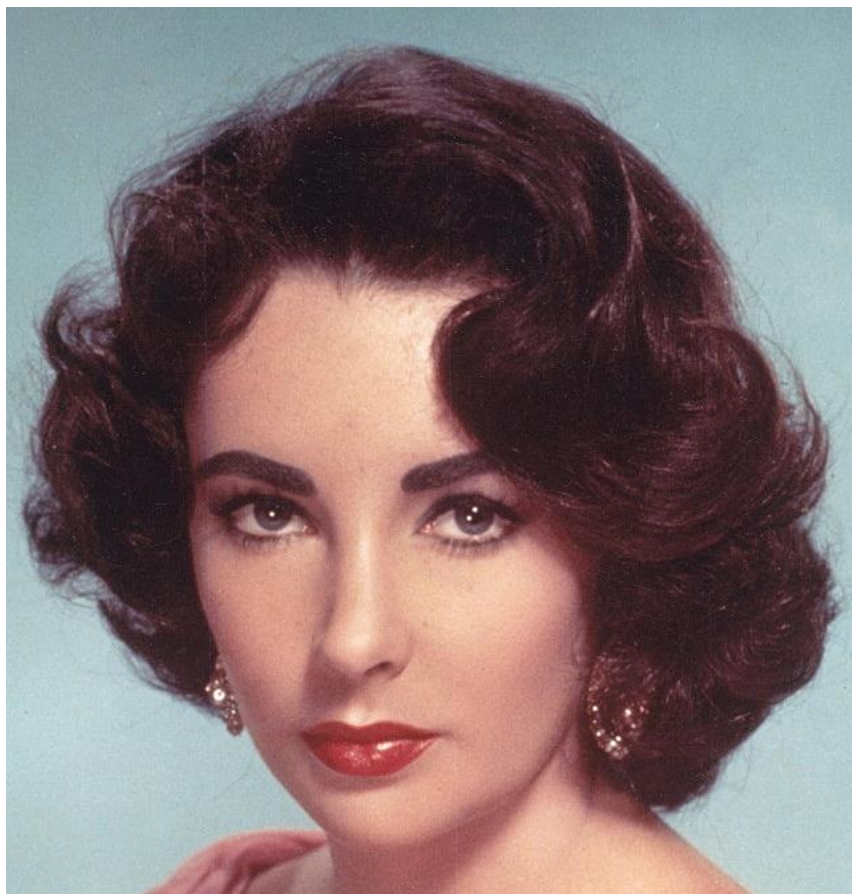

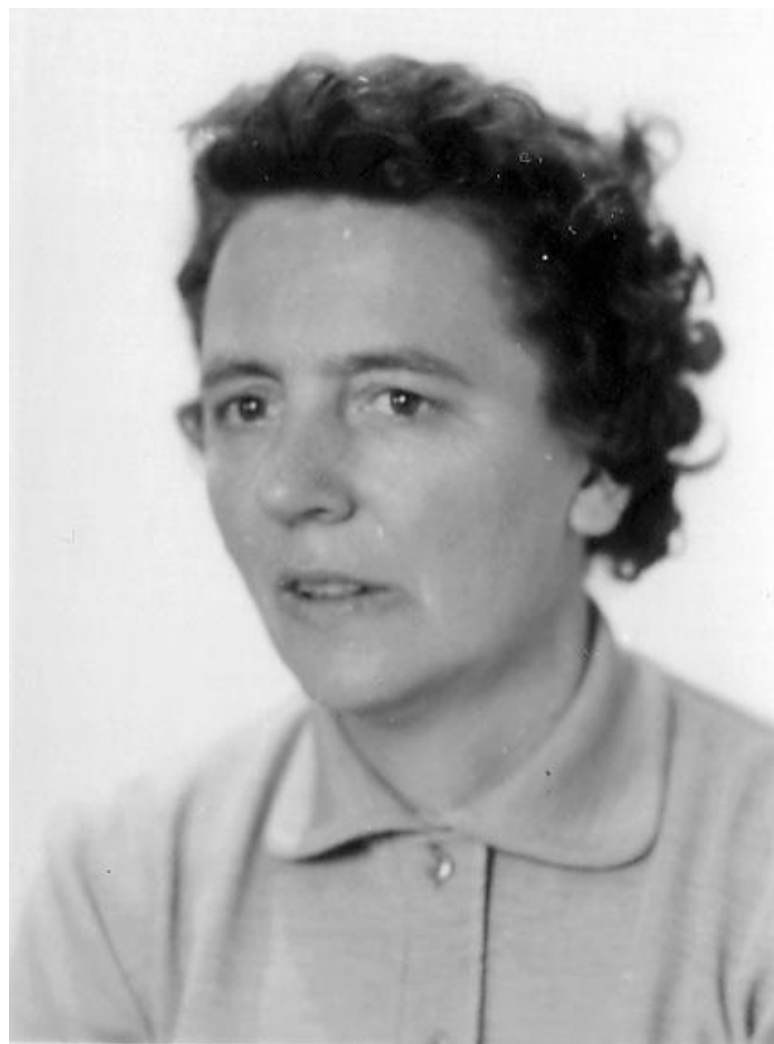

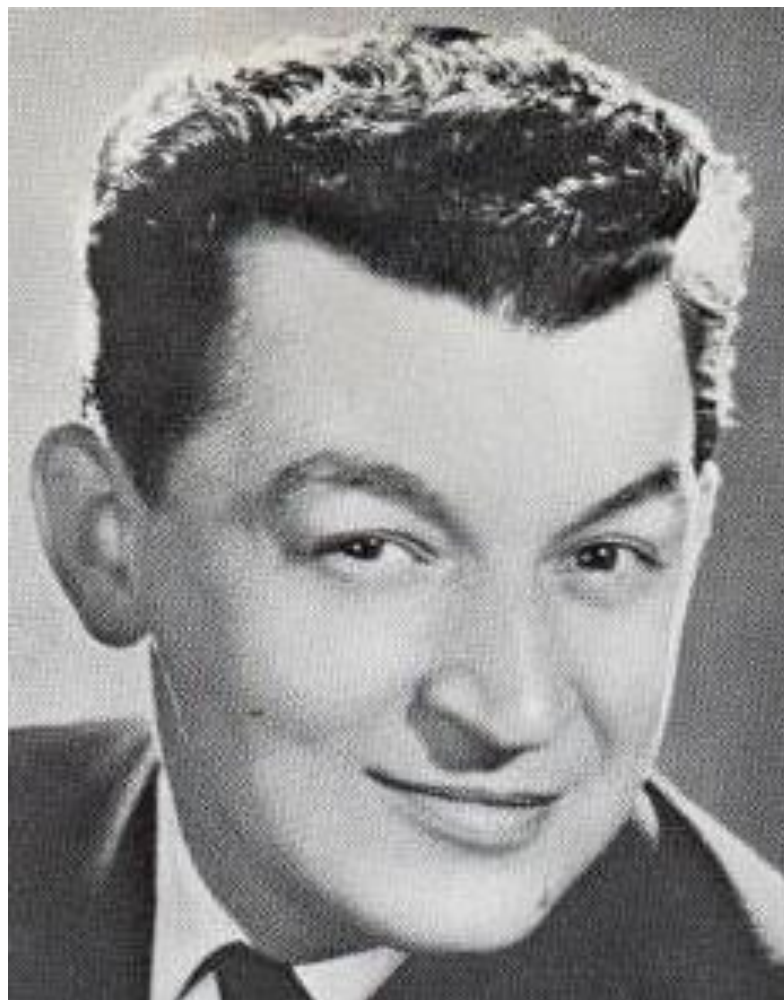

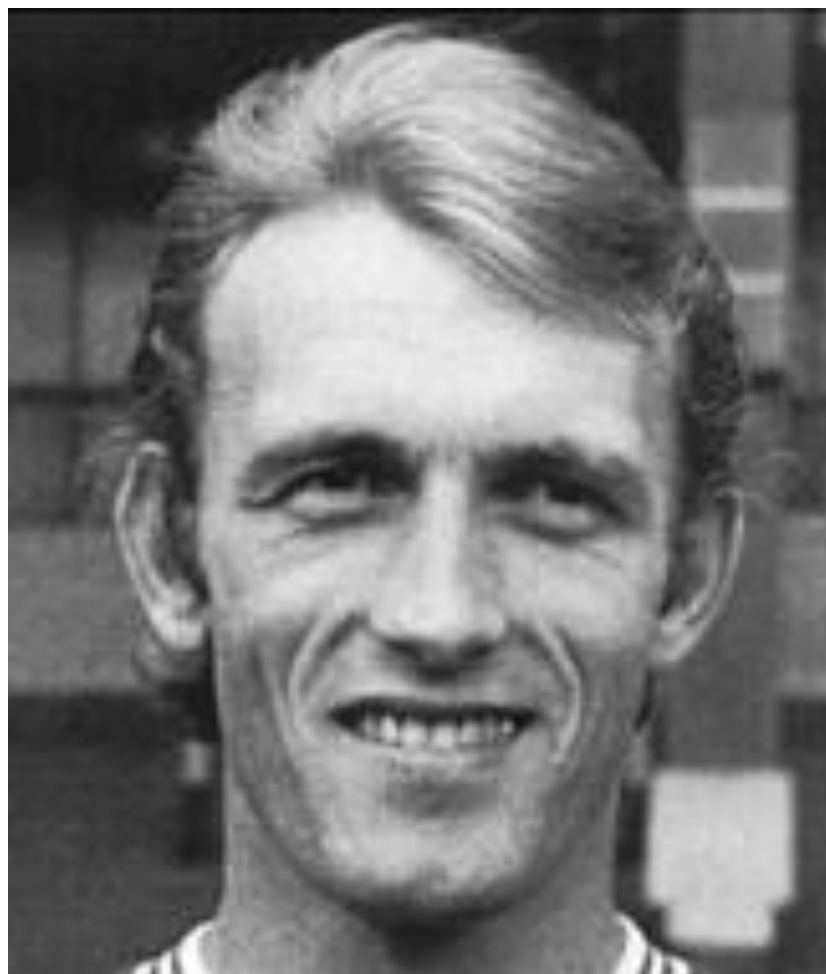

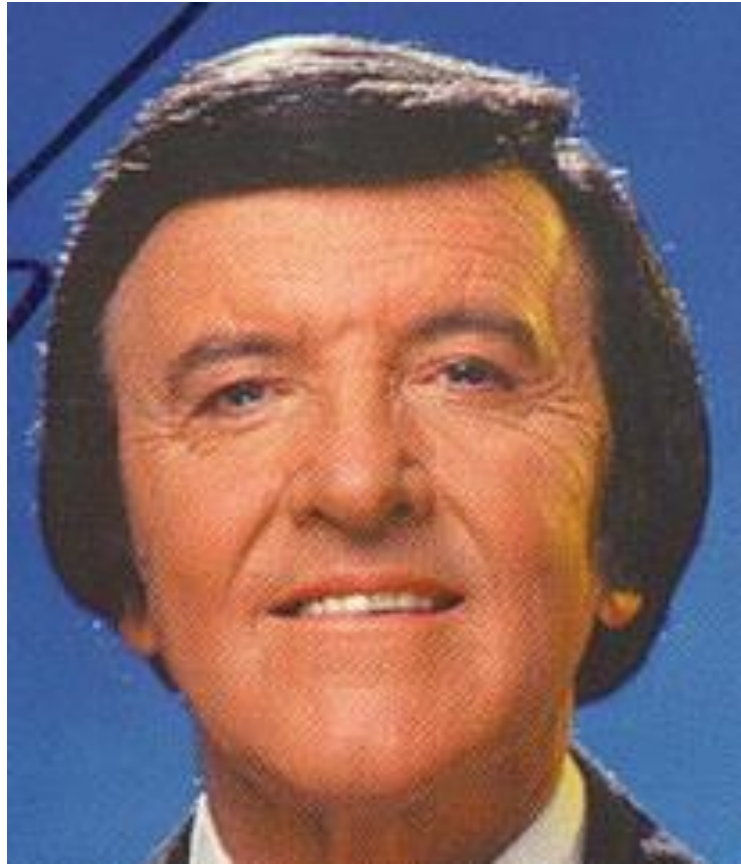

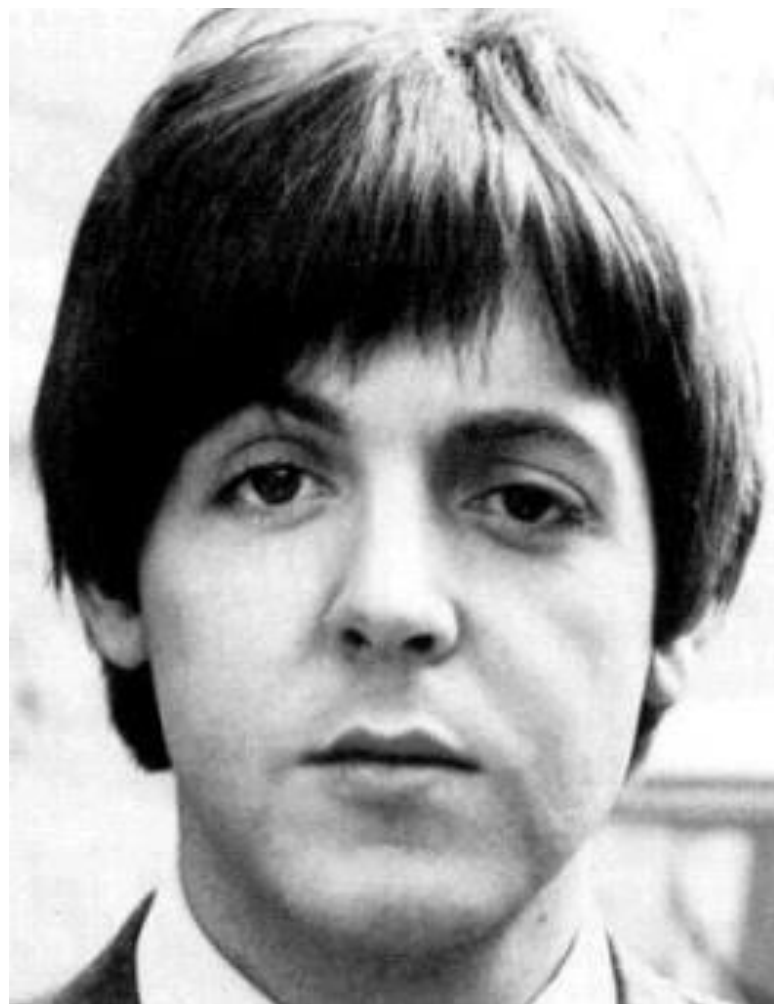

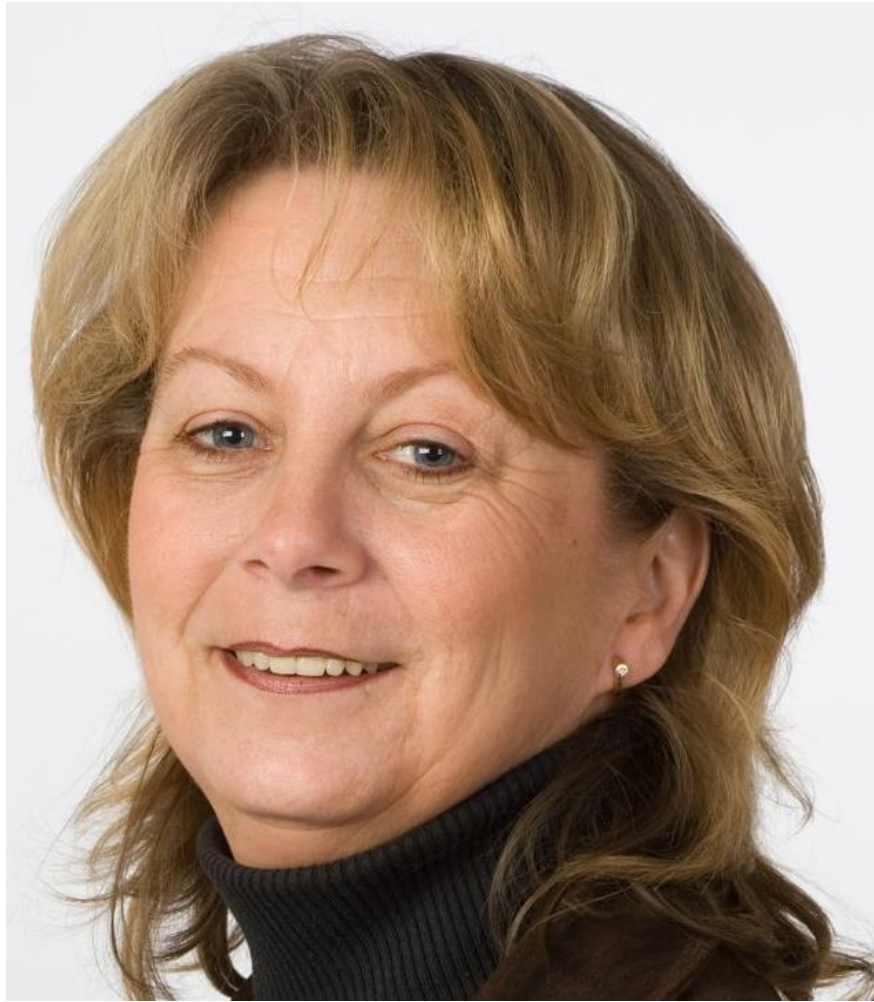

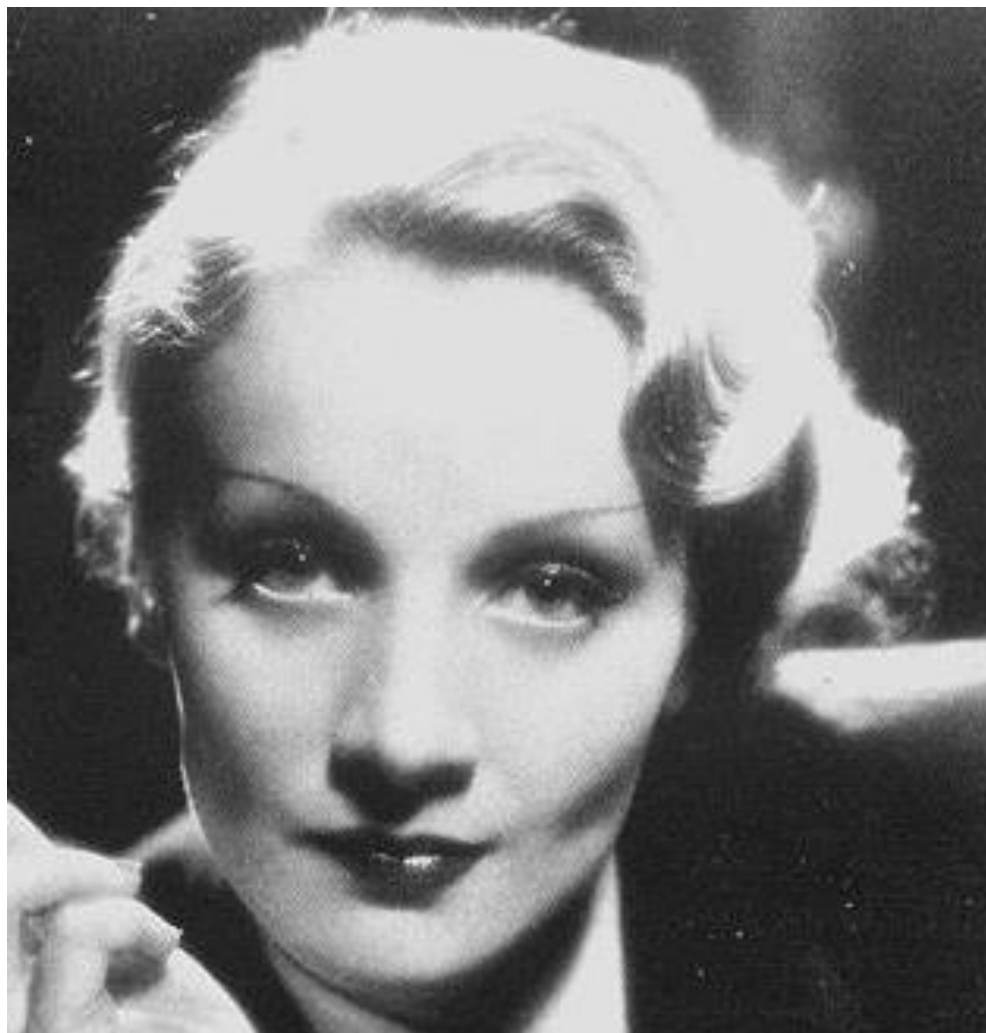

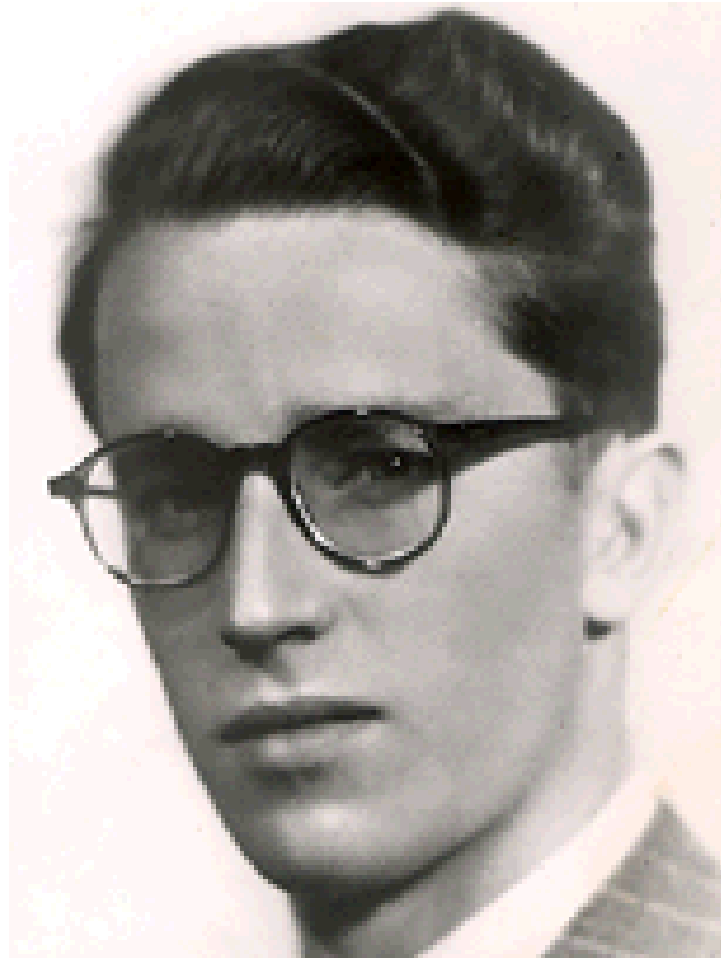

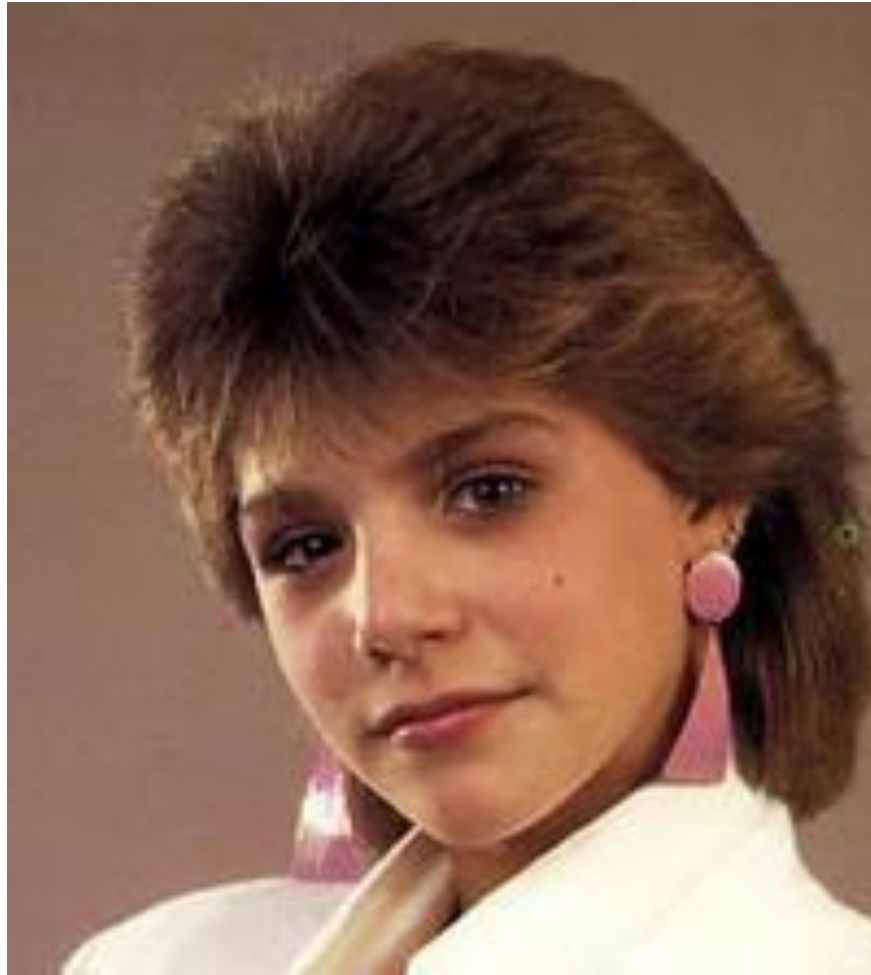

Supplement: Suppl. S1 — Stimuli of Experiment 3 - block 1 (familiarity detection). [file mmc2.pdf]
