# Supplementary material for: Face shape and face identity processing in behavioral variant fronto-temporal dementia: A specific deficit for familiarity and name recognition of famous faces
Source: Neuroimage Clin. 2016 Mar 10;11:368–77. doi: 10.1016/j.nicl.2016.03.001 (PMC4893012; doi:10.1016/j.nicl.2016.03.001)
Supplement: Suppl. S2 — Stimuli of Experiment 3 - block 2 (face-name matching). [file mmc3.pdf]

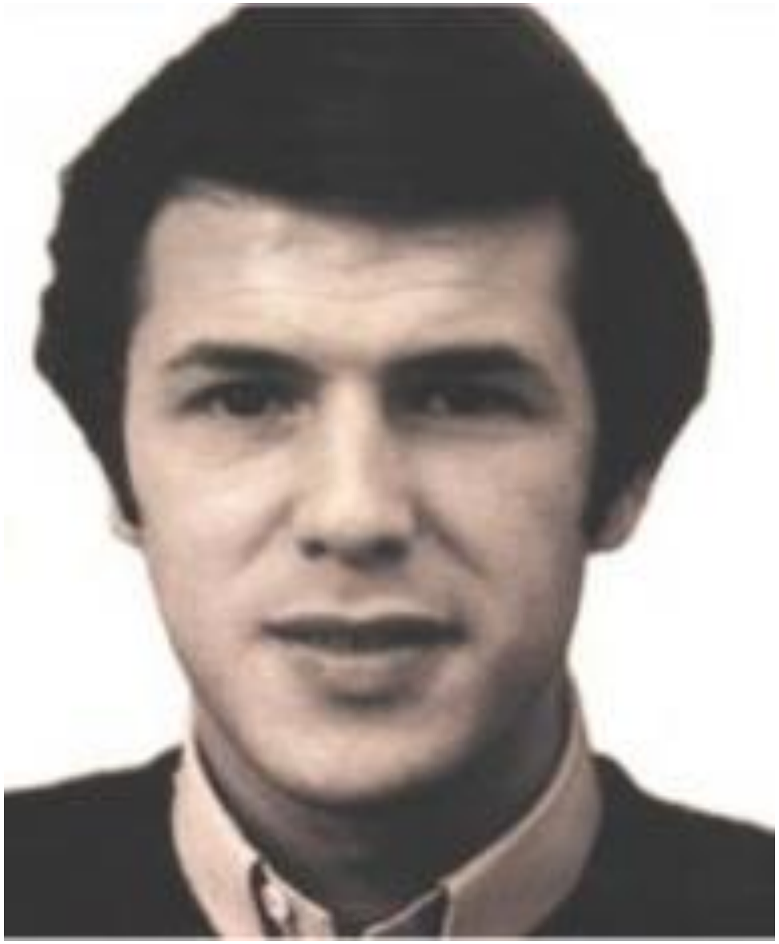

1. LEO MARTIN 2. THEO LEFEVRE 3. ADAMO

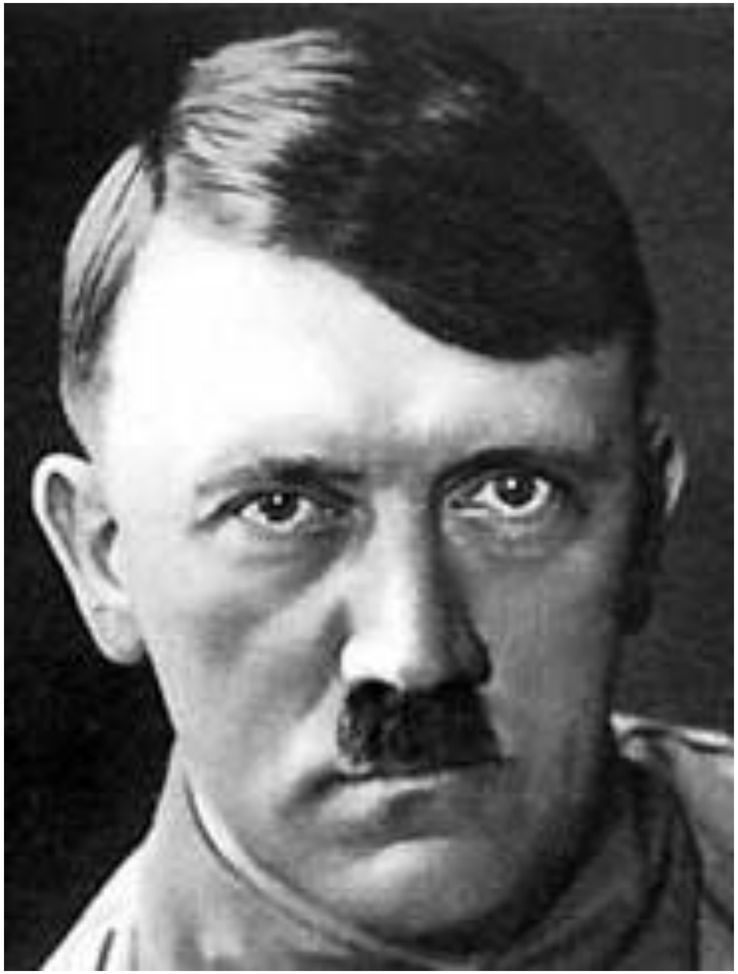

1. VLADIMIR LENIN 2. ADOLF HITLER 3. STAN LAUREL

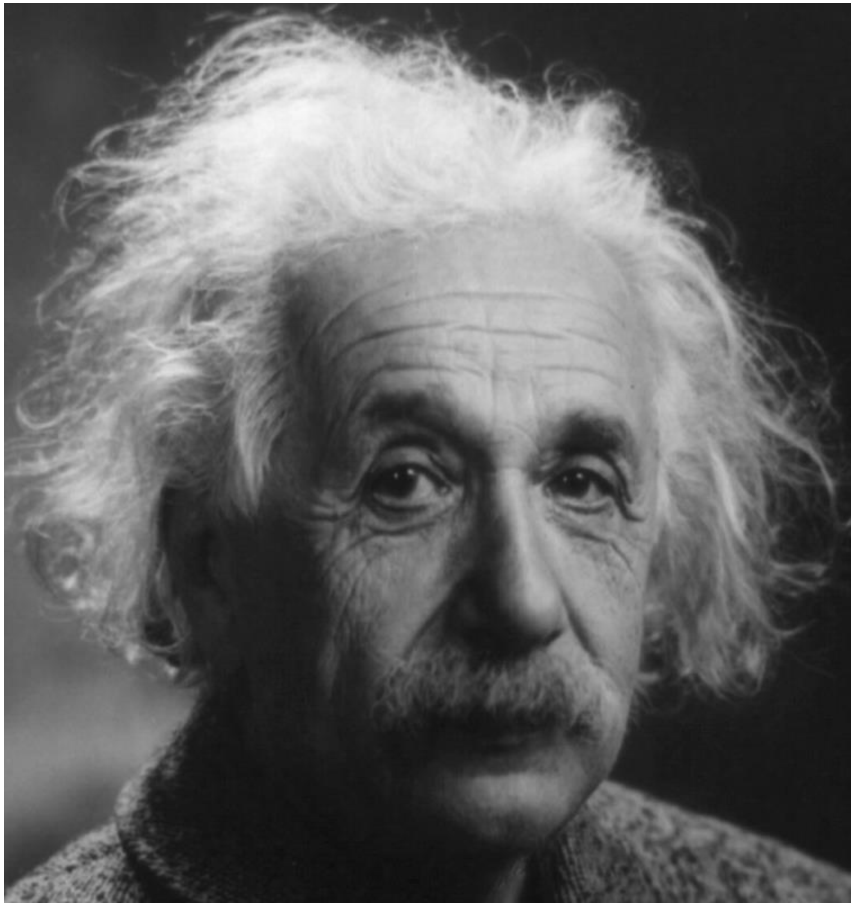

1. ALBERT EINSTEIN 2. JOHN WAYNE 3. JOHNNY CASH

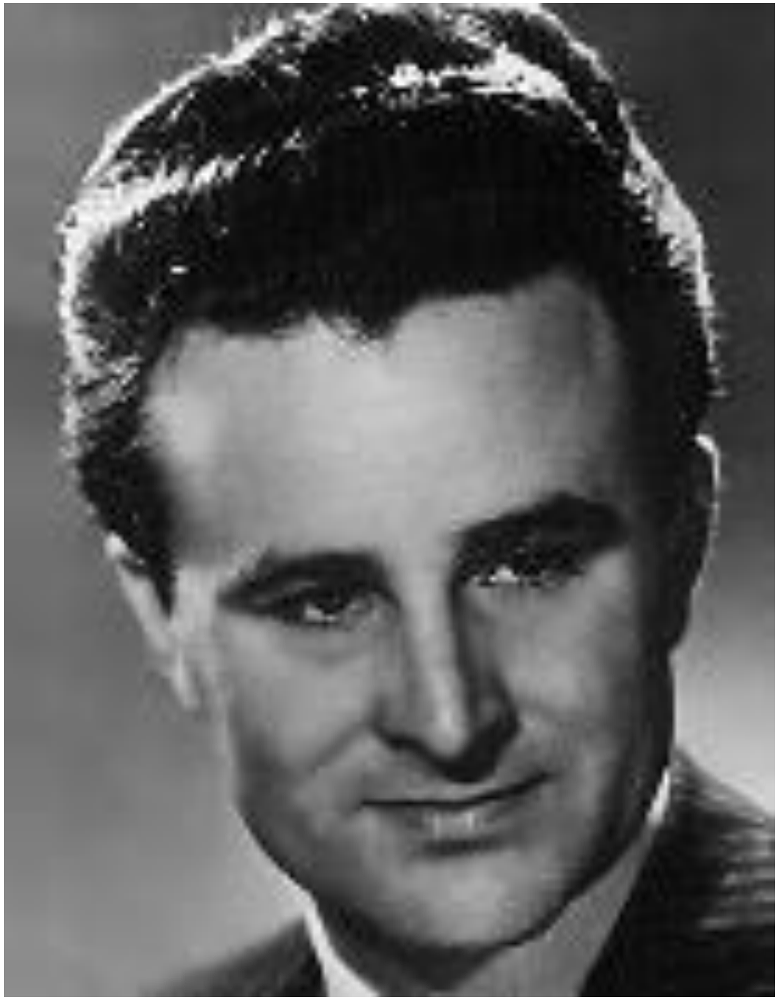

1. LUDO MARTENS 2. SENNE ROUFFAER 3. BOBBEJAAN SCHOEPEN

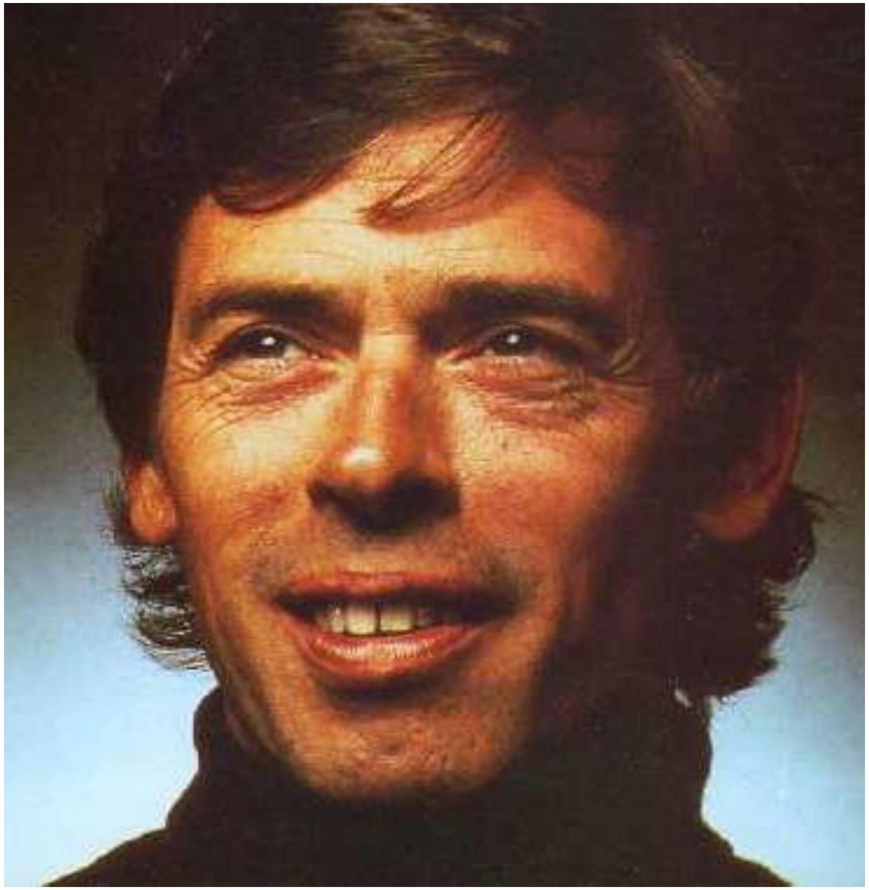

1. LUC APPERMONT 2. JACQUES BREL 3. NAND BAERT

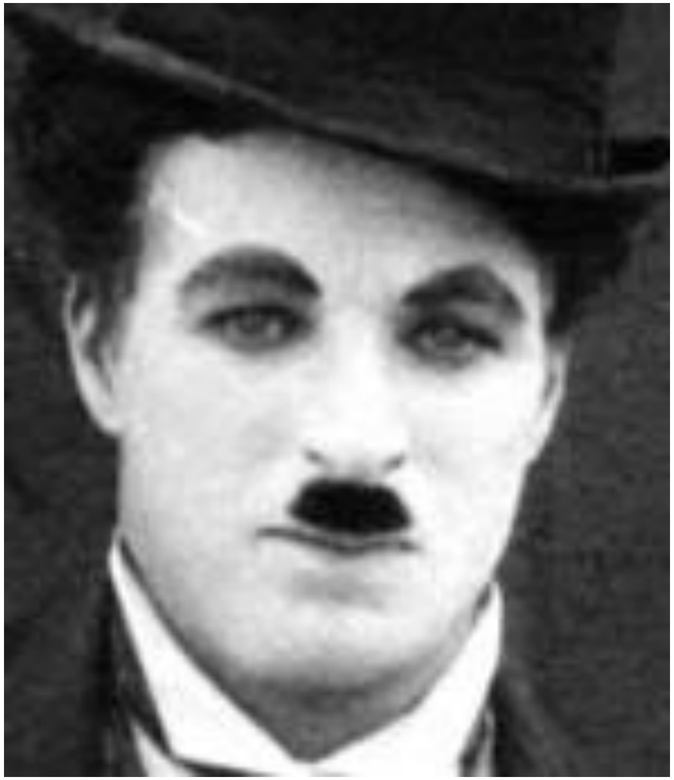

1. RONALD REAGAN 2. OLIVER HARDY 3. CHARLIE CHAPLIN

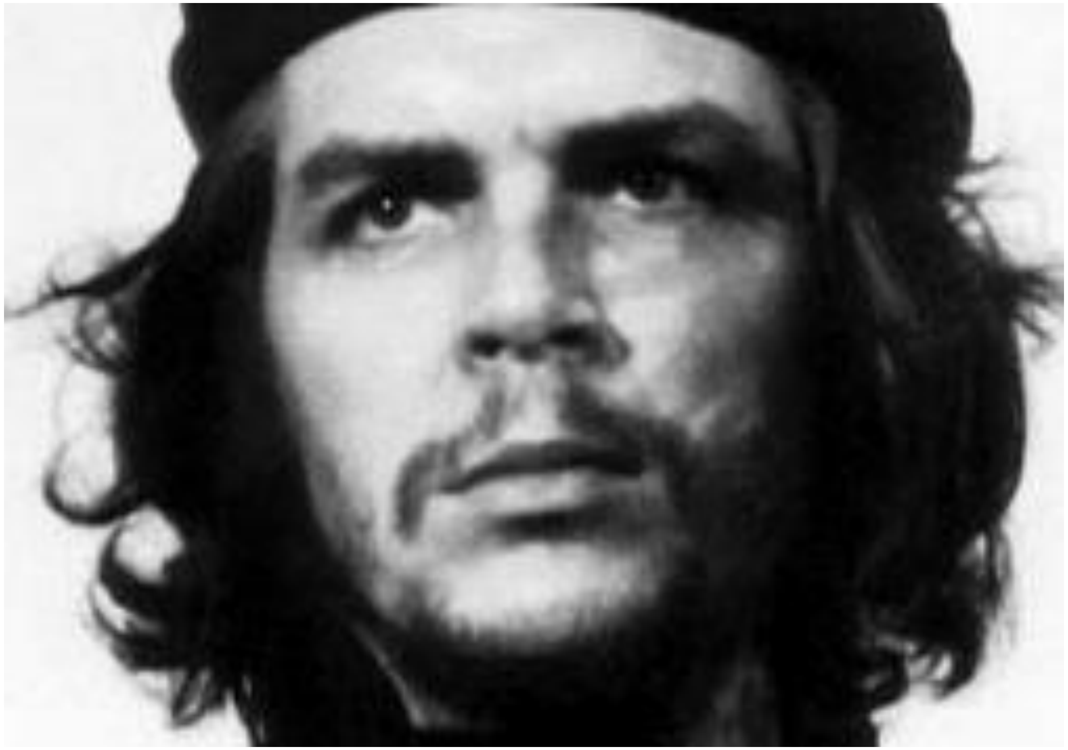

1. DIEGO MARADONA 2. CHE GUEVARA 3. SADAM HUSSEIN

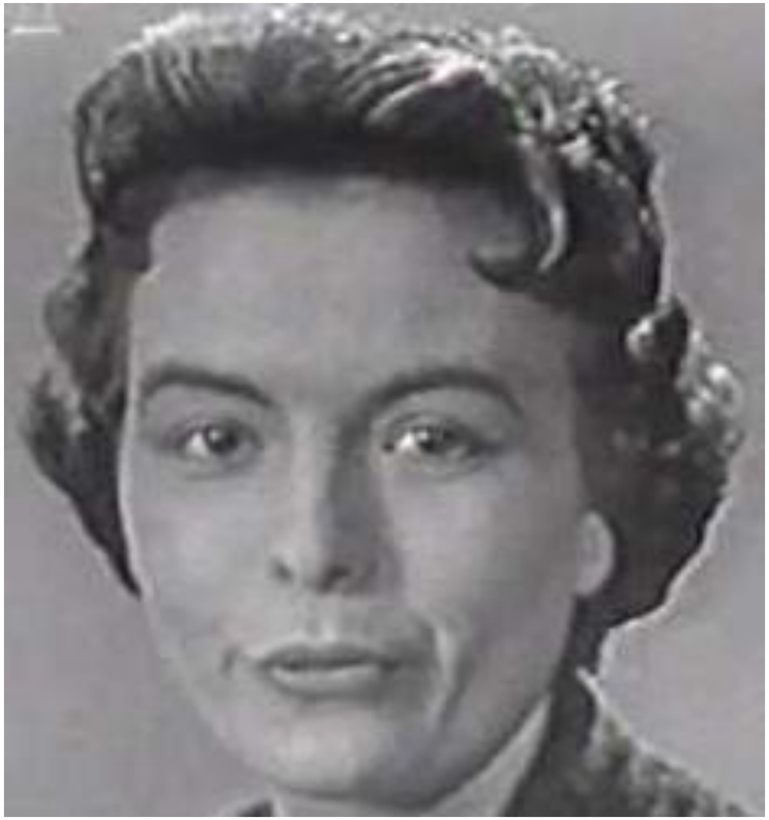

1. TANTE TERRY 2. LA ESTERELLA 3. CHRIS LOMME

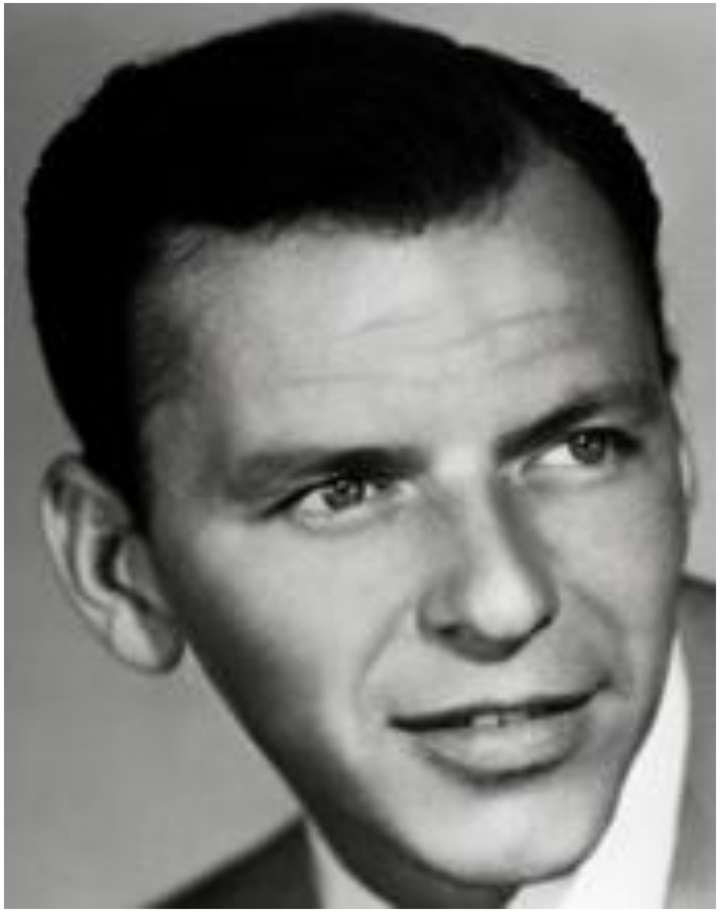

1. CLARK GABLE 2. LUCIANO PAVAROTTI 3. FRANK SINATRA

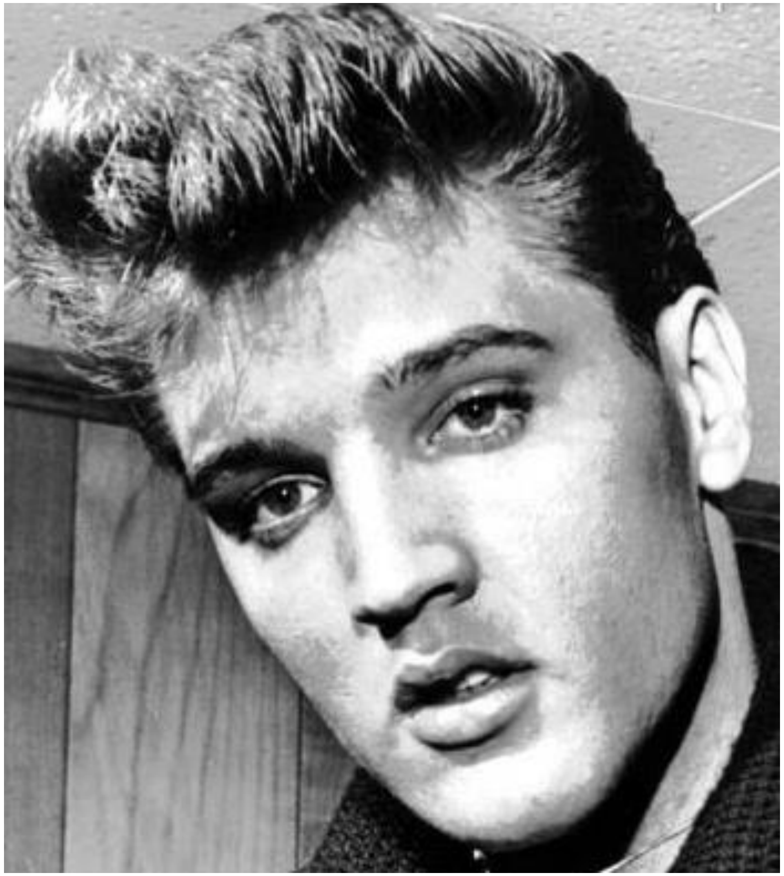

1. GEORGE BUSH 2. ELVIS PRESLEY 3. JOHN KENNEDY

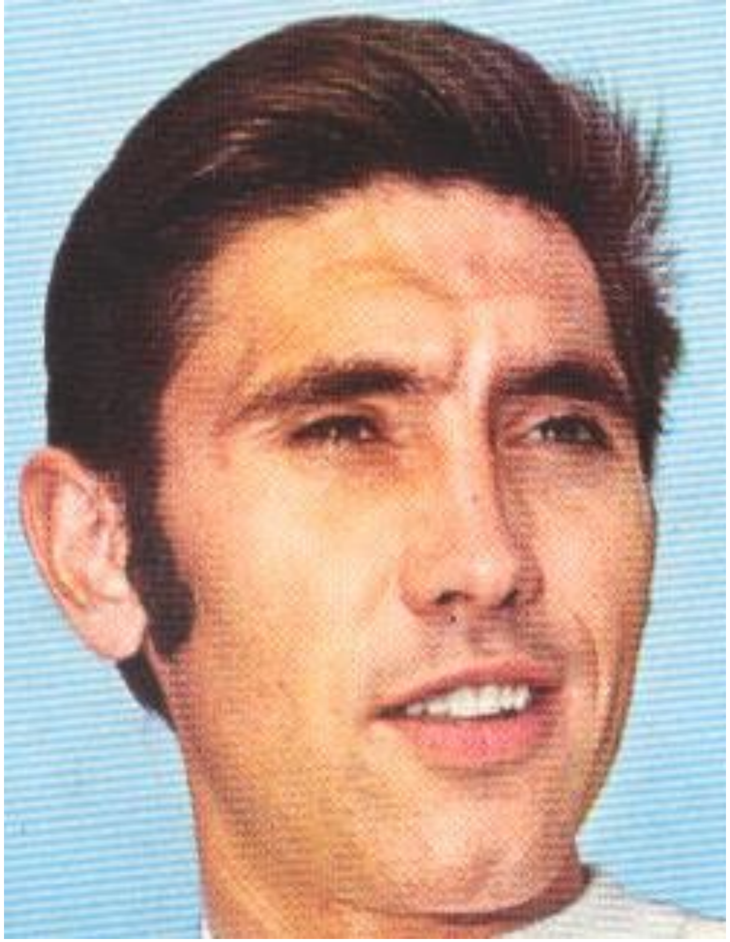

1. EDDY MERCKX 2. JO DE MEYERE 3. LUK ALLOO

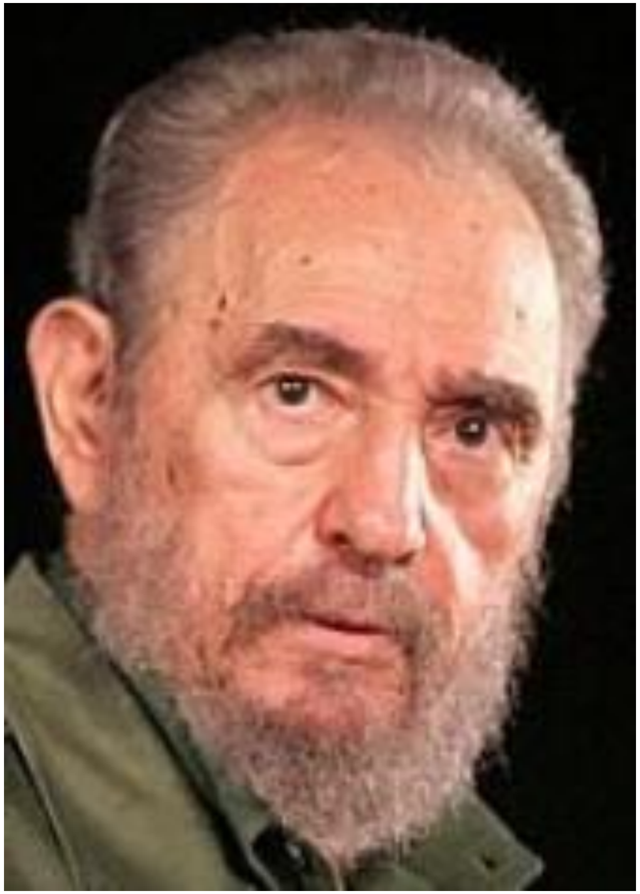

1. YASSER ARAFAT 2. OSAMA BIN LADEN 3. FIDEL CASTRO

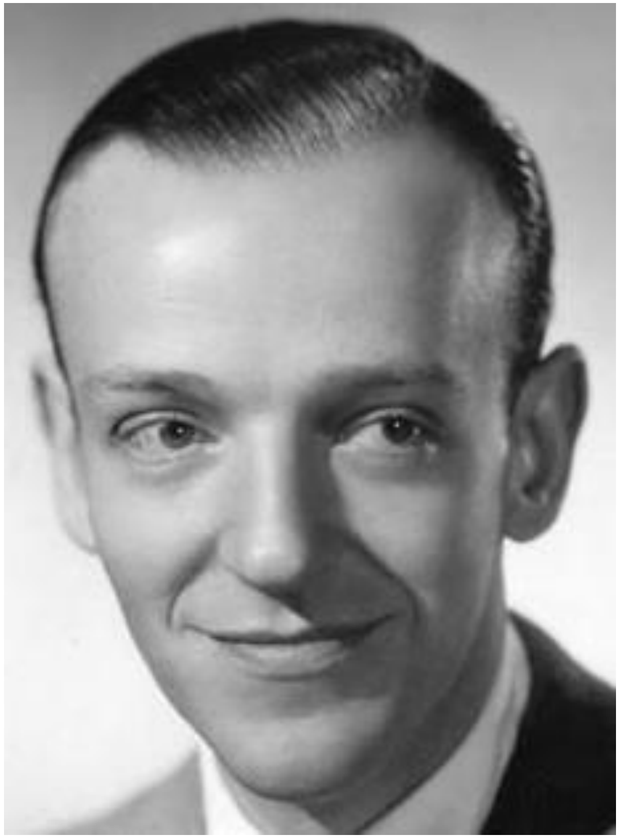

1. JAMES DEAN 2. FRED ASTAIRE 3. JAMES LAST

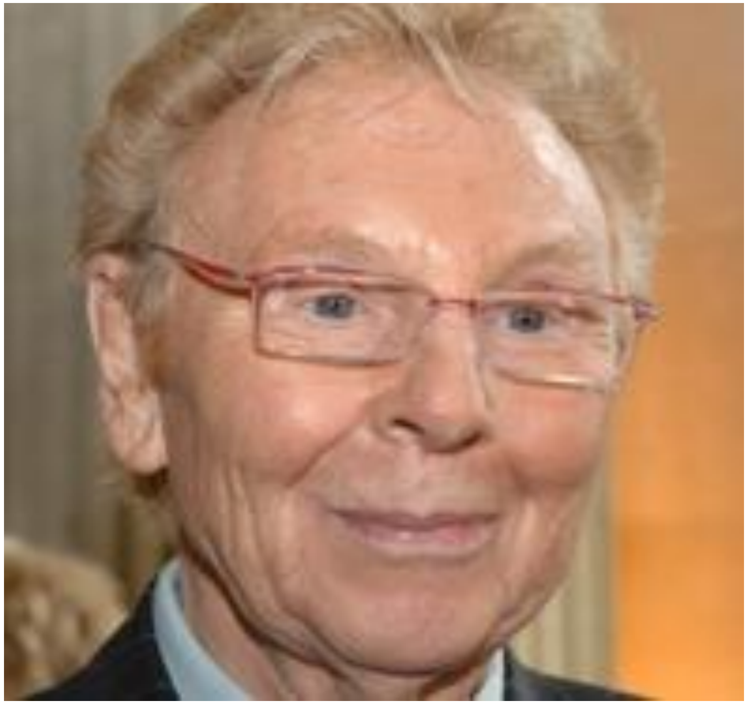

1. GASTON BERGHMANS 2. VADER ABRAHAM 3. WILLY VANDERSTEEN

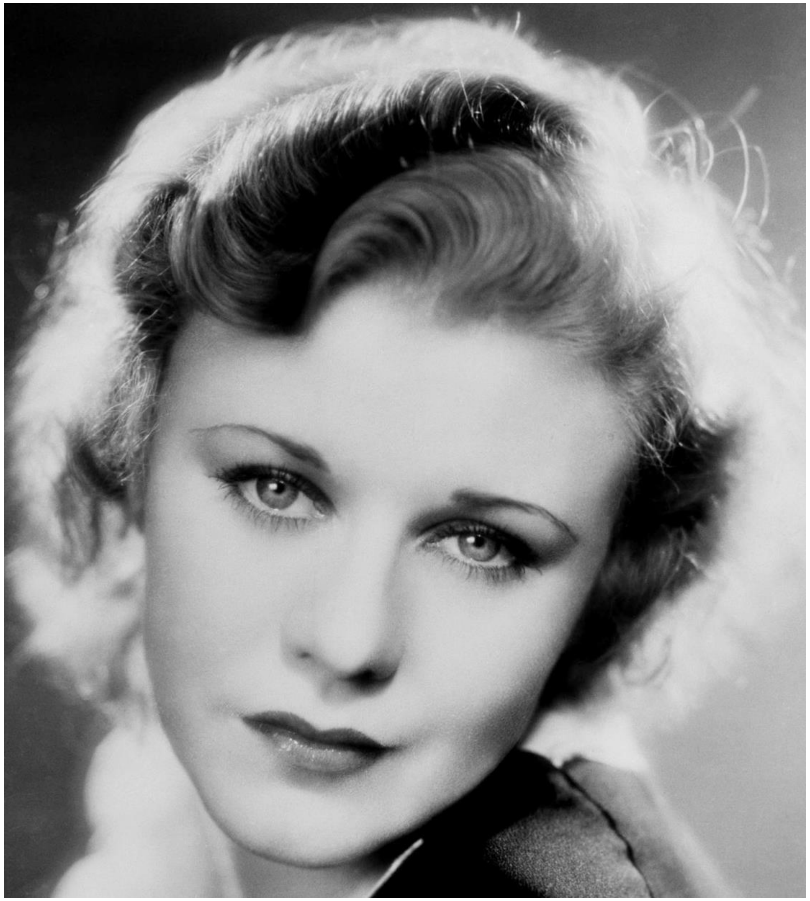

1. DOLLY PARTON 2. BRIGITTE BARDOT 3. GINGER ROGERS

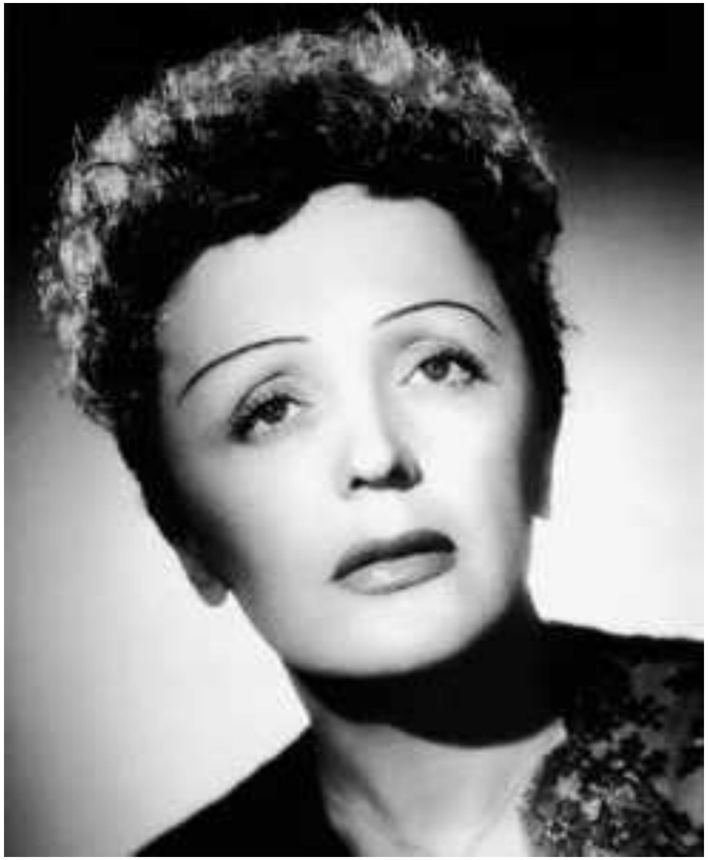

1. MIREILLE MATHIEU 2. EDITH PIAF 3. MARGARET THATCHER

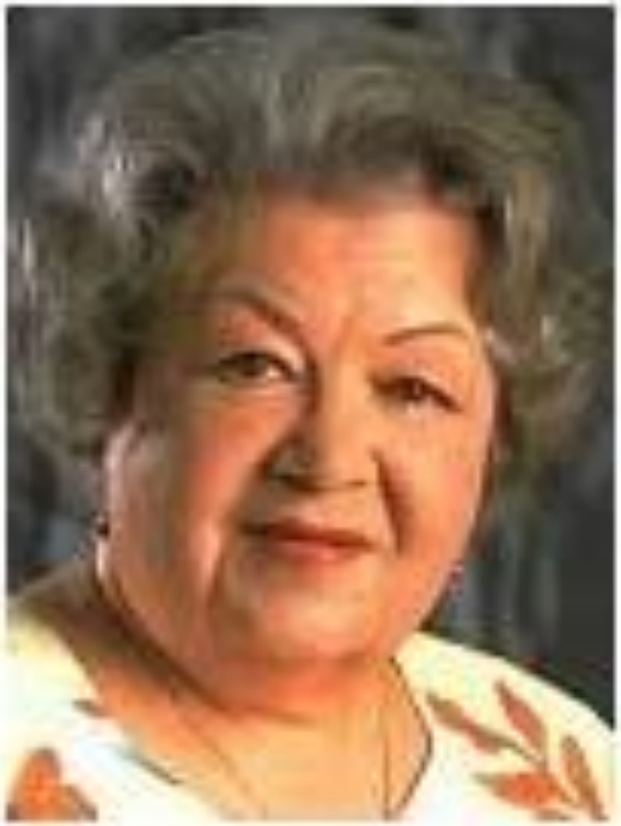

1. ANN PETERSEN 2. DENISE DE WEERDT 3. JET JORSSEN

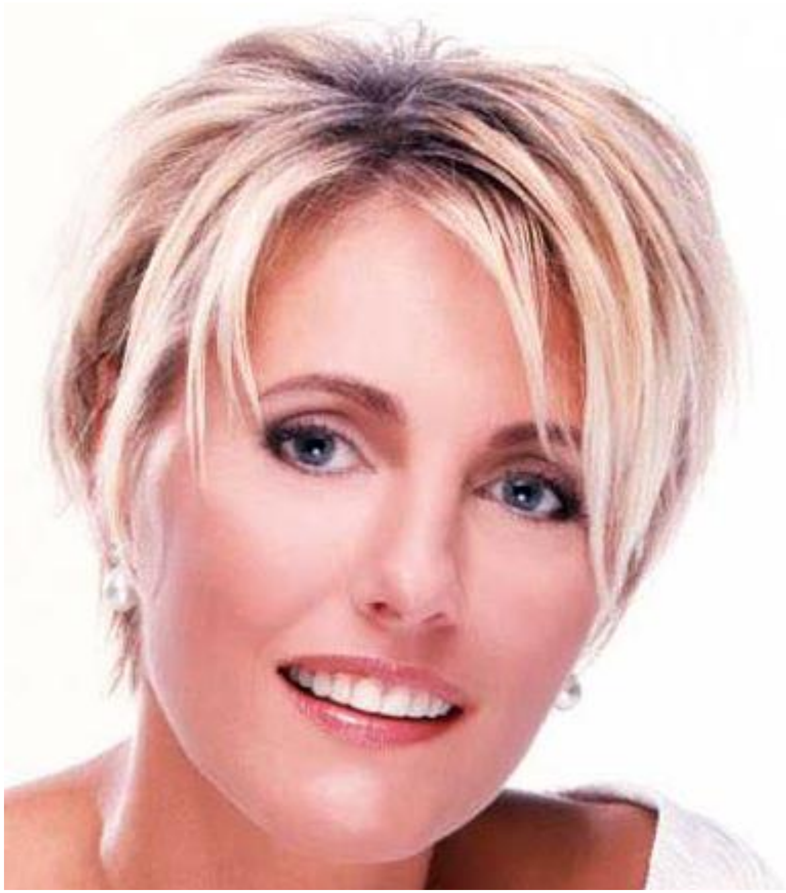

1. JANINE BISSCHOPS 2. MARIANNE THYSSEN 3. DANA WINNER

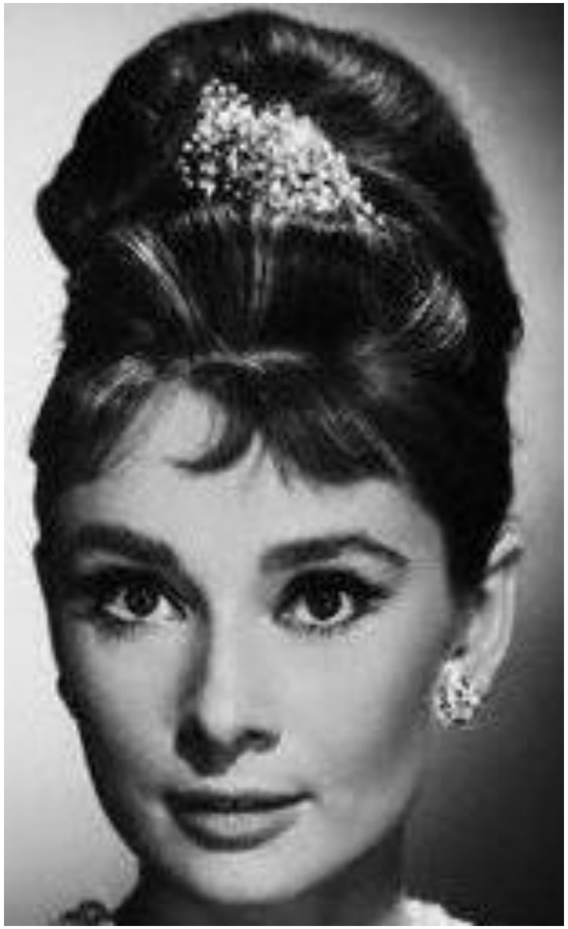

1. INGRID BERGMAN 2. AUDREY HEPBURN 3. JUDY GARLAND

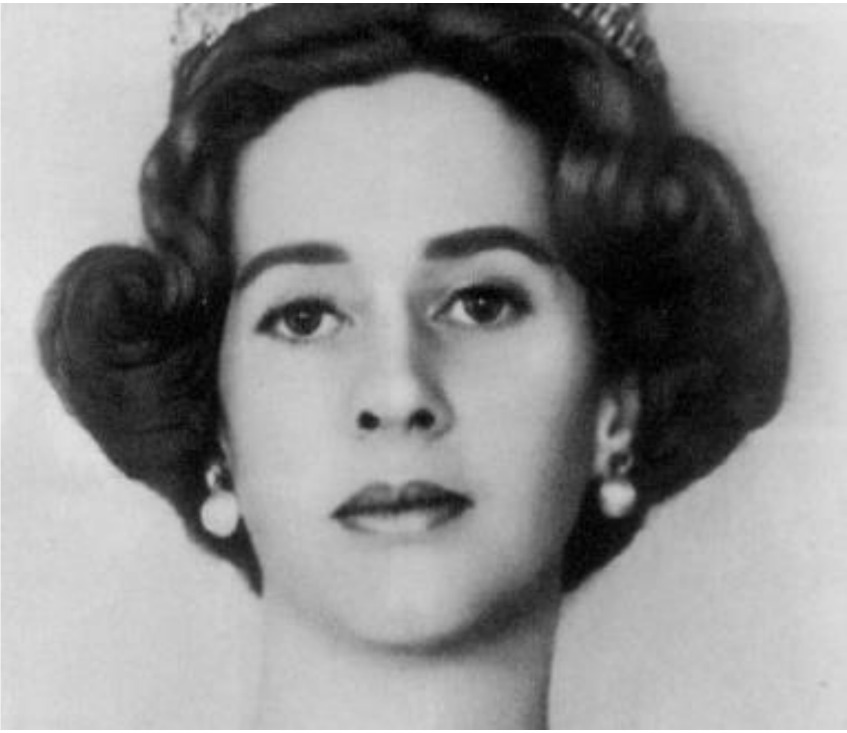

1. KONINGIN FABIOLA 2. KONINGIN PAOLA 3. PRINSES MATHILDE

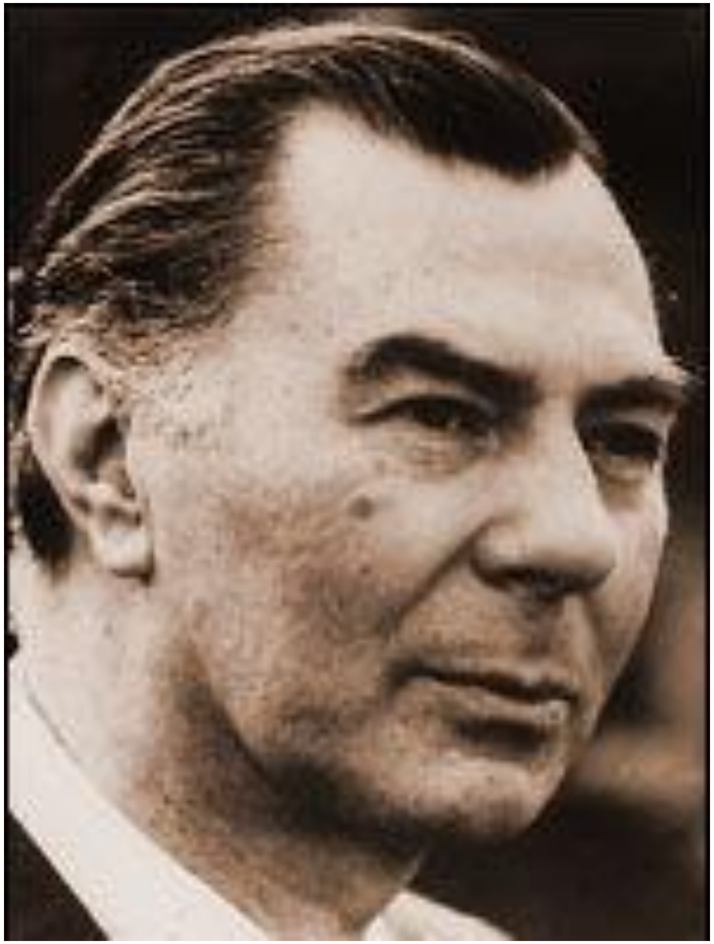

1. JEAN-LUC DEHAENE 2. WILLEM ELSSCHOT 3. LEO TINDEMANS

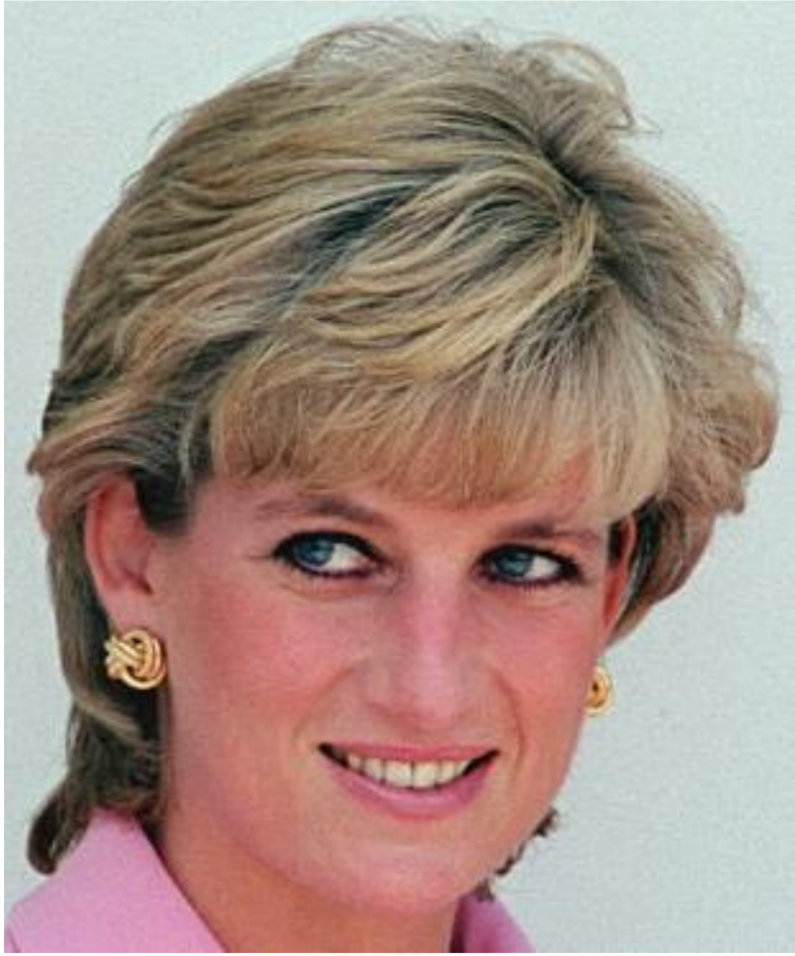

1. LUCILLE BALL 2. PRINCESS DIANA 3. QUEEN ELIZABETH

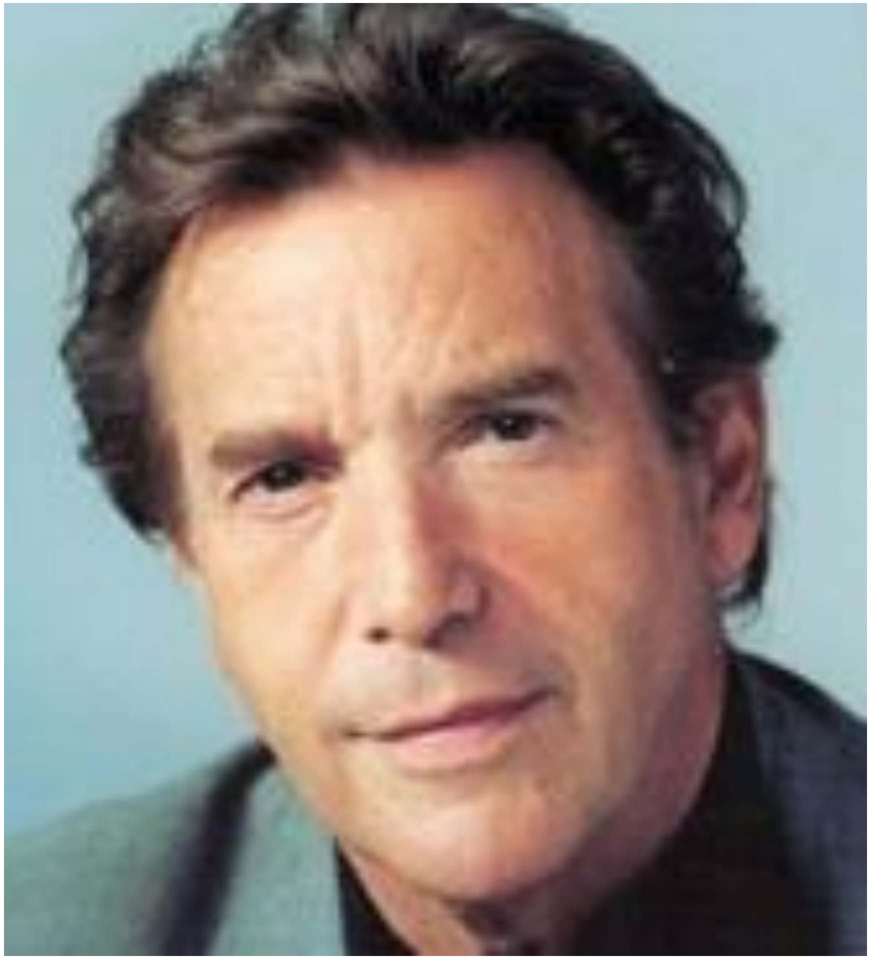

1. WILL TURA 2. GODFRIED BOMANS 3. BART DE WEVER

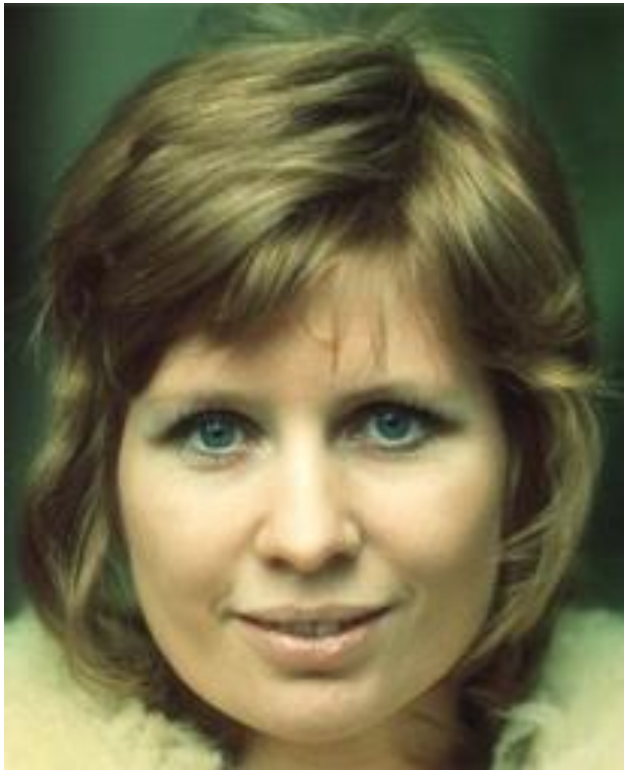

1. REGINA CLAUWAERT 2. RACHEL FREDERIKX 3. AN CHRISTY

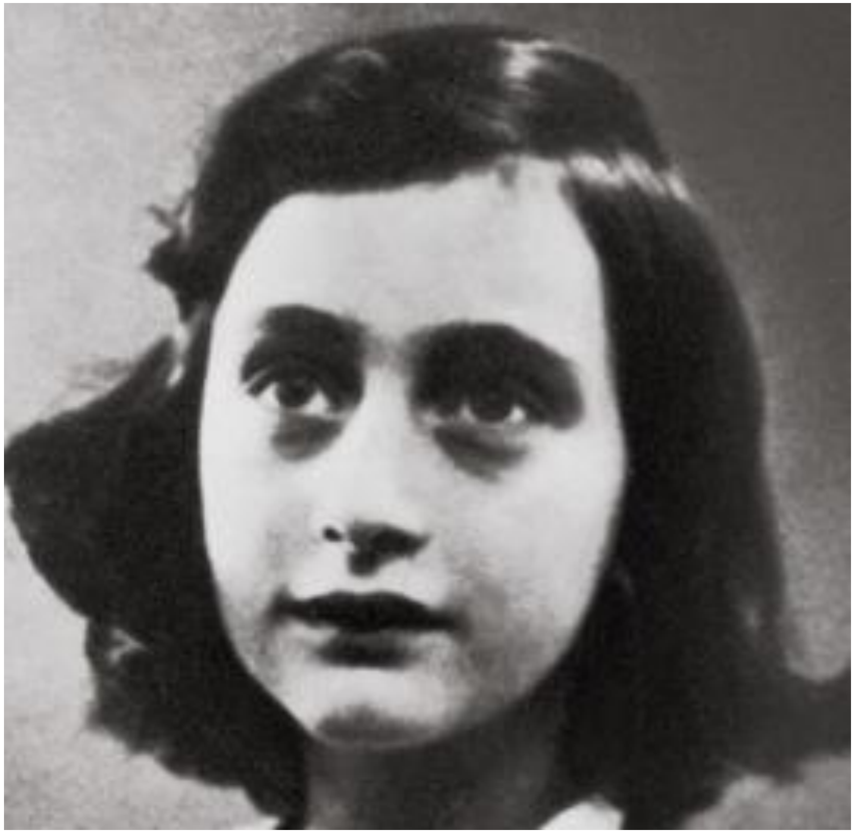

1. MARIE CURIE 2. ANNE FRANK 3. AGATHA CHRISTIE

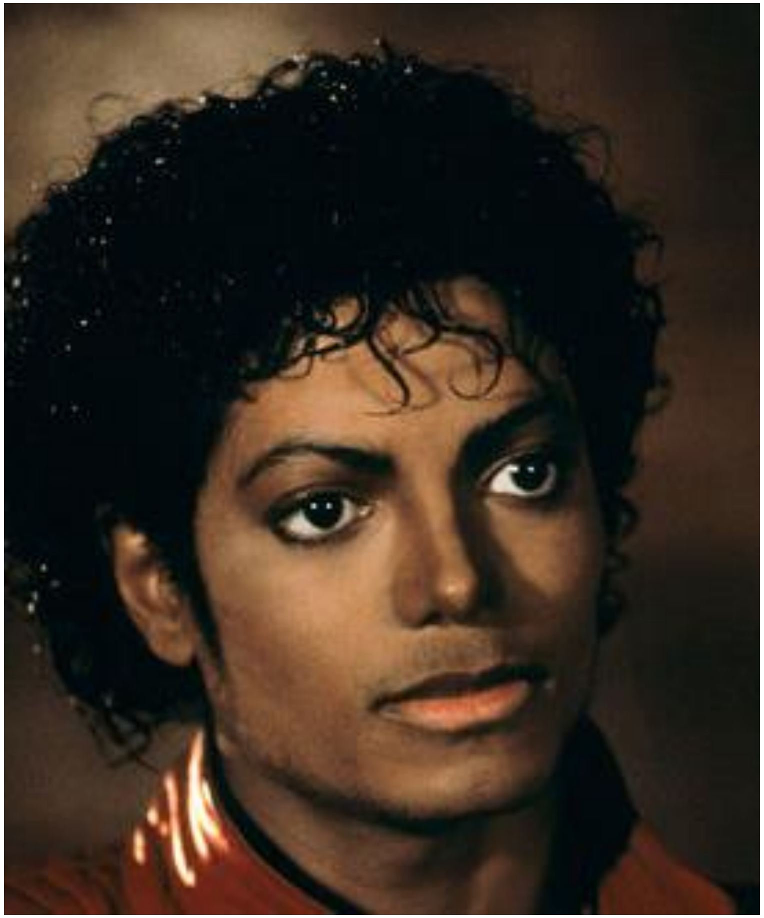

1. MICHAEL JACKSON 2. MOHAMMED ALI 3. RAY CHARLES

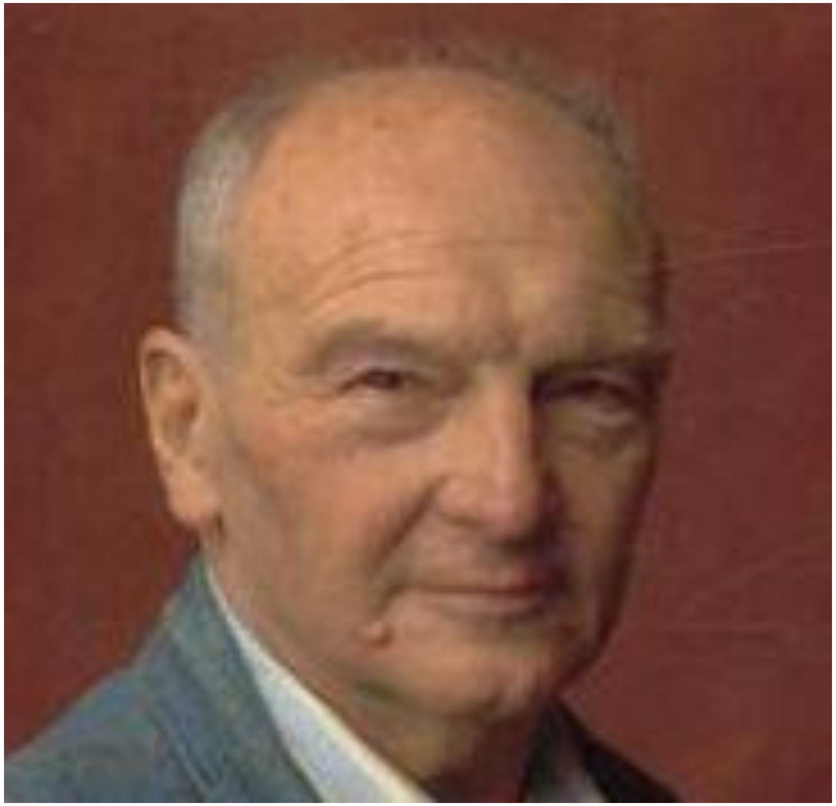

1. NONKEL BOB 2. HUGO CLAUS 3. LUC PHILIPS

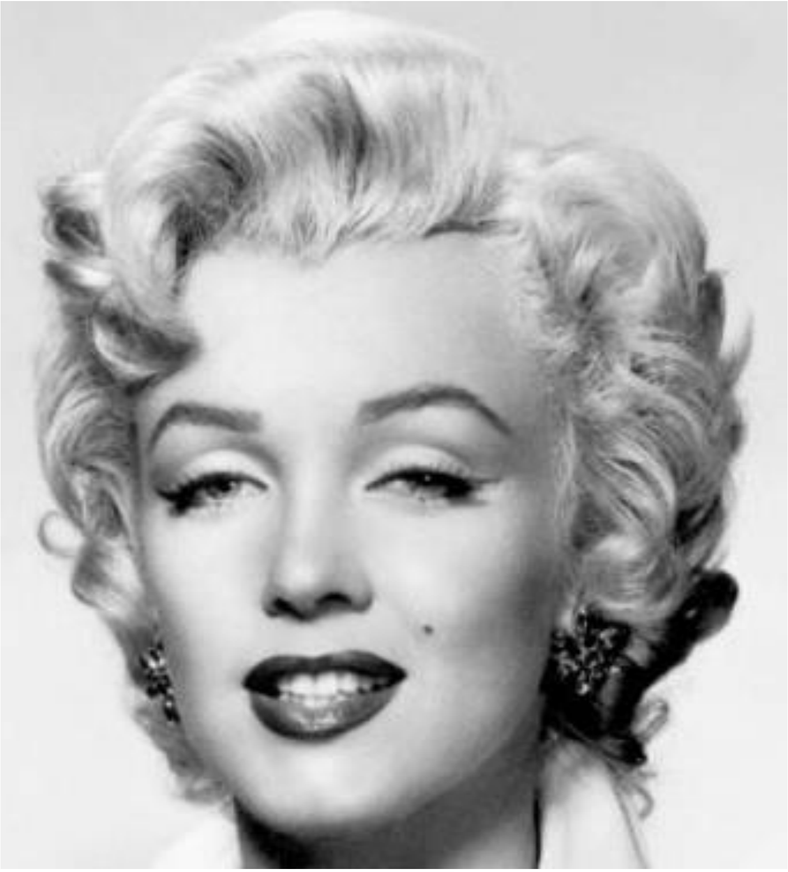

1. COCO CHANEL 2. MARILYN MONROE 3. DORIS DAY

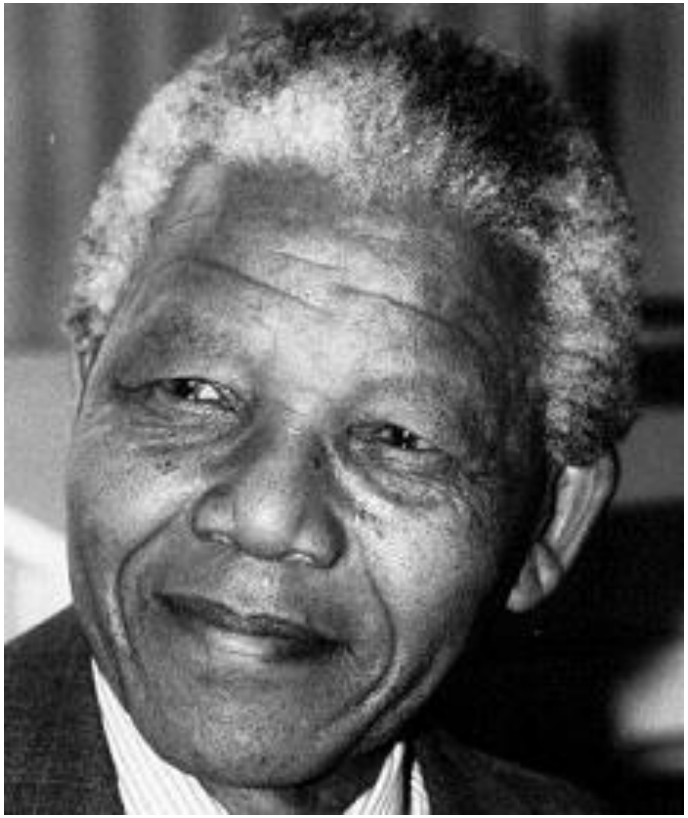

1. NELSON MANDELA 2. MOBUTU 3. MARTIN LUTHER KING

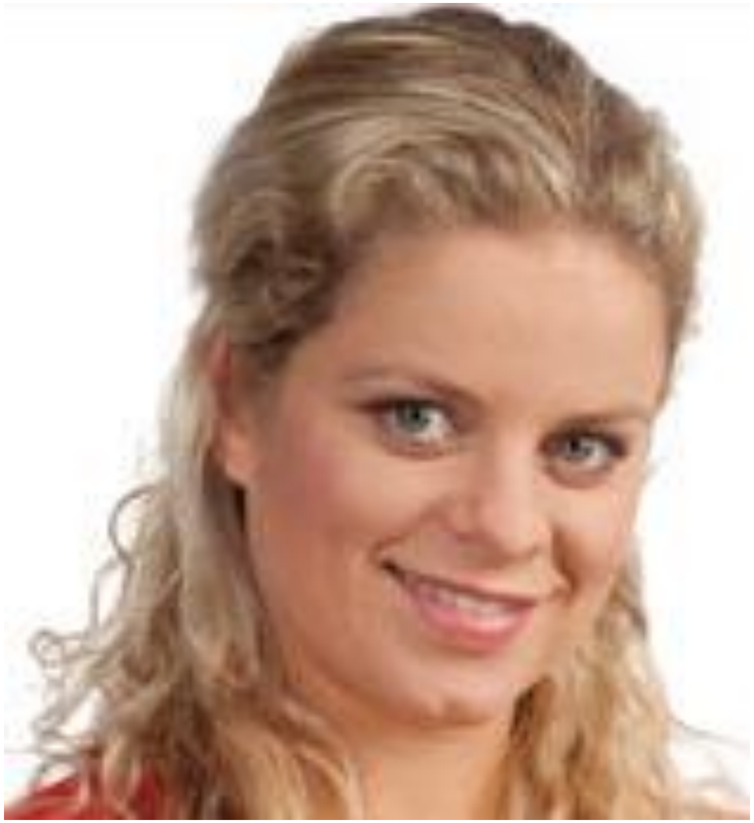

1. JUSTIN HENIN 2. TIA HELLEBAUT 3. KIM CLIJSTERS

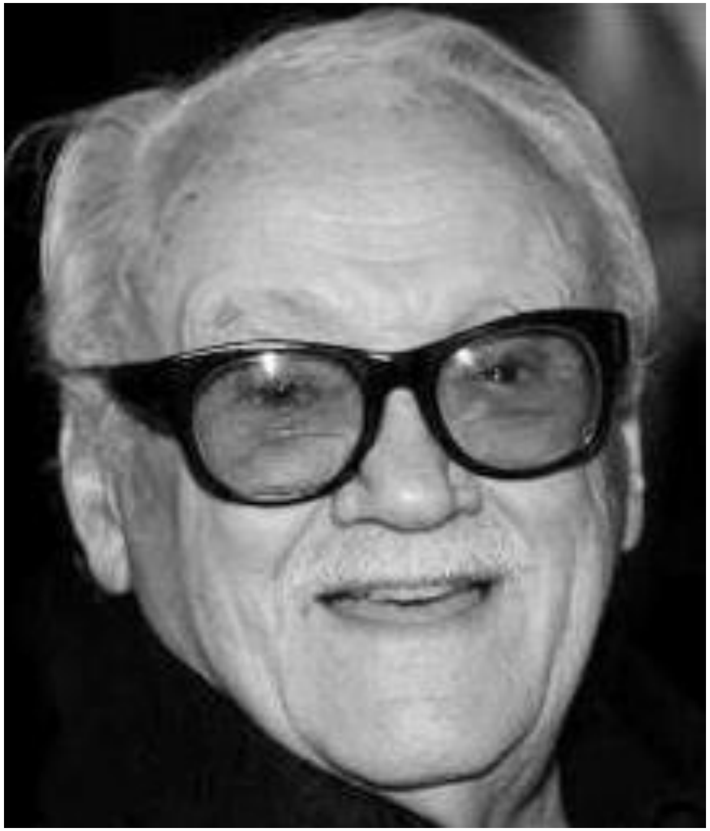

1. PATER DAMIAAN 2. TOOTS THIELEMANS 3. NAND BUYL

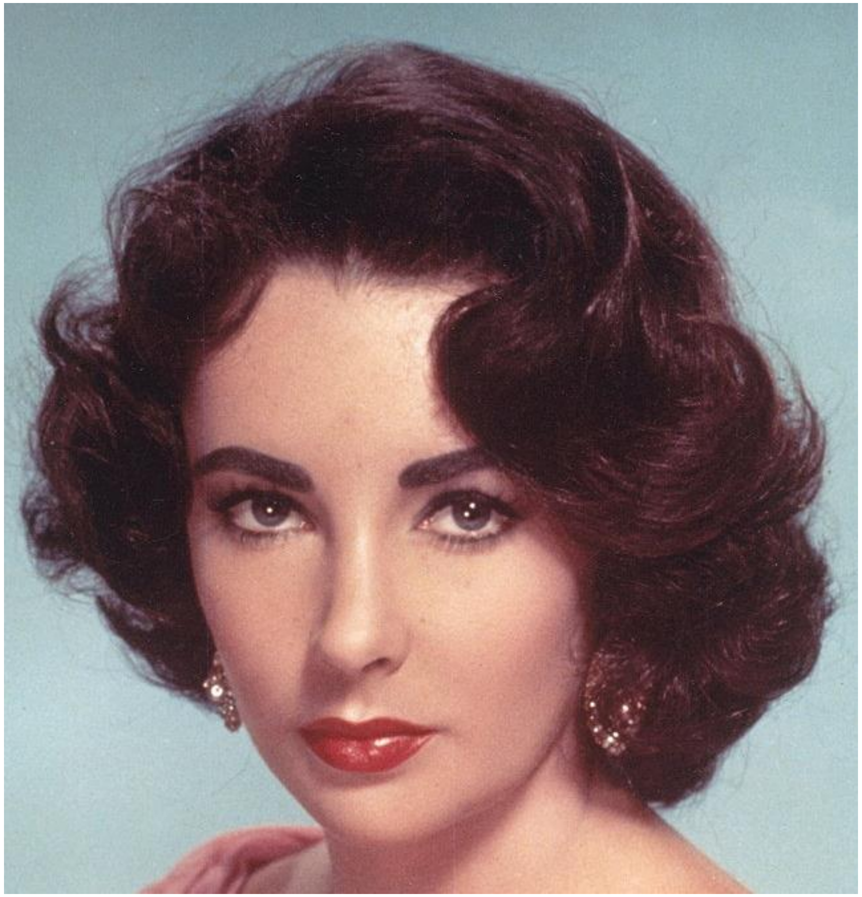

1. ELIZABETH TAYLOR 2. ARETHA FRANKLIN 3. TINA TURNER

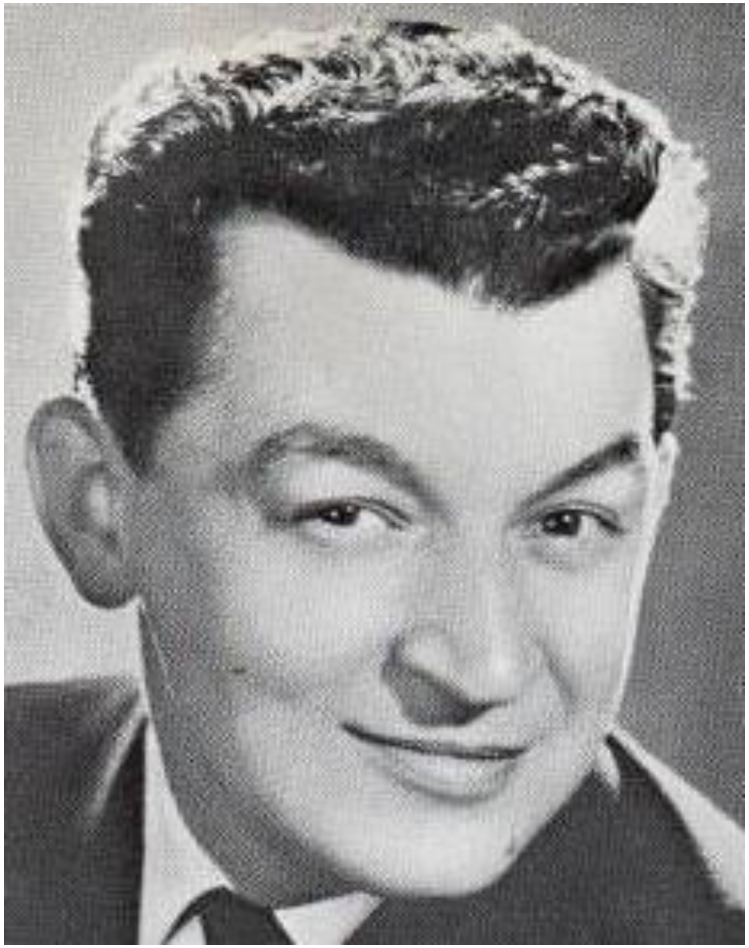

1. YVES LETERME 2. PAUL JAMBERS 3. TONI CORSARI

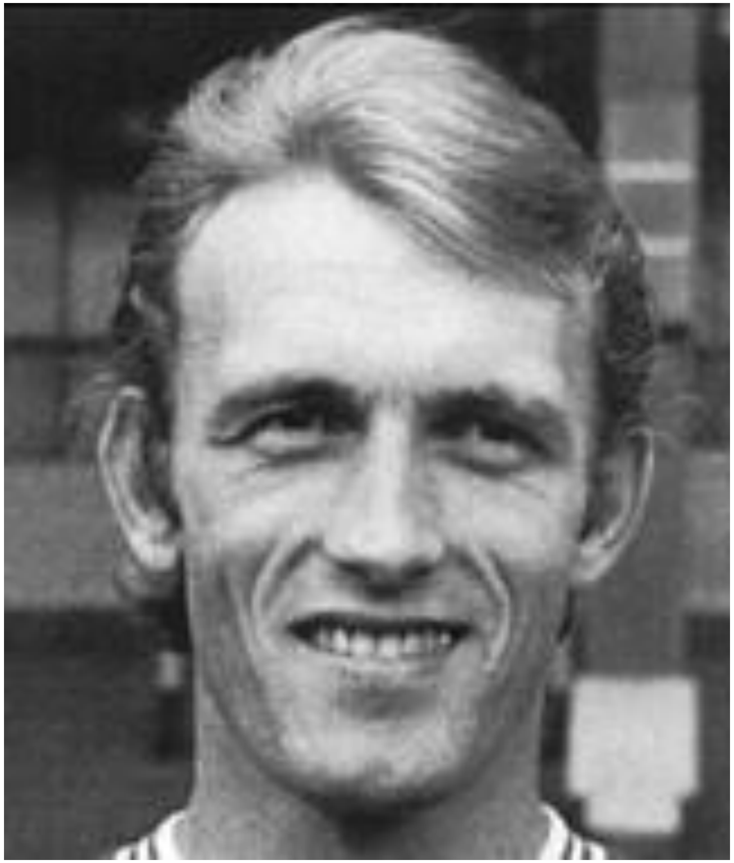

1. PAUL VAN HIMST 2. RIK VAN STEENBERGEN 3. URBANUS

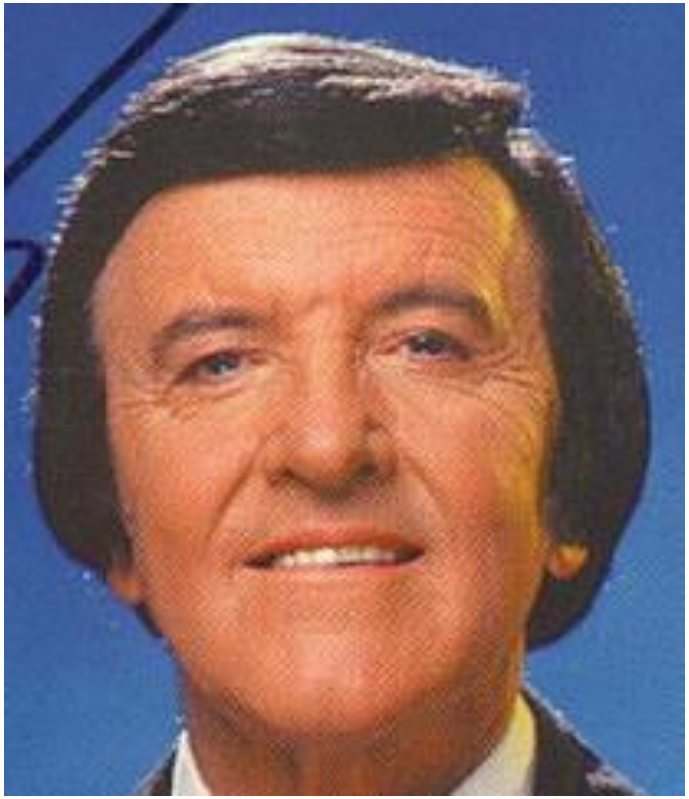

1. EDDY WALLY 2. WALTER CAPIAU 3. HERMAN VAN MOLLE

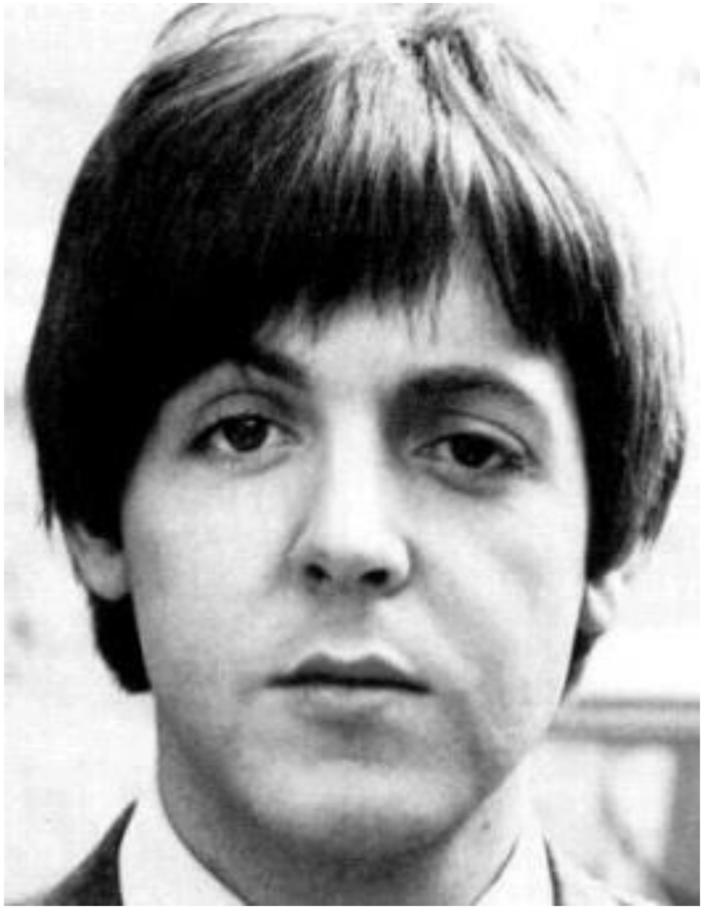

1. ROALD DAHL 2. RINGO STARR 3. PAUL MCCARTNEY

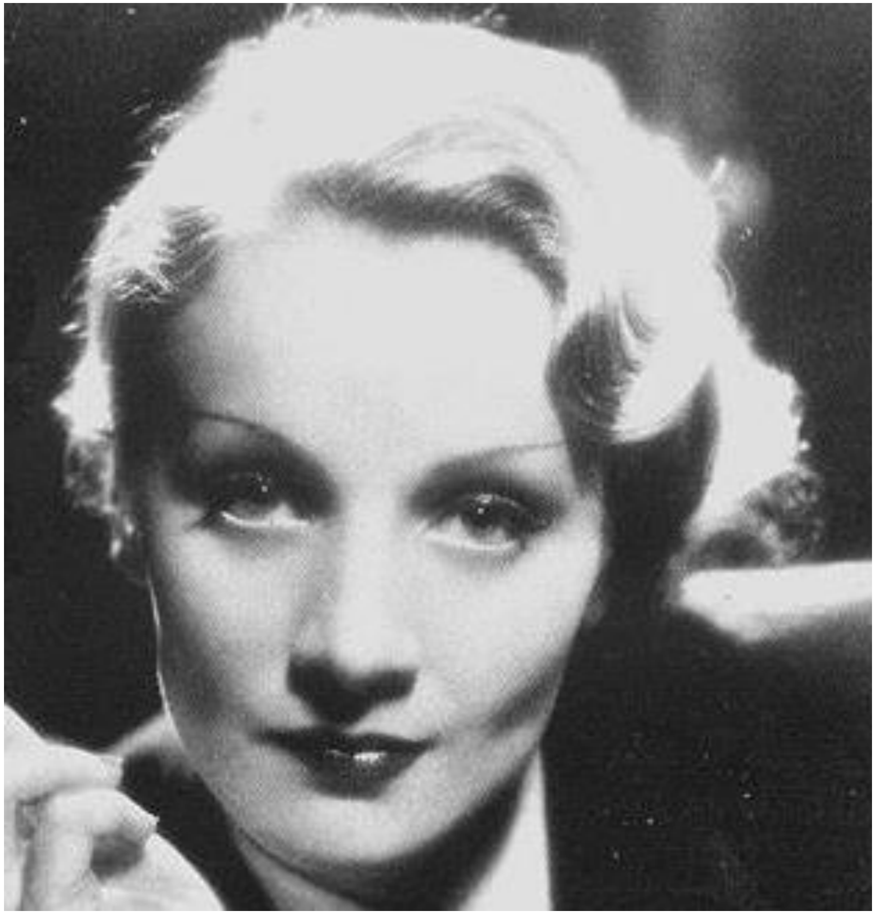

1. GRACE KELLY 2. MARLENE DIETRICH 3. EMILY DICKINSON

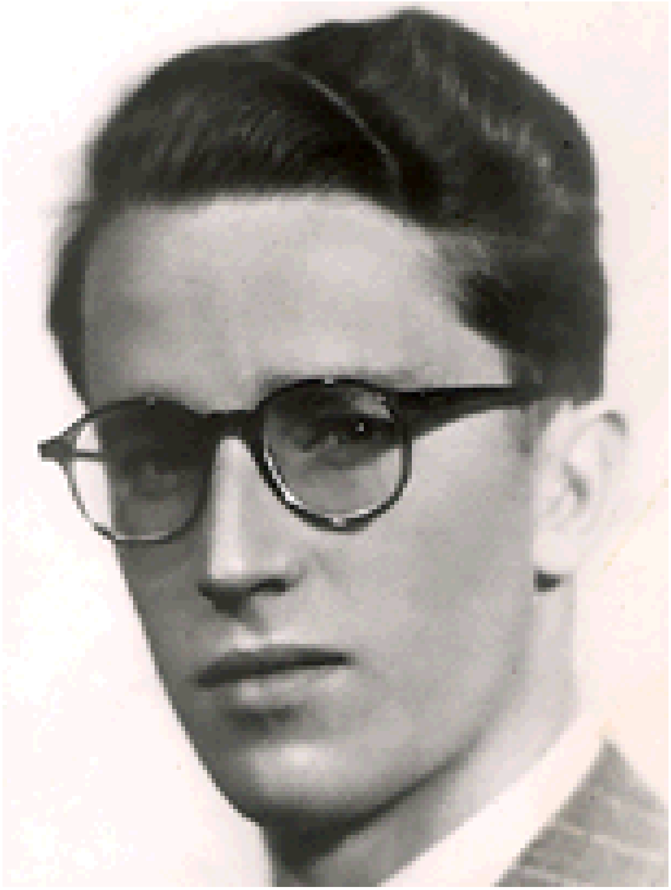

1. KONING BOUDEWIJN 2. BOUDEWIJN DE GROOT 3. HELMUT LOTTI

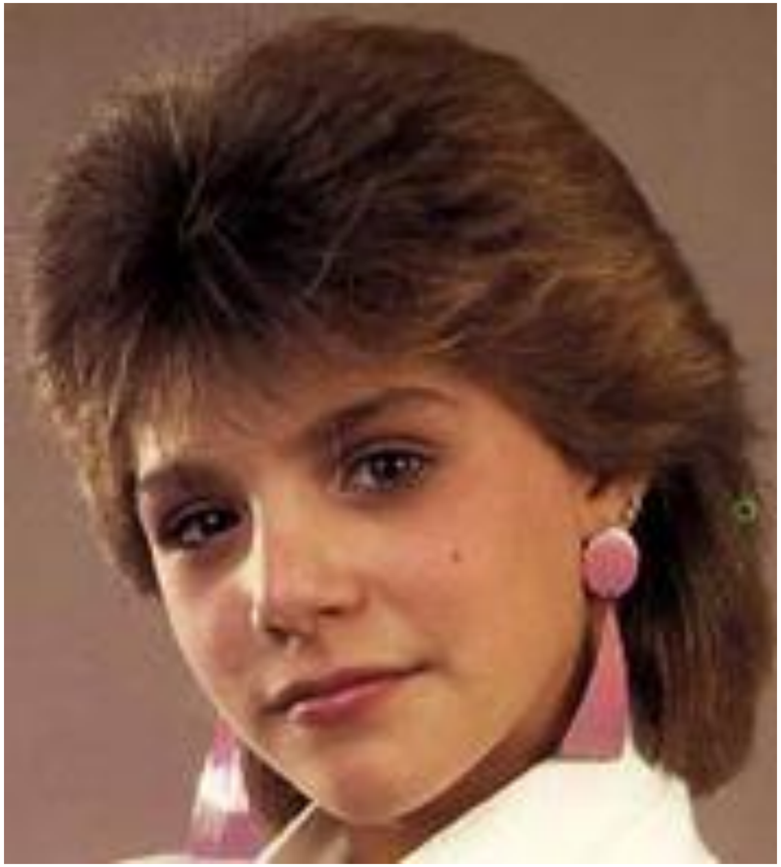

1. GERTY CHRISTOFFELS 2. LILIANE SAINT PIERRE 3. SANDRA KIM

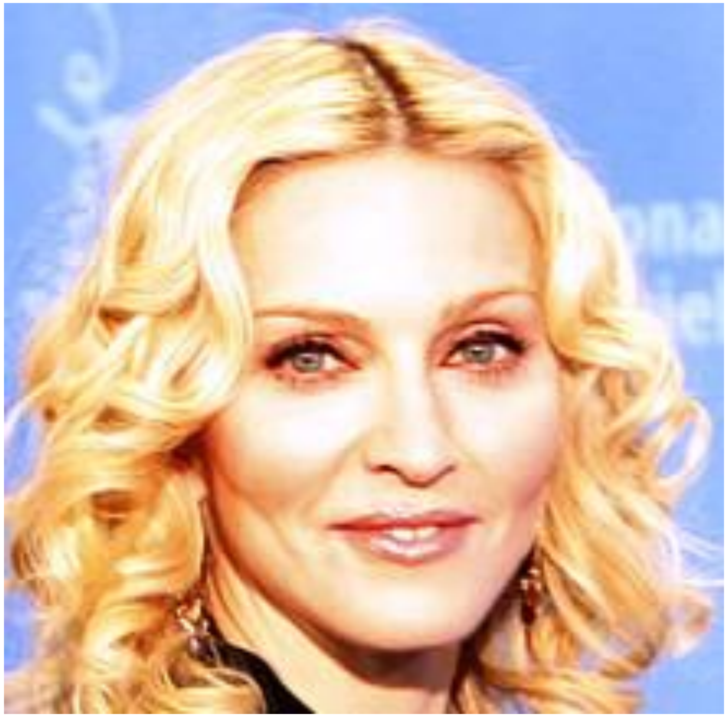

1. MADONNA 2. CHER 3. CELINE DION

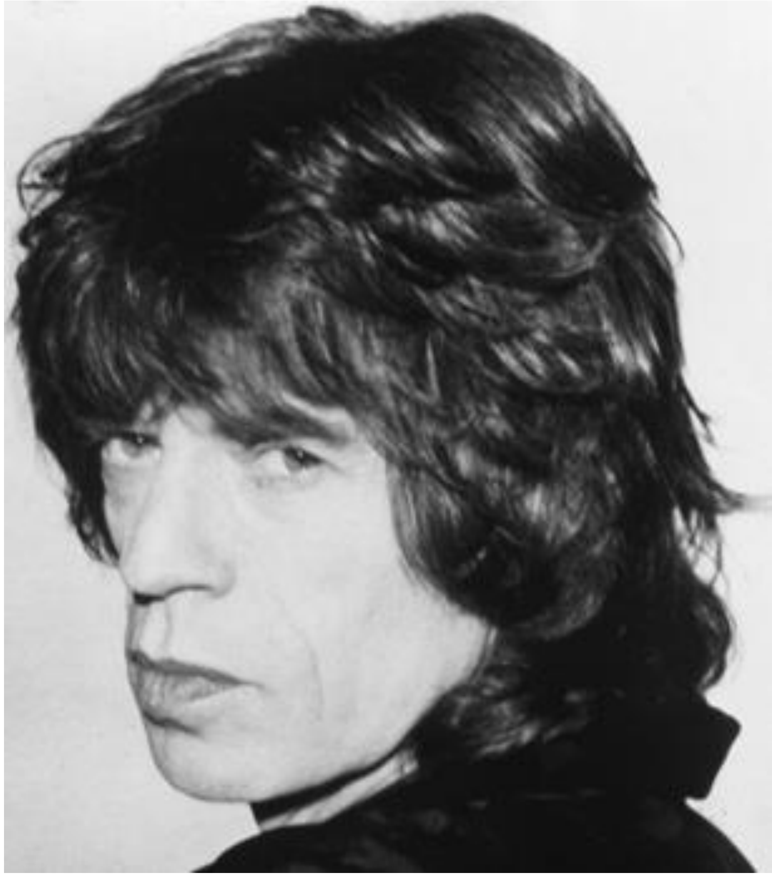

1. MICK JAGGER 2. AL PACINO 3. JOHN CLEESE

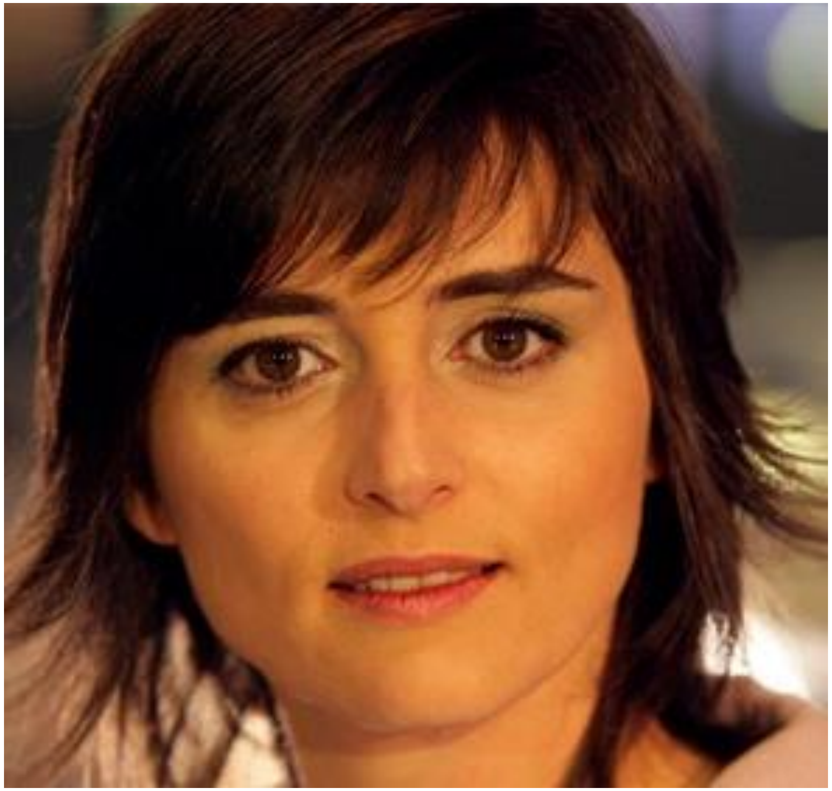

1. YASMINE 2. GEENA LISA 3. ISABELLE A
